# Ethnobotany of dye plants in Southern Italy, Mediterranean Basin: floristic catalog and two centuries of analysis of traditional botanical knowledge heritage

**DOI:** 10.1186/s13002-020-00384-2

**Published:** 2020-06-03

**Authors:** A. Prigioniero, A. Geraci, R. Schicchi, M. Tartaglia, D. Zuzolo, P. Scarano, M. Marziano, A. Postiglione, R. Sciarrillo, C. Guarino

**Affiliations:** 1 Department of Sciences and Technologies, University of Sannio, Via De Sanctis snc, 82100 Benevento, Italy; 2 Department of Agricultural, Food and Forestry Sciences, University of Palermo, Viale delle Scienze, Ed, 490128 Palermo, Italy

**Keywords:** Ethnobotany, Dye plants, Mediterranean Basin, Database

## Abstract

**Background:**

Since ancient times, man has learned to use plants to obtain natural dyes, but this traditional botanical knowledge (TBK) is eroding. In the late, during, and the early 1800s, there was an increase in research related to dye species, and this allowed the development of industry and economy in rural contexts of Southern Italy. Today, dyes are mainly obtained from synthetic products, and this leads to risks for human health related to pollution.

**Methods:**

Starting from the literature, three catalogs of the dyeing species (plants, algae, fungi, and lichens) used in the Mediterranean Basin and mainly in Southern Italy have been created. Percentages of parts used and colors extracted from species have been recorded and analyzed. The plant species present in the catalogs have been verified in the territories of Southern Italy, and the data have been registered. An ethnobotanical survey was conducted, in the region of Southern Italy, to verify the erosion level of traditional botanical knowledge, linked to the ethnobotanical dyeing, over time.

**Results:**

A total of 524 species were recorded among plants, algae, fungi, and lichens, and related parts used and extracted pigments. Most uses concern the stems and leaves, and the most frequent color is yellow. From the on-field survey operations, 283 plant species have been verified. These represent 64.31% of the species reported in the flora of the dye plants produced. The results, from the ethnobotanical survey, show that only 8.6% of TBK remained in the collective memory.

**Conclusions:**

This catalog is among the largest in this sector and is the basis for studies related to the restoration of an eco-sustainable economy which would allow the development of marginal areas present throughout Southern Italy.

## Background

The art of dyeing with natural colors extracted from plants has its origins at the dawn of civilization, when man had not yet acquired the writing skill [1]. In ancient times, color was contemplated as a spiritual necessity not less important than the physical need of food. Ever since the dawn of civilization, mankind has shown his liking and attraction of colors, as fundamental tools to communicating one’s belonging to a social class or tribe and to propitiate the forces of nature or those of the gods [2]. Even in prehistoric days, when men still lived in caves, available natural dyestuff and pigment were used for coloring animal skin and their own skin during festivals as well as during wars. Men believed that the color would give them magical powers, protect them from evil spirits, and help them to achieve victory in war [2–5]. With dyeing, man has always tried to imitate the colors of nature, deified, and considered the maximum expression of beauty [6]. The development of dyeing art and the search for raw materials follow the development of civilization. In prehistory, organic dyes and red, brown, yellow, black, and white mineral pigments were used, while minor are the evidences of the use of green and blue dyes that were complex to reproduce [5]. Studies carried out on archeological finds have shown the use of many plants to derive colors, such as yellow from *Arbutus unedo* L. or from *Cotinus coggygria* L., red from *Atriplex hortensis* L. and from *Rubia tinctorum* L., the orange from *Galium verum* L., and blue from the berries of *Sambucus nigra* L. [3, 7]. Black was also obtained using ground coal and has been attested the use of the blue color, coming from woad (*Isatis tinctoria* L.) [4]. This testimony is of great interest, considering the difficult process of extraction of indigo from leaves, for which it is necessary to ferment these, add an alkaline substance (vegetable ashes or urine), and then have the fabric oxidized to see the color [8]. Evidence of naturally dyed materials dates to the Egyptians and Phoenicians, and Greeks and Romans developed good techniques for dyeing [9]. With trade in the Mediterranean, the dyeing techniques introduced by the Greeks underwent further development. They got the blue from the woad, red from madder, and met many mordants, of which the most popular was the potassium alum. The Romans initially used tunics and cloaks of wool in their natural color. The dyeing activity contributed to the development of the Roman economy. With the development of trade and territorial conquests, the knowledge of dyeing practices in use by other peoples was also studied, until the achievement, in the imperial period, of the monopoly of some dyes such as purpura from *Bolinus brandaris* L., destined only to the richest classes [6]. Intense madder plantations were also developed, and the so-called *Collegium tinctorium* gained importance, whose members were divided on the base of the color with which they dyed: “Flaminii” for orange, “Violarii” for violet, “Crocei” for yellow, “Porporarii” for purple [6]. An important vision of the art of natural dyeing also comes from the archeological evidence of Magna Grecia (Paestum) and the remains of Pompeii from the Roman era. The fabrics, craft objects, and paintings have been kept their elegance and splendor, even after several centuries [5, 10]. Early trade routes introduced new plant dyes from India, Turkey, and the Orient [9]. During the Middle Age, there was a great development of the cultivation of plants from which fibers were obtained (flax, hemp, and cotton); of the processing of animal hair, in particular wool; and of the art of silk production by the silkworm breeding [1]. This led to the opening of the first real fabric factories; at the same time, the dyeing art was developed and it was during the Middle Ages that the first dyeing corporations and the first specialized texts were born. By the 10th century, the dye craft was flourishing all over Europe [1, 9]. The popularity of natural dyeing continued to spread for several centuries; an Italian book published in 1540 contained over 200 dye recipes. This text, published by Giovanventura Rosetti in Venice between the end of the Middle Ages and the Renaissance, is entitled *Plictho de l’arte de Tentori*. The treaty provides detailed information both on the dyes used on dyeing methods on wool, cotton, and linen and reports numerous dyeing recipes, anticipating modern dyeing chemistry [3, 9]. During the dynasty of the Bourbons (1732–1860), in Italy, within the Kingdom of the Two Sicilies, there was a noticeable intensification of industries linked to textile manufacturing. The silk factories of San Leucio (CE) became famous in the world because of the manufacturing production of high-quality silks dyed with pigments of natural origin. This strong impulse to textile manufactures, linked to silk, wool, and cotton, caused a strong demand for pigments within the Kingdom. In relation to this event, the Bourbons commissioned some botanists, Tenore first and Briganti subsequently, experimenting with spontaneous plants for dyeing. Bourbons were responsible for restoring the cultivation of *Isatis tinctoria*, which in the previous century had been gradually replaced, in use, by *Indigofera tinctoria* L. [8]. In this context, the famous factories of blue extraction from the woad, adjacent to the Real Site of Carditello, and the famous plantations of sumac at the Royal Palace of Caserta and Capodimonte, were born. All the Bourbon sites, located throughout the kingdom for quite long periods, hosted plantations of dye plants, and many sites became centers of experimentation with new pigments extracted from plant species. This condition contributed to the development and maintenance of peculiar dyeing traditions, in many centers of Southern Italy, such as the dyeing of wool at the village of Cerreto Sannita (Benevento, Italy) using walnut husks (*Juglans regia* L.). Unfortunately, natural dyes are rarely used in modern dyeing, except by specialist companies and craft dyers [11]. For the production of eco-textiles based on natural dyes, an evaluation of the economic and ecological sources for natural dyes is of growing interest [1, 11]. The use of dyeing plants and their reintroduction as potential natural resources to be cultivated, recently, have been favored, in Europe, by orientations of the common agricultural policy. Among the aims of these policies, there is to encourage forms of agriculture addressed not only to the production of food, but also to other forms of eco-sustainable production [1]. This new interest is motivated by several factors: environmental problems related to the production, application of dyes and disposal of wastewater, the risks for the health of workers and consumers towards production, handling and contact with synthetic dyes, market interest in natural and ecological textiles, and finally, the possibility of creating new sources of income for agricultural enterprises, by inserting dye plants within crop alternatives, with benefits linked to environmental, social, and environmental sustainability and economy of the rural sector [1]. The interest in the use of natural dyes for productive and economic purposes has led to an increase in investments in the textile sector. This has been observed recently and reported in international events [7]. This overview highlights the importance and urgency of intensifying scientific and interdisciplinary research in all potential sources and aspects of natural dyes to optimize their promotion and their use as a source of alternative and sustainable income. The authors, in this work, want to promote the traditional botanical knowledge (TBK) of Southern Italy in the use of dyeing plants. The aim of the work is to recover and fix the ethnobotanical cultural heritage linked to the use of dye plants in the Mediterranean context of Southern Italy through the creation of a floristic catalog as complete as never before. The data were obtained from archival, iconographic, ethnobotanical, and ethno-anthropological researches. In addition, over the course of 2 years, from 2016 to 2018, the presence of dye plants in the area of interest were checked, and an ethnobotanical survey in Southern Italy was performed.

## Methods

### Archive, bibliographic, and archeological sources

Archival research under the heading Dyeing Plants involved primary sources held in the State Archives of Naples at the Bourbon Royal House and the floristic lists of the Botanical Gardens of Naples and Palermo [12–14]. Scientific production that reviewed regional contexts related to rural culture [8, 15–24] has been also analyzed. In addition, the archeological remains found in archeological sites of Southern Italy, which document fabrics, objects, and paintings colored with plant-derived pigments, have been considered [5, 10, 25, 26].

### Flora of dye plants

A flora of dye plants (Supplementary file 1) has been created starting from the analysis of the flora produced by Briganti [12] and other works of the seventeenth to nineteenth century [13, 14]. Species reported have been updated in the nomenclature using the Catalogue of Life: 2019 Annual Checklist [27]. Information reported on this flora are as follows: species name [27], family name [27], habitat and Italian locality where the species can be found or where they have been reported, altitudinal range and chorology [28], chromosome numbers (if known) [28, 29], pigment color, and pigment sources [12]. Data relating to the pigments, colors, and parts of the plants used have been studied and reformulated to adapt to the current technical-scientific language. Species are listed in alphabetical order to allow an easier search of the items on the list. The frequency of the species on the Italian territories is reported with RR (very rare), R (rare), C (common), and CC (very common) [28].

### Plants, algae, lichens, and fungi catalogs

A floristic catalog of dyeing plants (Supplementary Table 1) has been produced, reporting some information present in the Flora and adding, where found, further recent bibliographic references relating to the species included in the table and their dyeing use [1, 2, 6, 7, 9, 11–18, 21–24, 30–39]. Records on the table are ordered according to the criteria of the Angiosperm Phylogeny Group (APG) IV to add an information level, linked to the phylogeny of the species concerned [40]. In the literature, citations concerning mushrooms, lichens, and algae used for dyeing purposes have been founded [12, 22, 24]. This information has been reported in two catalogs, one concerning fungi and lichens (Supplementary Table 2) and the other concerning algae uses as dye sources (Supplementary Table 3). The tables show the names of the species updated according to the current nomenclature [27], the families to which the species belong [27], the resulting colors, and the part of the organism used to derive the pigments [12, 22, 24]. Data relating to the pigments, colors, and parts of the organisms used have been studied and reformulated to adapt to the current technical-scientific language. The entries in the two tables are listed according to the alphabetical order of the families to which the species belong.

### Field studies

#### Floristic investigation of the cited species

During a 2-year period (from 2016 to2018), a floristic survey was carried out on the territory of Southern Italy and of the Major Islands (Sicily and Sardinia) to confirm the presence of dyeing species cited in the literature [1, 2, 6, 7, 9, 11–18, 21–24, 30–39]. The geographical information concerning the positions of the species reported in our catalog was found in the flora of Italy [28] and in the Prodrome of Italian Vegetation produced and updated by the Italian Botanical Society [41]. Species recorded for the regions of Southern Italy (Molise, Campania, Calabria, Puglia, and Basilicata) and for the Major Islands (Sicily and Sardinia) were taken into consideration. All the species actually found in the localities mentioned in the literature [28, 41] have been subsequently recorded. Herbarium vouchers have been taken from Herbarium of Palermo (PAL), Virtual Herbarium of Lake Van Basin (VHLVB), Linnean herbarium—Department of Phanerogamic Botany Swedish Museum of Natural History (S-LINN), Herbarium Luigi Paolucci (H.PAOL), New York Botanical Garden (NYBG), Herbarium Horti Botanici Pisani (PI), and Naturalis Biodiversity Center (NL). All the vouchers have been reported in the flora of the dyeing plants (Supplementary Materials 1).

#### Semi-structured interviews

A guide text has been produced for the realization of semi-structured interviews to be carried out on a sample of individuals. We used the snowball sampling technique to recruit a group of participants in each Southern Italian Region (Molise, Campania, Puglia, Calabria, Basilicata, Sicily, and Sardinia) [42]. The sample is composed of men (37%) and women (73%) (i) older than 70 years of age and (ii) resident from birth in the same villages of Southern Italy. In each region, a different number of interviews were carried out due to the availability of the interviewees and the different size of the population. In Molise, 57 interviews were carried out, in Campania 149 interviews, in Puglia 138 interviews, in Calabria 142 interviews, in Basilicata 58 interviews, in Sicily 134 interviews, and in Sardinia 153 interviews. The total number of interviews collected is 831. The semi-structured interview was preferable to any other form of investigation due to the level of education of the respondents (for 83% only elementary or lower) and for the possibility offered by the method of establishing a more empathic relationship conducive to dialog. The interview was conducted in Italian and anonymously. Respondents were asked if they remembered the following: (i) uses of plants to dye, (ii) which plants were used, and (iii) other people who were interested in plant dyes. Prior informed consent was verbally obtained before starting each interview, and ethical guidelines were rigorously followed [18]. The semi-structured interviews were listened to, and the non-interesting interviews for this work were discarded (when the interviewees claimed not to remember any plant used for dyes). Initial interviews were 831, and around 18% were eliminated, resulting in a total of 680 valid interviews. The resulting information, useful for the purposes of this work, was recorded for analysis.

### Data analysis

Once the dye plant catalog has been built, the percentages of the different parts of the plant used to extract the pigments, in relation to families, have been determined. Hence, the various anatomical parts of plant have been grouped into broad categories: leaves, roots, stem, flowers, fruits, and other. In addition, the percentages of the colors obtained from the different families have also been estimated. Graphical representations were produced for both data analyses. The same percentages were also analyzed for algae, fungi, and lichens. The data from the floristic survey of the species cited in the literature have been compared with the specific richness reported in the flora of dyeing plants. In this way, it was possible to verify the percentage of the flora actually represented and present in the Mediterranean territories of Southern Italy, after about two centuries. Data from semi-structured interviews have been recorded and compared with the flora of dyeing plants; informant consensus has been obtained with a simple frequency count of the single species cited in the total of citation emerged in the interviews [43]. In this way, it was possible to obtain an estimate of the percentage of TBK conserved over two centuries in the investigated territories, and on the other hand, the percentage of that knowledge that was lost. From the analysis of the answers of the interviewed, the relations between the species and the regional contexts were investigated.

## Results and discussion

### Flora of dyeing plants and catalog of dye plants

The flora of the dyeing plants produced (Supplementary File 1) shows 440 species of plants whose presence in Italy is verified in the bibliography [28, 41] and of which is made explicit dye use, except for 18 species. The catalog of dye plants (Supplementary Table 1) well reflects the flora in the contents, with the substantial difference in the order of the floristic list. The catalog is in fact ordered according to the APG IV criteria [40]. Data for a total of 72 entries have been recorded in the mushroom and lichen catalog (Supplementary Table 2), of which there is a dyeing use, except for 2 species [12, 22, 24]. For the algae, a total of 12 entries have been recorded (Supplementary Table 3), for which there is a dyeing use [12]. A wider and more complete database is in an embryonic phase, and the data currently available are those reported in the supplementary materials (Supplementary File 1; Supplementary Table 1, 2, 3). They consist of the floristic list and the tables with the uses and references. In the future, the database under development will be available online for the scientific community; it can be expanded and completed by experts in the field freely.

### Ethnobotanical prospection

The verification of the current presence on the territory of the dyeing species indicated in the literature [1, 2, 6, 7, 9, 11–18, 21–24, 30–39] enables a comparison to be made between the oldest floras [12–14] and the actual finding of the presence of the species in the wild, in the investigated territories. From the on-field survey operations, 283 plant species have been verified. These represent 64.31% of the species reported in the flora of the dyed plants produced (Supplementary Materials 1). The voices present in the flora and not found in the field could indicate a decrease in the species present in the South Italian territory which occurred over the last 200 years. The causes of this decrease could be attributed both to the modifications that took place in the textile sector, which would have caused a change in the cultivation and spread of some species, and to environmental changes that occurred over the last few centuries. In analyzing the results of valid interviews, the frequency in numbers of citations of each dye species, recorded by the interviewees, was recorded. The data relating to the species mentioned and the simple and percentage frequencies of citations have been reported in Table 1. Respondents recalled a total of 38 species; those with more quotations were *Isatis tinctoria* L. and *Juglans regia* L., with 655 and 523 citations respectively. This means that 96% of those interviewed knew and remembered the use of the *I. tinctoria*, and about 77% of them knew and remembered the *J. regia* for the extraction of natural dyes. This data confirms the large use and the importance given to *I. tinctoria* during the Bourbon kingdom [8], probably the reminiscence of this usage was conserved through the centuries, also after the end of the Bourbon dynasty. Analyzing the data from the regional point of view, as expected, it is observed that there are some species that are actually remembered in all the regions of Southern Italy and Major Islands, such as *I. tinctoria* and *J. regia* L., while others are totally missing in some regions but are widely or exclusively mentioned in others as shown in Fig. 5. Looking at the histograms in Fig. 5, we can see that from our field research, there was some exclusive usage throughout the investigated territories. The use of *Genista tinctoria* L. and *Chrozophora tinctoria* (L.) A.Juss were exclusive for Calabria region; the usages of *Quercus suber* L., *Rhamnus alaternus* L., *Phytolacca americana* L., *Phillyrea latifolia* L., *Populus nigra* L., *Ferula communis* L., and *Euonymus europaeus* L. were exclusive for Sardinia; the usage of *Curcuma longa* L. resulted exclusive for Sicily; the usage of *Fraxinus ornus* L. was exclusive for Molise region; and the usages of *Rumex acutus* L., *Rumex acetosa* L., and *Mentha aquatica* L. were exclusive for Basilicata region. The interviews aimed to confirm the uses of some dyeing plants found in the literature [1, 2, 6, 7, 9, 11–18, 21–24, 30–39] and allowed to determine the percentage of TBK eroded, in these territories, over the last two centuries through the comparison between the results of the interviews and the information collected in the flora. The number of species recalled by the total of the sample represents only about 9% of the dyeing flora reported in this work and which contains the memories of two hundred years knowledge of dyeing plants. Consequently, at the present state of things, it is clear that there has been a strong erosion of TBK amounting to about 91% of the knowledge concerning vegetable dyes.

**Table 1:** The data relating to species mentioned in the semi-structured interviews, the number of citations, and the percentage of citations for each species on the total of citations

Species	Citations	Citation%
*Alkanna tinctoria* Tausch subsp. *tinctoria*	196	29
*Alnus cordata* (Loisel.) Duby	146	21
*Alnus glutinosa* (L.) Gaertn.	102	15
*Anagyris foetida* L.	101	15
*Calendula arvensis* L.	118	17
*Capsicum annuum* L.	165	24
*Castanea sativa* Mill.	290	43
*Chrozophora tinctoria* (L.) A.Juss.	102	15
*Curcuma longa* L.	74	11
*Cynara scolymus* L.	223	33
*Daphne gnidium* L.	440	65
*Euonymus europaeus* L.	85	13
*Ferula communis* L.	132	19
*Fraxinus ornus* L.	49	7
*Genista tinctoria* L.	193	28
*Hedera helix* L.	124	18
*Isatis tinctoria* L.	655	96
*Juglans regia* L.	523	77
*Malva arborea* (L.) Webb & Berthel.	143	21
*Matricaria chamomilla* L.	95	14
*Mentha aquatica* L.	41	6
*Papaver rhoeas* L.	218	32
*Phillyrea latifolia* L.	101	15
*Phytolacca americana* L.	108	16
*Populus nigra* L.	126	19
*Punica granatum* L.	414	61
*Quercus cerris* L.	392	58
*Quercus suber* L.	132	19
*Reseda luteola* L.	237	35
*Rhamnus alaternus* L.	123	18
*Rhus coriaria* L.	266	39
*Rubia peregrina* L.	201	30
*Rubia tinctorum* L.	460	68
*Rumex acetosa* L.	34	5
*Rumex acutus* L.	46	7
*Sambucus nigra* L.	406	60
*Solanum lycopersicum* L.	41	6
*Urtica dioica* L.	169	25

### Used parts of plants

**Fig. 1:**
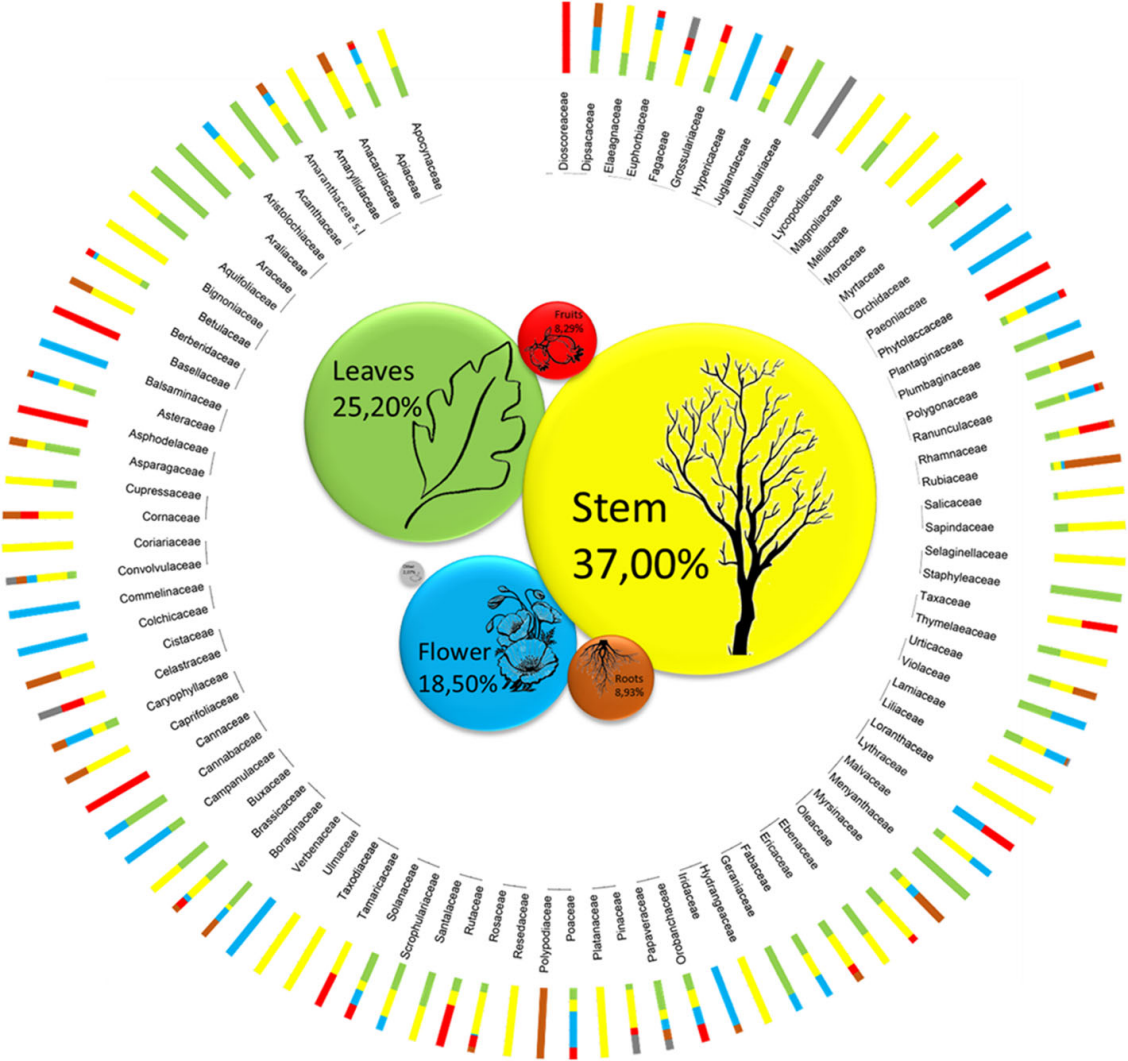
Differences between the percentages of the parts used in the various plant families reported in the flora of dye plants (Supplementary File 1). In the central section of the graph, there are the percentages of use of the anatomical parts in total

The percentages of the parts used to extract the pigments from the species registered in the catalogs have been calculated. For the plants, the data was calculated both in total and for families. A graph was produced that highlights the differences between the percentages of the parts used in the various plant families reported in the flora (Fig. 1). From the analysis of the data, it appears that the uses are divided into 37.00% stem, 25.20% leaves, 18.50% flowers, 8.93% roots, 8.29% fruits, and the remaining 2.08% for other (in the latter category, fall ashes and coals). The same operation performed on the plant catalog was carried out on the algae and fungus and lichen tables. Our results show that, for algae, 100% of the whole organism is used to extract pigments, while for fungi and lichens, the uses are divided into the whole plant 88.71% and juices for the remaining 11.29%.

### Colors extracted from plants

**Fig. 2:**
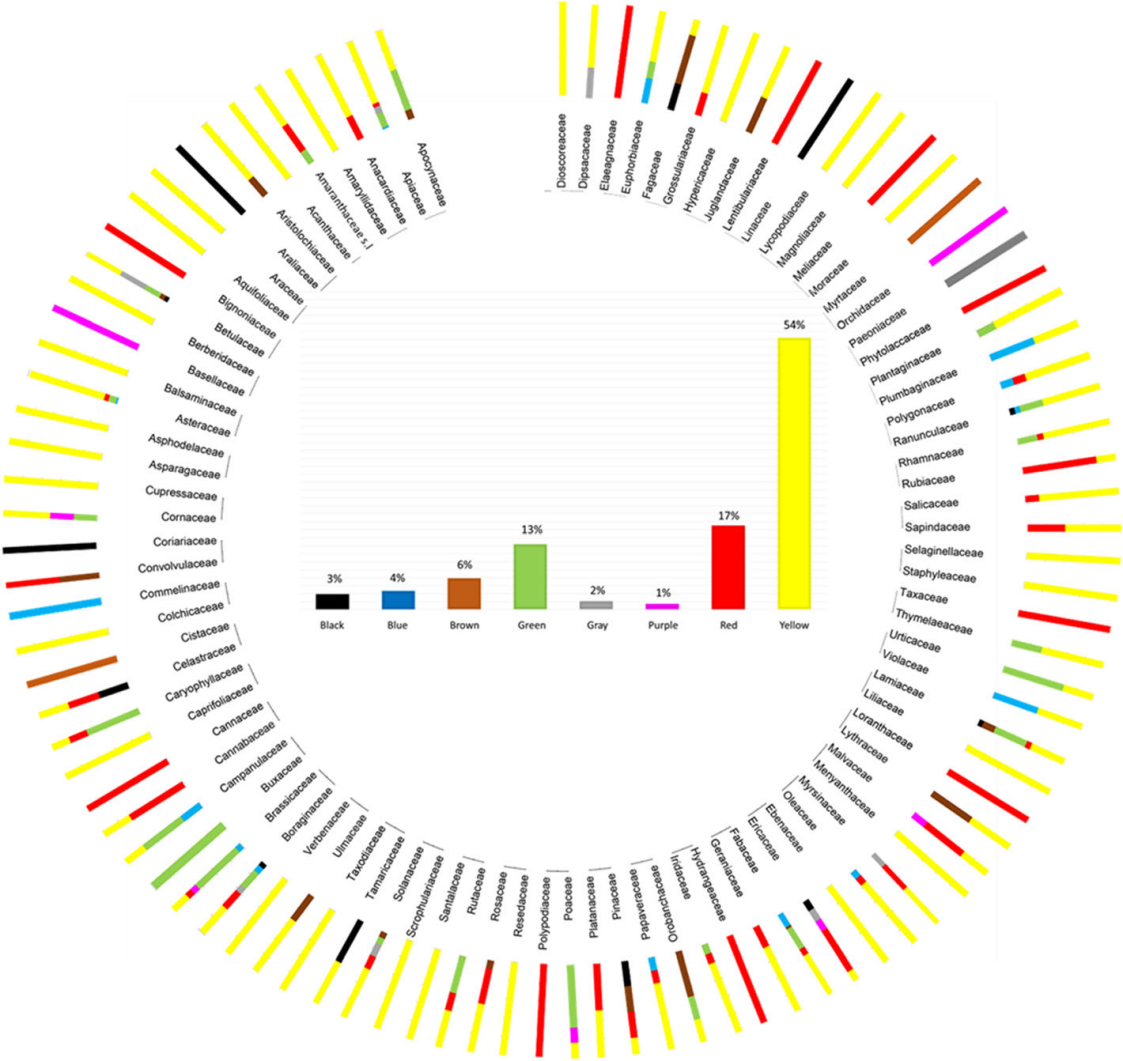
The different color breakdowns in plant families reported in the flora of dye plants (Supplementary File 1). At the center of the graph are the percentages of the extracted colors in total

**Fig. 3:**
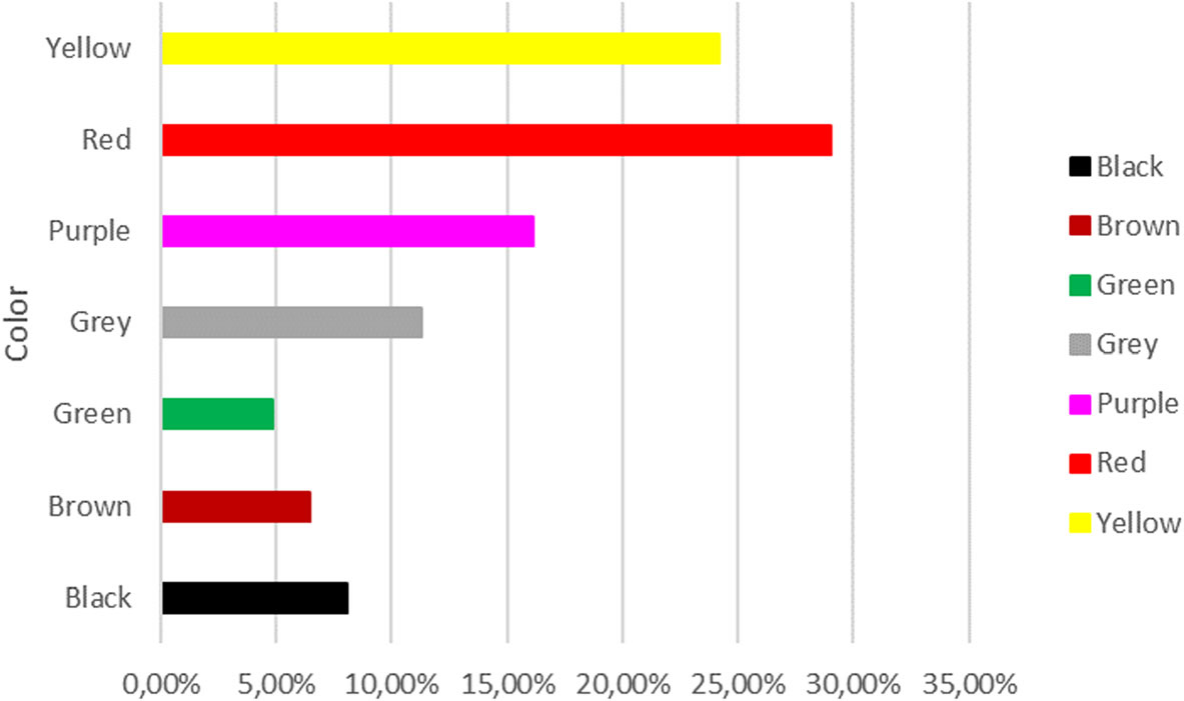
Total percentages relative to the colors taken from fungi and lichens reported in Supplementary Table 2.

**Fig. 4:**
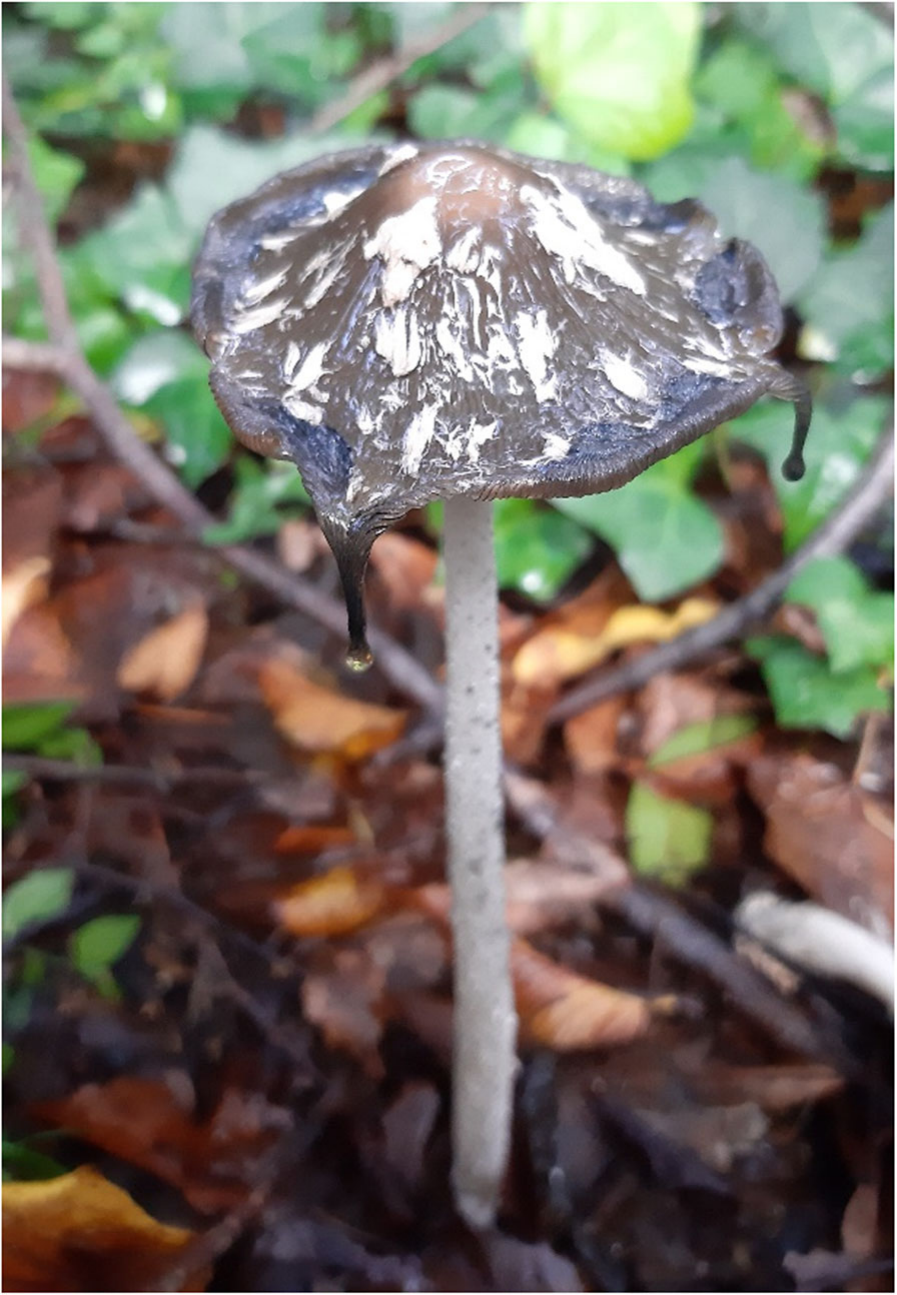
*Coprinus comatus* (O. F. Mull.) Pers., a mushroom used to extract black pigment. In the picture, the peculiarity of its black deliquescent spores is shown

For all the catalogs, the data related to the extracted colors have been analyzed. For the plant catalog, given its size, the percentages of colors drawn for families and overall were calculated. A graph has been created that highlights the different color breakdowns in families (Fig. 2). The total percentages of the extracted colors are 54% yellow, 17% red, 13% green, 6% brown, 4% blue, 3% black, gray 2%, and purple 1%. The dominance of yellow, extracted from plants, is not surprising. The range of colors that starts from yellow and turns towards red (the second most represented color) is in fact obtained mainly from the variety of tannins present in plants in large quantities [30]. The percentages of use of anatomical parts in plants confirm this data. Most uses concern the stems and leaves, rich in tannins which may help regulate the growth of these tissues [30]. Green color is extracted mainly from leaves and fresh tissues, rich in chlorophylls. Other colors, like purple or blue, are extracted from plants having molecules like indigotin [30]. This molecule is present only in some species of plants, and therefore, the blue color appears to be poorly represented in this analysis. However, it is known that the cultivation of plants for blue dyeing has been very important in history, up to the present day [8]. The percentages relative to the colors taken from fungi and lichens are shown in Fig. 3. Unlike plants, there is a greater presence of red and a net decrease of green compared to other colors. Specifically, the colors in mushrooms and lichens are divided into red 29.03%, yellow 24.19%, purple 16.13%, gray 11.29%, black 8.06%, brown 6.45%, and green 4.84%. The use of fungi and lichens is also reported in some works of literature [6, 9, 22, 24, 30, 44–46]. The algae show greater uniformity in the colors deductible and usable by them: violet 75% and green for the remaining 25%. Due to the lack of information on algae, fungi, and lichens, the data obtained from catalog analyses, on colors and parts used, require more accurate experimental checks to determine a possible dyeing use of the species mentioned by Briganti [12]. Of great importance seems to be the species *Coprinus comatus* (O. F. Müll.) Pers. used to extract the black color. This mushroom, edible before the spore production, was discussed in some notes by Briganti himself [12] for the peculiarities of his black deliquescent spores (Fig. 4).

## Conclusions

**Fig. 5:**
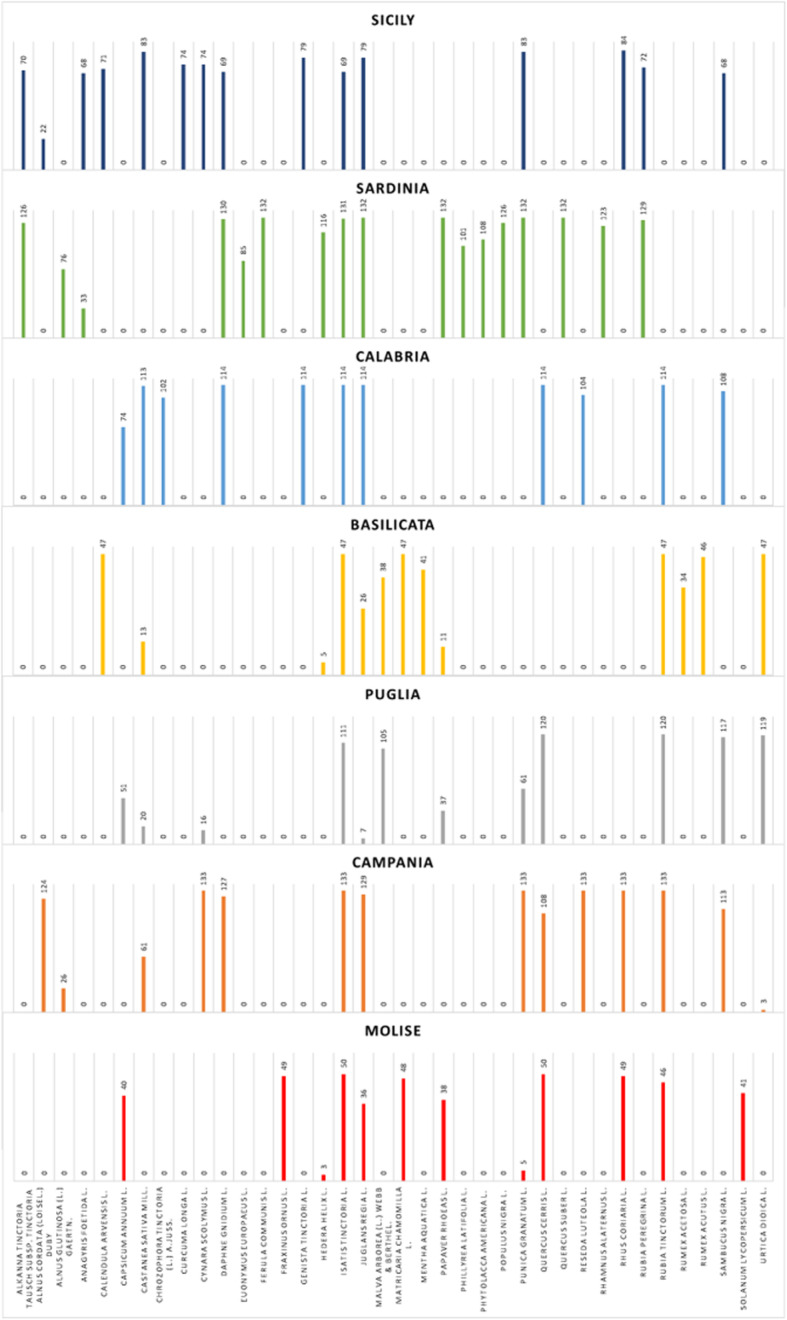
Uses of dye plant species in the region of Southern Italy. Data obtained from the semi-structured interviews

Today, synthetic chemicals used in dyes for the textile industry are known to be a principal source of environmental pollution (Fig. 5) [1]. Synthetic dye substances also have several carcinogenic properties and cause allergies in humans [1, 38]. It is estimated that, during industrial processing, the use of chemical substances can reach 1 kg per 1 kg of processed textile product [1]. At the European level, some directives have placed restrictions on the production and use and placing on the market of a growing number of synthetic dyes and auxiliary chemical compounds, indirectly favoring the application of natural dyes in textile finishing processes [1, 38]. Natural dyes cause less environmental pollution and health problems [47]. The development of a production chain linked to natural textiles and artistic craftsmanship could also contribute to the development of the agricultural sector with a view to multi-functionality of agricultural production systems, considering the articulated rural system of many Italian regions, and the propensity to innovation of the agricultural companies that operate there [1]. The flora of dyeing plants, realized in this work, is a concentration of ethnobotanical knowledge of dimensions never recorded so far, in the field of dyeing plants. The catalogs produced and the flora of the dyeing plants are at the base of the potential recovery of an eco-sustainable culture and economy linked to rural areas and that could change the living conditions of the people who live and live in non-intensive agriculture contexts. The species reported in the catalog allow the extraction of many pigments and colors; the great biological variety reported would allow an ad hoc agro-territorial planning calibrated on the needs and characteristics of the various regional contexts. The presence of so many different species would allow a diversification of hypothetical cultivations for the development of the marginal territories of Southern Italy, and not only. The representativeness analysis of the presence of the recorded species, and their uses, in the Mediterranean Basin area, and in the South of Italy, allows the possibility of recovery and reintegration of crops no longer registered. The erosion of ethnobotanical knowledge linked to vegetable dyes is an indication of how much knowledge is now lost. With in-depth studies on individual species, linked both to the sustainability of production processes and their remunerability, it will be possible, in the future, to create new development prospects in areas with different agro-cultural vocation.

## Supplementary information

**Additional file 1: Supplementary File 1.** A wider and more complete database and the currently available data.

**Additional file 2: Supplementary Table 1.** Dye plants catalog, sorted by APG IV criteria.

**Additional file 3: Supplementary Table 2.** Dye fungi and lichens catalog.

**Additional file 4: Supplementary Table 3.** Dye algae catalog.

## Data Availability

Not applicable
