# Supplementary material for: Ethnobotany of dye plants in Southern Italy, Mediterranean Basin: floristic catalog and two centuries of analysis of traditional botanical knowledge heritage
Source: J Ethnobiol Ethnomed. 2020 Jun 3;16:31. doi: 10.1186/s13002-020-00384-2 (PMC7268309; doi:10.1186/s13002-020-00384-2)
Supplement: Supplementary file 1 — Additional file 1: Supplementary File 1. A wider and more complete database and the currently available data. [file 13002_2020_384_MOESM1_ESM.pdf]

Flora of Dye Plants

Information reported on this Flora are:
Species name (Roskov et al., 2019), Family name (Roskov et al., 2019), habitat and Italian locality where the species can be found or where they have been reported (Pignatti et al., 2019), altitudinal range and chorology (Pignatti et al., 2019), chromosome numbers (if known) (Pignatti et al., 2019; Tropicos, 2020), pigment color and pigment sources (Briganti, 1842), Herbarium voucher (Index Herbariorum acronym, and voucher number).
Species are listed in alphabetical order. The frequency of the species on the Italian territories are reported with: RR (very rare), R (rare), C (common), CC (very common) (Pignatti et al., 2019).

Abies alba Mill.
Pinaceae
Mountain woods in the beech belt. Alps and Apennines up to Aspromonte: C, but with large gaps; elsewhere frequently cultivated (400-1800 m).
2n=24
Brown: young branches
Black: soot
H.PAOL – 2207

Acanthus mollis L.
Acanthaceae
Arid fallows, bushes. Liguria, Peninsula (especially along the coasts), Sicily, Sardinia, and smaller islands: C. (0-700 m). Steno-Mediterranean-West.
2n=80
Yellow: grass
PAL – 77570

Acer campestre L.
Sapindaceae
Mesophilous woods, on rich soil; also, commonly cultivated in hedges and vineyards. In the whole territory, however, it is lacking in the high mountains and in the most arid areas of the Mediterranean: C; in Sardinia, it is lacking in the spontaneous state. (0-800, in Sicily up to 1600 m). European-Caucasian (Subpontic).
2n=26
Red: fresh wood, bark
PAL – 69273

Acer opalus subsp. *obtusatum* (Waldst. & Kit. ex Willd.) Gams
Sapindaceae
Broad-leaved woods, especially *Quercus cerris*' woods. Peninsula from Southern Lazio, Molise, and Gargano to Calabria: R. (500-1300 m). Endemic.
2n = unknown
Red: bark
PI - 010548

Acer pseudoplatanus L.
Sapindaceae
Mountain woods, especially beech woods. Alps, northern and central Apennines (including minor reliefs): C; Padania, southern Apennines, and northern Sicily: R. (500-1500, rarely 0-1900 m). European-Caucasian.
2n=52

Red: bark
PAL – 70480

Actaea spicata L.
Ranunculaceae
Mountain woods, especially beech woods. Alps and Apennines up to Sila: C. (400-1500, max 1900 m). Eurasian tempered.
2n=16
Black: berries' juice
PAL-GR – 100306_GR

Aesculus hippocastanum L.
Sapindaceae
Commonly cultivated for ornamentation. In the whole territory area, sub-spontaneous, especially in northern and central Italy (50-1300 m). Balkan.
2n=40
Yellow: bark, young branches, leaves
NYBG - 2497668

Aesculus pavia L.
Sapindaceae
Exotic, native species in the USA from Illinois to Missouri.
Yellow: bark, young branches, leaves
NYBG - 1269329

Agrimonia eupatoria L.
Rosaceae
Woodland margins, meadows, and pastures, fallows. (0-1000, rarely 1500 m). Sub-Cosmopolitan.
2n=28
Yellow: leaves, stem
PAL – 76608

Alcea rosea L.
Malvaceae
Cultivated for ornament. Reported as sub-spontaneous in northern Italy, Tuscany, Lazio, Abruzzo, Basilicata, Sicily, and Sardinia, but in general due to confusion with *A. setosa* (0-800 m). Unknown origin.
2n=26
Red: flowers
PAL – 85348

Alkanna tinctoria Tausch subsp. *tinctoria*
Boraginaceae
Arid fallows, sandy, or rocky. Liguria, Molise, Puglia, Basilicata, Calabria, Sicily and Sardinia: R. (0-600 m). Steno-Mediterranean.
2n=30
Red: roots
Yellow: leaves
PAL – 72291

Allium cepa L.
Amaryllidaceae

Bulbous herbaceous plant, cultivated since ancient times. Random archaeophyte in different regions of the Italian Peninsula. W-Asiatic
2n=16
Red: cataphyll
NYBG - 1504442

Alnus cordata (Loisel.) Duby
Betulaceae
Gorges and mountain woods (oak, chestnut, beech). Southern Apennines from the Neapolitan to the Sila (especially on the Tyrrhenian side): C; also on the Majella in Pretoro, Elba (spontaneous?); doubt in Sardinia; cultivated in the Prealps in the province of Pordenone, Belluno, on the Garda and in the northern Apennines (Campigna, Futa). (0-1500 m). Endemic.
2n=unknown
Red: inner bark
Yellow: bark, cones
Green: catkins
PAL – 107986

Alnus glutinosa (L.) Gaertn.
Betulaceae
Woods and bushes on the banks of streams and on peaty, asphyxial soils. In the whole territory, in the North: C; in the South: R. (0-800, rarely 1200 m). Paleo-tempered.
2n=28
Red: bark
Yellow: leaves, bark, young branches
Brown: bark
Black: cones
PAL – 68228

Amelanchier ovalis Medik.
Rosaceae
Sparse woods, on slopes and also on rocky places with limestone soils. Whole territory: R. (0-2200 m). Mediterranean
2n=34, 68
PAL – 68226

Amorpha fruticosa L.
Fabaceae
Pebbly shores and river beds. Cultivated and feral in northern, central, and southern Italy, and in Sardinia: C; of American origin, it was introduced in 1850, first in the North, and now expanding throughout the territory; missing in Sicily. (0-600 m). N-American.
2n=40
Blue: tender shoots, leaves
PAL – 97055

Anagyris foetida L.
Fabaceae
Spots and cliffs (calcareous). Southern Italy (north to Gargano and Naples), Sicily and Sardinia: C; northwards it radiates on the Adriatic side up to the Marche in Fabriano, on the Tyrrhenian side up to the Alban Hills, Tuscany in Follonica and Magliano, Liguria in Varigotti. (0-1100 m). South Mediterranean.
2n=18

Yellow: leaves
PAL – 26

Anchusa ochroleuca M. Bieb.
Boraginaceae
Ruins, ferrivia embankments, torrential beds. Spotted from 1893 to 1930 in Trieste (S. Elia Draga Station) and from 1893 to 1914 in the Frigido stream bed in the province of Massa, but in both cases, it was no longer found and probably to be eliminated from the Italian Flora. (0-300 m). E-European.
2n=16
Red: roots
Green: leaves
Blue: flowers
VHLVB - 10 0841337

Anemone nemorosa L.
Ranunculaceae
Broadleaf woods. Alps: C; Padania. Spontaneous everywhere, but mostly disappeared due to the crops; Peninsula, above all on hills, in the South up to Pollino: C. (0-1500 m).
2n= (16, 24), 30, (45)
Yellow: leaves
PAL-GR – 100303_GR

Angelica archangelica L.
Apiaceae
Allochthonous casual in the gardens where it is cultivated. Alps in Lombardy: R. Not confirmed recently in Emilia, Tuscany, and Abruzzo (500-1400 m). E-European and Siberian, archaeophyte.
2n=22
Yellow: leaves
NYBG - 3365663

Angelica sylvestris L.
Apiaceae
Wet woods and ravines. Throughout the national territory (dubious in Puglia and Sardinia): C. (0-1600 m). Euro-Siberian.
2n=22
Yellow: roots, leaves, stem
PAL – 21266

Anthemis cotula L.
Asteraceae
Uncultivated, ruins, cereal fields. In the whole territory, however, in the Po Valley it lacks or ephemeral adventitia and generally on the Alps in the valley floor or dry sunny slopes: C. (0-1300 m). Euri-Mediterranean.
2n=18
Yellow: leaves, stem
PAL – 28876

Anthriscus cerefolium (L.) Hoffm.
Apiaceae

Allochthonous casual or sometimes naturalized near the cultivated and uncultivated. Remnant of ancient crops in continental Italy (with large gaps, often missing) and Sardinia. (0-1000 m). Eurasian (archaeophyte).
2n=18
Yellow: leaves, green stem
NYBG - 3366383

Anthriscus sylvestris (L.) Hoffm.
Apiaceae
Ruined orchards and woodland environments. In much of continental Italy, but often confused with *A. nemorosa*. (0-1500, rarely 2200 m). Paleo-tempered.
2n=16
Yellow: umbels
Green: grass
PAL – 97825

Anthyllis vulneraria L.
Fabaceae
Northern European plant that does not exist in Italy in the spontaneous state. N-European.
2n=12
Yellow: grass
Blue: flower
PAL – 83445

Antirrhinum majus L.
Plantaginaceae
Cliffs, rocks, rubble, and walls. Cultivated by ornament and sub-spontaneous throughout the territory: R. (0-800 m). W-Mediterranean.
2n= 16, 24
Yellow: stem, flower
PAL – 43139

Apera spica-venti (L.) P.Beauv.
Poaceae
Infesting wheat crops on siliceous sandy soil. Padania: C; Basilicata and Calabria: R: reported once in Sardinia. (0-1000 m). Euro-Siberian.
2n=14
Green: spike
PAL – 96903

Arctium lappa L.
Asteraceae
Uncultivated, ruins, edges of roads, hedges. Widespread throughout the territory, but often confused with *A. nemorosum*. (0-1100 m). Eurasian.
2n=36
Yellow: roots, leaves
PAL – 96903

Arctostaphylos uva-ursi (L.) Spreng. subsp. *uva-ursi*
Ericaceae

Mountain and subalpine pine forests, rhododendron bushes. Alps, from the Giulie to the Maritime and the Abruzzese Apennines: R; in the rest of the Apennines from Liguria to Salerno: RR. (600-2500 m). Circum-Arctic-Alpine.
2n=52
Red: roots, whole plant
Grey: leaves
Black: leaves
PAL – 102655

Aristolochia clematitis L.
Aristolochiaceae
Woodland margins and uncultivated on damp soils, often sandy, rich in nitrates. Northern and central Italy, south to Ostia, Maiella and Giulianova: C; Puglia province of Bari and Sicily on the Nebrodi: R. (0-1400 m). Euri-Mediterranean.
2n=14
Yellow: leaves, stem, flower
PAL – 89661

Arnica montana L.
Asteraceae
Pastures, rhododendron moors, dry meadows, always on acid soil. Alps from Gorizia to Cuneese, Piedmontese and Pavese Apennines: C; on the Piacentino Apennines and Parmigiano: R. (500-2200, in Friuli up to the plain, in the Lombard Alps up to 2650 m). Central European orophyte.
2n=38
Yellow: leaves
PAL-GR – 54405_GR

Artemisia absinthium L.
Asteraceae
Arid uncultivated, hedges, walls. Northern Italy and the Peninsula as far as Basilicata, Tremiti, in the arid hilly belt: C, missing in the Padania almost everywhere. (0-1100 m). Sub-cosmopolitan.
2n=18
Yellow: pulverized dry stem
PAL – 107797

Artemisia dracunculus L.
Asteraceae
Cultivated as a condiment and sub-spontaneous in the vegetable gardens. Sarmantico-Siberian.
2n=18 (36, 54, 87-90)
Yellow: woody stem
NYBG - 1893763

Artemisia vulgaris L.
Asteraceae
Uncultivated, rubble, generally synanthropic. In the whole territory; Padania: CC, rest of northern Italy: C, elsewhere R. (0-1000, in Livigno up to 1800 m). Circumboreal.
2n=16
Yellow: stem
PAL – 9364

Arum maculatum L.
Araceae

Beech woods, clearings, coppices. Northern Italy, Peninsula, (on the reliefs): R; the indications for Sardinia are probably due to confusion. (0-1600 m). Central European.
2n=56
Yellow: leaves
PAL – 79278

Asarum europaeum L.
Aristolochiaceae
Broad-leaved woods on neutral and well-humidified soil. Alps and Northern Apennines: C; Lazio, Abruzzo to the Gran Sasso, Campania and Basilicata to the Lake of Sirino, northern Calabria: R. (0-1500 m) Euro-Siberian.
2n=26
Brown: whole plant
PAL – 89613

Asclepias syriaca L.
Apocynaceae
Cultivated by fiber and naturalized in damp woods and hedges. Riparian forests of the Ferrara area: C; also in the Trieste, Veneto, Trentino, and provinces of Brescia, Pavia, Parma and Piedmont. (0-200 m). N-American.
2n=22
Green: leaves, stem
NYBG - 2281009

Asperula arvensis L.
Rubiaceae
Fields, dry pastures, uncultivated. In the whole territory; Liguria Peninsula (north to the Via Emilia) and Islands: C; Padania and southern slopes of the Alps: R. (0-1500 m). Euri-Mediterranean.
2n=22
Red: roots
PAL – 59262

Asperula cynanchica L.
Rubiaceae
Dry meadows and pastures, stony (preferably calcareous). In the whole territory: C; in the alluvial plains, it is missing or R. (0-1000, rarely 2000 m). Euri-Mediterranean.
2n= 20, 40
Red: roots
PAL – 60597

Asperula laevigata L.
Rubiaceae
Thermophilic broadleaf woods. Liguria, Peninsula, Sicily and Sardinia: R. (0-1500 m). W- and Central-Mediterranean.
2n=22
Red: roots
PAL – 62491

Asperula purpurea (L.) Ehrend.
Rubiaceae

Stones, cliffs, calaches and pastures. Eastern Alps and pre-Alpine reliefs from the Trieste Karst to the Insubria: C; in the western Alps, generally R; Cuneo, Liguria, Central Italy and Campania up to Alburno: C. (0-1400 m). SE-European orophyte.
2n=22
Red: roots
H.PAOL - 2614

Asperula taurina L.
Rubiaceae
Mesophilous broad-leaved woods (oak, linden, beech). Apennine system from Liguria to Sila: C, advances northwards to via Emilia, Langhe, southern slopes of the Alps from Gorizia and Friuli to Piedmont: R. (100-1700 m). S-European orophyte. and SW-Asian.
2n=22
Red: roots
NYBG – 3358415

Asperula tinctoria L.
Rubiaceae
Woods, deciduous forests. Lazio on the Alban Hills: R; Also, reported in Trieste and Venice (perhaps disappeared), Moldovì and Parmigiano. (0-600 m). European.
2n= 22, 44
Red: roots
NYBG - 3358418

Asphodeline lutea (L.) Rchb.
Asphodelaceae
Arid and stony slopes. Southern Italy and Sicily: C; Tuscany (Uccellina, Argentario), Marche, Lazio (Circeo, Monte Autore, Tivoli and Monte Guadagnolo), Abruzzo, Sardinia: R. Also in Istria, but outside the Italian territory. (0-1700 m). E-Mediterranean.
2n=28
Yellow: green capsule
PAL – 67933

Asphodelus aestivus Brot.
Asphodelaceae
Uncultivated dry, pastures, garrigues (often on acid soil). Liguria, Peninsula, above all on the western slope in Tuscany, Lazio and in all Southern Italy, in land up to Norcia; on the Adriatic coast, perhaps only in Puglia, in the nineteenth century sighted in Pesaro, but later disappeared, Sicily, Sardinia, and the smaller islands: C. (0-1200 m). Steno-Mediterranean.
2n=28
Yellow: green capsule
PAL-GR – 51869_GR

Aster amellus L.
Asteraceae
Bushes, coppices, at the edge of the woods (calcareous). Northern Italy in the hills and low mountains (no longer found in Liguria): R; Central Italy: Marche (at the Furlo, near Sarnano and Senigallia); Umbria, no longer found in Tuscany. (0-800 m). Central European-S-Siberian (Subpontic).
2n=18
Yellow: stem, dry flower
PAL – 27222

Atriplex hortensis L.
Chenopodiaceae
Uncultivated, ruderal environments. Naturalized in Emilia Romagna and Abruzzo; casual In Northern Italy, Marche, Umbria, Basilicata, and Sicily; no longer found in Lazio and Tuscany. (0-600 m). Temperate Asia. (Archaeophyte).
2n=18
Yellow: leaves, stem
PAL – 90384

Atropa belladonna L.
Solanaceae
Humid clearings, coppices, brightened deciduous forests (especially beech woods, rarely oaks). In the whole territory: R; it is almost everywhere in Padania (perhaps disappeared due to land reclamation and crops) and in the Mediterranean area. (0-1400, in Sicily up to 1800 m). Mediterranean mountain.
2n=72
Green: berries
PAL – 68490

Ballota nigra L.
Lamiaceae
Ruins, uncultivated, hedges (nitrophilous). In the whole territory: C. (0-1300 m). Euri-Mediterranean. (perhaps archaeophyte).
2n= 20, 22
Green: stem, flower
PAL – 15642

Barbarea vulgaris (L.) W.T. Aiton
Brassicaceae
Wet mud, uncultivated, banks of streams. In the whole territory excluded Puglia and Calabria (perhaps only not observed): R. (0-1600 m). Eurosiberian become Cosmopolitan.
2n=16
Green: leaves
PAL – 76659

Basella alba L.
Basellaceae
Rarely cultivated. SE-Asia, Africa, America.
2n=48
Purple: berries
NYBG - 919326

Berberis vulgaris L.
Berberidaceae
Arid slopes, pine forests, degraded sub-Mediterranean forests. Alps, from the Giulie to the Marittime and Carso Triestino: C; Padania and Peninsula up to Macerata and Spoleto: R, also in Abruzzo (Gran Sasso, Sirente, Pizzoli, Marsica), Lazio in Civita Castellana and the province of Rome, Cuma, Gaeta, Avellino, Vulture, A. of Latronico, Muro Lucano, Pollino. (100-2000 m). Eurasian.
2n=28
Yellow: roots, bark, wood
PAL – 99159

Beta vulgaris L. subsp. *vulgaris*
Amaranthaceae
Spontaneous on the coasts and commonly cultivated. (0-600 m). Euri-Mediterranean.
2n=18
Red: roots
PAL – 108211

Betonica officinalis L.
Lamiaceae
Dry meadows, pastures, nardeti, molinieti. In the whole territory excluding the Padania and the Islands: C. (0-1800 m). European-Caucasian.
2n=16
Yellow: leaves, flowery stem
PAL – 37060

Betula pubescens Ehrh. var. *pubescens*
Betulaceae
Marsh woods, peat bogs: in the Italian Alps, it is a very rare species, the most frequent are the hybrids *B. pendula x pubescens*. Alps, from the Carso to the Val Susa and Val Pellice, Val Chisone and Val Pesio, the Parmesan Apennines: R. (600-2150 m). Eurosiberian.
2n=56
Red: branches, wood, bark
Yellow: leaves
PAL – 88760

Bidens tripartita L.
Asteraceae
Ditches, marshes, muddy lands, radurae of the alveale woods. Friuli, Trentino, South Tyrol, Lombardy, Piedmont, Val d'Aosta, Emilia Romagna, Marche, Umbria, Abruzzo and Sicily: R. (0-800 m). Eurasian.
2n=48
Yellow: stem, leaves
PAL – 108433

Bistorta officinalis Raf. subsp. *officinalis*
Polygonaceae
Fertilized meadows in the subalpine belt and other stations with soil rich in nitrates. Alps and Apennines up to Calabria: C. (900-2000 m). Circumboreal.
2n=24
Red: roots
NYBG - 3063394

Borago officinalis L.
Boraginaceae
Fields, uncultivated, ruins, roadsides. In the whole territory: C. (0-800, rarely 1400 m). Euri-Mediterranean.
2n=16
Yellow: leaves, flowery stem
PAL – 87644

Bromus secalinus L.

Poaceae
Infesting cereal crops, especially wheat and rye. Northern Italy, Peninsula up to Basilicata: C. (0-1200 m). Eurosiberian.
2n=28
Green: grass, spike
PAL – 32461

Broussonetia papyrifera (L.) Vent.
Moraceae
Cultivated and naturalized in ruderal environments. In the whole territory: C. (0-600 m). East Asia.
2n=26
Yellow: branches
NYBG - 2512306

Buglossoides arvensis (L.) I.M.Johnst.
Boraginaceae
Cultivated or uncultivated grassland areas, pastures. In the whole territory: C. (0-1800 m). Euri-Mediterranean.
2n=28
Red: roots
PAL – 68453

Buxus sempervirens L.
Buxaceae
Broad-leaved thermophilous woods, cliffs, stony ground. Spontaneous only in the western Alps and northern and central Apennines up to the Alban Hills and Abruzzo and Campania: R; isolated stations in Carnia; commonly cultivated in gardens throughout the territory and often wild. (0-800 m). Sub-Mediterranean-Subatlantic.
2n=28
Green: leaves, branches
PAL-GR – 65582_GR

Calendula arvensis L.
Asteraceae
Uncultivated, edges of streets, fields, and vineyards. In the whole territory (excluding Val d'Aosta): C; anciently reported in Trentino and southern Tyrol (0-600 m). Euri-Mediterranean.
2n= 36, 44
Yellow: petals, flowery grass
PAL – 83716

Calendula officinalis L.
Asteraceae
Cultivated for ornamentation and commonly feral. Northern Italy (excluding Trentino, southern Tyrol, Lombardy, and Val d'Aosta), central and southern Italy (not reported in Calabria): R. (0-600 m). W-Mediterranean.
2n=28, 32
Yellow: petals, flowery grass
PAL – 83724

Callistephus chinensis (L.) Nees
Asteraceae

Gardens, shores, rubble. Random in northern Italy (citations for Friuli, Lombardy, Piedmont, Val d'Aosta, Emilia Romagna): R. (0-500 m). Mongolia, China, E-Asia.
2n= 18, 36
Yellow: leaves, stem
NYBG – 2447903

Calluna vulgaris (L.) Hull
Ericaceae
Moors, lean pastures, bushes, coniferous forests, always on highly acidified soils. Alps, Northern Apennines: C; Padania and central Apennines up to the Marche, Umbria and northern Lazio in Monte Rufeno: R. an isolated station in Calabria, in Serra S. Bruno (0-2000, max 2750 m). Euro-American Circumboreal (Anfi-Atlantic).
2n=16
Red: branches
Yellow: whole plant
PAL – 89217

Caltha palustris L.
Ranunculaceae
Wet meadows and banks of waterways. Northern Italy: C (in Padania, however, sometimes absent); Tuscan-Emilian Apennines, Umbria, Pontine Marshes, Fondi Abruzzo, Basilicata, Sila; Sardinia on Mount Tuttavista. (0-2000, max 2530 m). Circumboreal.
2n=32, 53-62
Yellow: flower, grass
PAL-GR – 100368_GR

Campanula pyramidalis L.
Campanulaceae
Cliffs and rocks (calcareous). Carso Triestino and Gorizia, eastern Friuli up to Gemona: R; naturalized on the walls in Veneto and Bresciano (0-600 m). Illyrian.
2n=32
Yellow: flowery stem
PAL – 39970

Campanula rotundifolia L.
Campanulaceae
Meadows, stony slopes, walls, cliffs (acid substrates, rarely on limestone). Alps from Gorizia to Susa Valley: C; Tuscan Apennines: R. (100-2000 m). N- and Central-European.
2n= 34, 68, 102
Green: leaves, flowery stem
Blue: flower
PAL – 40209

Campsis radicans (L.) Bureau
Bignoniaceae
Cultivated by ornament and sub-spontaneous on old walls. Lombardy, Langhe, Colli Emiliani, Lazio: R. (0-600 m). N-American.
Red: branches
NYBG - 2456451

Canna indica L.
Cannaceae

Cultivated widely for ornamentation and sub-spontaneous in various coastal regions (Liguria, Tuscany, Lazio etc.); already reported in Sicily in Syracuse. Pantropic of Neotropic origin.
2n=18
Red: seeds
PAL – 86651

Cannabis sativa L.
Cannabaceae
Cultivated mainly in Romagna and in the Neapolitan area for fiber, throughout the territory in the vegetable gardens for seed and rarely sighted (0-800 m). Central Asian.
2n= 20, 40, 80
Yellow: grass
PAL – 86004

Capsella bursa-pastoris (L.) Medik.
Brassicaceae
In the uncultivated and cultivated. In the whole territory, northern Italy: CC; Peninsula and Islands especially on the reliefs. (0-1800, max 2600 m). Cosmopolitan (Sinantropic).
2n=32
Yellow: plant, seeds, leaves
PAL – 5917

Capsicum annuum L.
Solanaceae
Cultivated for food in many varieties, throughout the territory, but does not tend to spontaneously become. S-American.
2n= 24, 36
Yellow: leaves, stem, fruit
PAL – 107838

Carpinus betulus L.
Betulaceae
Mesophilic woods. Together with *Quercus robur* in the plains, more rarely in the hills, but only on moist, rich, well humified ground: it formed climatogenic forests in Padania (today largely destroyed). Alps and Peninsula on the reliefs: C; it goes down to the sea in the Maremma and Agro Pontino; missing in the Islands. (0-1200 m). Central European-Caucasian.
2n=64
Yellow: bark
PAL – 88725

Carthamus lanatus L.
Asteraceae
Uncultivated arid, olive groves, vineyards, on clays. In the whole territory: C; progressively rarer in the North. (0-1300 m). Euri-Mediterranean.
2n=22
Yellow: flower
PAL – 10970

Carthamus tinctorius L.
Asteraceae
Uncultivated, along the streets. Random in Trieste, Veneto, Piedmont, and Umbria; ancient indications in Emilia Romagna and Tuscany. (0-600 m). Temperate Asia.

2n=24
Red: flower
Yellow: stamens
PAL – 10907

Castanea sativa Mill.
Fagaceae
Woods, generally on acid soils. In the whole territory, C on the reliefs (but R on limestone), absent in the alluvial plains; missing in Padania. (0-1200 m). SE-European.
2n=24
Brown: bark, wood, fruit rind
PAL – 69497

Catalpa bignonioides Walter
Bignoniaceae
Cultivated by ornament and sub-spontaneous in ruderal environments and along the streets. Colli Veneti, Adige Valley up to Bolzano, Lombardia: R. (0-600 m). N-American.
Red: branches
NYBG - 2584060

Celosia argentea L.
Amaranthaceae
Ruderal environments (cultivated for ornament, sometimes escaping from cultivation). Distribution to be defined, excluded from Piedmont and Tuscany. Asia Tropical.
2n=72
Red: flower
NYBG – 1374124

Celtis australis L.
Ulmaceae
Arid woods on limestone. In the whole territory: C, however, frequent only as cultivation and naturalization. (0-800 m). Euri-Mediterranean.
2n=40
Yellow: branches, bark
Brown: wood
PAL – 69687

Centaurea benedicta (L.) L.
Asteraceae
Fields at rest, uncultivated. Tuscany, Campania, Puglia and Sardinia; considered alien in Molise; dubious in Lazio and Abruzzo (perhaps as a remnant of ancient crops; medicinal plant). (0-800 m) W-Mediterranean.
2n=18
Yellow: leaves
VHLVB - 10 0635234

Centaurea cyanus L.
Asteraceae
Cereal fields. In the whole territory: from R to C. (0-1500 m). Sub-cosmopolitan.
2n=24
Blue: flower
PAL – 10735

Centaurea jacea L.
Asteraceae
Bushes, pine forests, arid scrubland. In all the territory except Sardinia. (0-1000 m). Eurasian.
2n= 22, 44
Yellow: grass
PAL – 69110

Centaurea nigra L.
Asteraceae
Woodlands, hedges, clearings, pastures. Western Alps from Valsesia to Liguria; also suitable for the Tuscan-Emilian Apennines, mountains of Lazio and Abruzzo, but probably by mistake: R. (100-1500 m). W-European.
2n= 22, 44
Green: leaves, stem
PAL – 31437

Centaurea solstitialis L.
Asteraceae
Uncultivated, vineyards, along the streets. In the whole territory; Liguria, Peninsula (north to via Emilia), Sicily, Sardinia, and smaller islands: CC; southern edge of the Alps: R is often only sighted; missing in Padania. (0-1400 m). Sub-cosmopolitan.
2n=16
Yellow: flower
PAL – 10852

Centaurium erythraea Rafn
Gentianaceae
Meadows and uncultivated. In whole territory: CC (0-1000 m). Mediterranean.
2n=(20) 40 (42)
Yellow: flower
PAL – 69489

Cercis siliquastrum L.
Fabaceae
Thermophilic broadleaf woods. Especially in the area of *Quercus pubescens* (preferably calcareous); also, widely cultivated as an ornamental and wild plant. In the whole territory (except Sardinia), but certainly spontaneous only in central Italy: C. (0-800 m). S-European-W-Asian (Pontic).
2n=14
Yellow: branches
PAL – 69557

Chelidonium majus L.
Papaveraceae
Walls, ruins. In the whole territory: C. (0-1200, rarely 1600 m). Eurasian became Circumboreal.
2n=12
Yellow: roots, sap
Blue: grass
PAL – 67480

Chenopodium vulvaria L.

Chenopodiaceae
Edges of streets, uncultivated. In the whole territory: R. (0-800, in Sicily up to 1850 m). European.
2n=18
Yellow: whole plant
PAL – 58437

Chondrilla juncea L.
Asteraceae
Uncultivated and dry meadows. In the whole territory: Northern Italy R; Southern Italy, central Italy, Sicily, and Sardinia: C. (missing on the alluvial soils of Padania (0-1700 m). Euri-Mediterranean-S-Siberian.
2n=15
Yellow: stem, flower
PAL – 11909

Chrozophora tinctoria (L.) A.Juss.
Euphorbiaceae
Stubble fields, abandoned or at rest, uncultivated. Liguria, Peninsula (north to Cesena, Faenza, Trasimeno, Orbetello), Sicily, Sardinia, and smaller islands: C in Sicily and neighboring islands, elsewhere: R. (0-700, rarely 1600 m). Mediterranean-Turanian.
2n=22
Blue: fruits juice, flowered bunches
PAL – 68955

Chrysojasminum fruticans (L.) Banfi
Oleaceae
Hedges. Cultivated and wild, especially in northern Italy in a hilly environment. (0-600 m). East Mediterranean.
2n=26
Yellow: young branches
NYBG – 3147100

Cichorium intybus L.
Asteraceae
Along the streets, in the uncultivated and ruins, also weed in the vegetable gardens. In the whole territory: C. (0-1450 m). Cosmopolitan.
2n=18
Green: grass
PAL – 70634

Citrus aurantium L.
Rutaceae
Cultivated. Especially in Sicily and Calabria and up to the Gargano (0-600 m). China-South.
2n=18
Red: peel of ripe fruit
Yellow: unripe fruits
NYBG – 1341913

Citrus medica L.
Rutaceae
Rarely grown.
2n=18 (20)

Yellow: unripe fruits
PAL – 106002

Clematis vitalba L.
Ranunculaceae
Sub-Mediterranean deciduous woods; hedges. In the whole territory, however in the South mostly on the reliefs and in the humid stations: C. (0-1300 m). European-Caucasian.
2n=16
Yellow: lianas
PAL – 70693

Clinopodium acinos (L.) Kuntze
Lamiaceae
Arid pastures (calcareous). Southern slopes of the Alps, Apennines up to Pollino, Sicily and Sardinia: R. (0-1300 m). Euri-Mediterranean.
2n=18
Green: stem, leaves
PAL – 87352

Clinopodium nepeta (L.) Kuntze
Lamiaceae
Arid meadows, the uncultivated, along the walls, but also the screes, the stony grounds, the herbaceous margins of the woods and the Meso-thermophilic shrubs. In whole territory: CC. (0-1400 m). Steno-Mediterranean.
2n=46
PAL – 72134

Clinopodium vulgare L.
Lamiaceae
Broadleaf woods. In the whole territory: C. (0-1500 m). Circumboreal.
2n=20
Yellow: leaves, flowery stem
PAL – 93721

Colchicum autumnale L.
Colchicaceae
Cropped meadows, damp environments, wooded hills. Alps, from the Giulie to the Marittime: C; Padania in the north of the Po, Appennins Ligure, Pavese and Tosco-Emiliano up to Abetone and Cerreto: R. (0-2100 m). Central European.
2n= 36, 38
Yellow: flower
PAL – 56221

Colutea arborescens L.
Fabaceae
Arid slopes, sub-Mediterranean undergrowth (preferably calcareous). In the whole territory (but with large gaps: it is missing in Padania and Venetian Pre-Alps in max part), in Sicily only as adventitia (Etna): R. Often confused with *Emerus majus*, but easily recognizable by the swollen legume etc. (0-1200 m). Euri-Mediterranean (Subpontic).
2n=16
Green: branches
PAL-GR – 51609_GR

Commelina communis L.
Commelinaceae
Uncultivated, woods. Padana Plain, Prealps, from Veneto to Piedmont: C; also in Lazio, Pescara, Cilento and in general around urban centers. (0-600 m). E-Asian.
2n= many are known, from 16 to about 90
Blue: flower
PAL – 79214

Consolida ajacis (L.) Schur
Ranunculaceae
Fields. Southern Piedmont, Liguria and the Peninsula (north to Lunigiana and Gargano), Sicily: C; probably native only in the western regions; in northern Italy, Marche, Umbria and Abruzzo escaped from cultivation and naturalization. (0-600 m). Euri-Mediterranean.
2n= 16, 18
Green: leaves, flowery stem
PAL – 69496

Convallaria majalis L.
Asparagaceae
Woods and woods. Alps, especially in deciduous forests, more rarely in pine and spruce forests: C; Padania, Northern Apennines, Lazio, Abruzzo, Campania: R; reported anciently in Basilicata, but not found later. (0-1200 m). Circumboreal.
2n=38
Yellow: roots, leaves
PAL – 90932

Convolvulus arvensis L.
Convolvulaceae
Vegetable gardens, vineyards, uncultivated land. In the whole territory: CC. (0-1500 m). Paleo-temperate became Cosmopolitan.
2n=50
Brown: stem, leaves, flower
PAL – 54911

Convolvulus sepium L.
Convolvulaceae
Wet woods, embankments, reeds, wet meadows, hedges, uncultivated. In the whole territory: C in northern and central Italy; progressively rarefied in southern Italy and the islands. (0-1400 m). Paleo-tempered.
2n= 22, 24
Red: roots
PAL – 55072

Coriaria myrtifolia L.
Coriariaceae
Arid slopes, shores, cliffs (calcareous). Western Liguria: C; Parmigiano Apennines in the Manubiola valley and along the Taro to Oppiana. An isolated station on the Euganei. Friuli adventitia. (0-900 m). W-Mediterranean.
2n= 72, 80
Black: whole plant
PAL-GR – 102170_GR

Cornus mas L.
Cornaceae
Sub-Mediterranean broad-leaved woods. Northern Italy (missing in the Pad.), Peninsula up to the Pollino: R. (0-1400 m). SE-European-Pontic.
2n= 18, 27
Yellow: roots' bark
PAL – 85349

Cornus sanguinea L.
Cornaceae
Broad-leaved woods (oaks, chestnut trees), hedges. In the whole territory: C. (0-1300 m). Eurasian-Temperate.
2n=22
Yellow: branches
Green: bark
Purple: berries
PAL – 80568

Coronilla valentina subsp. *glauca* (L.) Batt.
Fabaceae
Limestone cliffs, garrigues. Liguria, Peninsula, Sicily and Sardinia: R. (0-1500 m). SW-Mediterranean.
2n=12
Green: stem, leaves
PAL – 3789

Corylus avellana L.
Betulaceae
In the undergrowth of deciduous and coniferous forests, woodland margins. In all the territory frequently cultivated. (0-1700 m). European-Caucasian.
2n= 22, 28
Green: bark, branches
PAL – 65156

Cota tinctoria (L.) J. Gay subsp. *tinctoria*
Asteraceae
Marly arid slopes (preferably calcareous). Liguria, central and southern Italy up to Campania: C; to the north of the Po in some areas (Colli Eug., Prealpi Lombarde): C; elsewhere (Southern Piedmont, Trentino): R; in alpine valleys, generally as an ephemeral adventitia. (0-1500 m). Central European-Pontic.
2n=18
Yellow: grass, flower
PAL – 29062

Cotinus coggygria Scop.
Anacardiaceae
Bushes and cliffs (calcareous). Northern Italy from the Carso Triestino to Lombardy and Canton Ticino: C. southern Piedmont (up to the Val di Susa), the Apennines in Liguria, Emilia-Romagna, Marche and Umbria, Spoleto, Narni, Sabina, M. della Tolfa, M. Gennaro and to the Agro Pontino:

R. to the south goes as far as the L'Aquila area, Gole di Popoli, Marsica, sporadic in Tuscany in Versilia, towards Florence and Siena. (0-900 m). S-European-Turanian.
2n=30
Red: roots' bark
Yellow: branches, leaves, bark
PAL-GR – 51851_GR

Crataegus monogyna Jacq.
Rosaceae
Bushes, hedges, sub-Mediterranean woods (mixed oak wood, ostereti) above all on the margins and in the clear sky (preferably calcareous). In the whole territory: C. (0-1500 m). Paleo-tempered.
2n=34 (51)
Red: wood
PAL – 85735

Crocus biflorus Mill.
Iridaceae
Dry pastures, meadows. Southern edge of the Alps from Veronese to Bergamasco, Piedmont, Liguria, Peninsula, and Sicily: R. (0-1200 m). Endemic.
2n=8
Yellow: stigmas
PAL – 85932

Crocus imperati Ten.
Iridaceae
Mountain meadows and grassy places near the sea. Southern parts of the Peninsula: Capri, Sorrento Peninsula, Taburno, Avellinese, Potenza: R; also reported in Umbria (Spoleto), Lazio in Sperlonga and Pollino, but to be confirmed. (0-1400 m). Apennine Endemic.
2n=26
Yellow: stigmas
PAL – 86221

Crocus longiflorus Raf.
Iridaceae
Dry and stony pastures, coppices. Sicily, Egadi and Malta: C; Southern Italy: R in the Salerno area (M. della Stella, M. Sacro), Murge, Basilicata in Serraneta, Pollino, Sila, Rosarno, Mongiana, Serra S. Bruno. (0-1500 m). Subendemic.
2n=28
Yellow: stigmas
PAL – 63952

Crocus sativus L.
Iridaceae
Cultivated as a condiment in Abruzzo in the Navelli plain and Sardinia in S. Gavino Monreale. Rarely subspace (Bolzano, Marche, Umbria, Abruzzo, Sicily), but today almost disappeared. W-Asian.
2n= 20, 22, 24, 28
Yellow: stigmas
PAL – 63967

Crocus thomasii Ten.
Iridaceae

Arid stony pastures. Puglia from the Bari to Porto Cesareo and Otranto: C, also in Basilicata near Matera, Montocchio, M.Foj, Potenza to the Dragonare, Poggi di S. Michele and the Pollino: R. Reported by mistake in the Salernitano and on the Serra S. Bruno. (0-1000 m). Subendemic.
2n=16
Yellow: stigmas
VHLVB - 10 0355308

Crocus vernus (L.) Hill
Iridaceae
Generally fertilized meadows, alpine pastures. Alps from the Giulie to the Marittime: C; the reports for the northern and central Apennines, Puglia and Calabria are certainly erroneous. (600-2400 m). SE-European orophyte.
2n=8
Red: flower
Yellow: stigmas
PAL – 86415

Crucianella latifolia L.
Rubiaceae
Arid and stony slopes. Liguria, western coasts from the Argentario to the Circeo, southern Italy, Sicily (Catania, Avola), Sardinia and many smaller Islands: R; also near Florence and Tivoli, Abruzzo. (0-500 m). Steno-Mediterranean.
2n=44
Red: roots
PAL – 59241

Cruciata laevipes Opiz
Rubiaceae
Uncultivated, edges of streets, meadows, forest edges with rich soils. In all the territory, generally C, but R in Sicily (0-1500 m). Eurasian.
2n=22
Red: roots
PAL-GR – 53532_GR

Cupressus sempervirens L.
Cupressaceae
Cultivated for ornamentation and reforestation. Native to the Aegean, widespread throughout the territory, but especially in Liguria, the Peninsula, Sicily, Sadregna and smaller islands: C. (0-800 m). E-Mediterranean (Euri-).
2n=22
Yellow: branches
PAL – 69909

Curcuma longa L.
Zingiberaceae
Rhizomatous, herbaceous plant native to South-East Asia (India).
2n=63
Orange-red: rhizome
NYBG - 1505666

Cuscuta epithymum (L.) L.
Convolvulaceae

On numerous herbaceous and woody plants. In the whole territory: C. (0-1500, max 2000 m). Temperate Eurasian.
2n= 14(16, 28, 30, 32)
Red: whole plant
PAL – 55653

Cuscuta europaea L.
Convolvulaceae
On nettle, hop, elder and other plants of the uncultivated. In the whole territory: C. (0-800, rarely 1800 m). Paleo-tempered.
2n=14
Red: whole plant, sap
PAL – 55617

Cyclamen hederifolium Aiton subsp. *hederifolium*
Myrsinaceae
Holm oak and deciduous woods (oaks, chestnut groves). Liguria, the Peninsula (north to the Via Emilia), Sicily, Sardinia, and smaller islands: C; Veneto, Trentino, Brescia to La Maddalena (RR and not recently observed), Turin to Revigliasco (adventitia?). (0-1300 m). N-Mediterranean (Steno-).
2n=34
Red: tuber
PAL – 63067

Cyclamen purpurascens Mill. subsp. *purpurascens*
Myrsinaceae
Woods, especially beech woods. Alps on southern chains from Gorizia to Valsesia: C; Trieste Karst, pre-Alpine reliefs, high Pianura Padana, Colli Euganei, western Alps from Ivrea to Liguria and Bologna to Barbiano: R. (0-1900 m). NE-Mediterranean orophyte.
2n=34
Red: tuber
PAL-GR – 54530_GR

Cyclamen repandum Sm.
Myrsinaceae
Shady holm oak woods and bush scrub although rarely found in deciduous woods. Peninsula, Sicily Sardinia: C. (0-1200 m). N-Mediterranean.
2n=ignote
PAL – 63071

Cydonia oblonga Mill.
Rosaceae
Cultivated for its fruit throughout the Mediterranean and sub-Mediterranean area; sub-spontaneous in the Peninsula and in the Islands. (0-1500 m). SW-Asian.
2n=34
Yellow: leaves
PAL – 71255

Cynara cardunculus L.
Asteraceae
Cultivated and spontaneous in arid meadows, fallows, and edges of the roads. Present in all Regions south of the Po river: C. (0-1100 m) Steno-Mediterranean.

2n=34
Green: leaves
PAL – 69058

Cynara scolymus L.
Asteraceae
Alien in Friuli, central and southern Italy: C. (0-1100 m) Steno-Mediterranean.
2n=34
Yellow: leaves
Grey: bracts
VHLVB - 14966-010

Cynomorium coccineum L.
Cynomoriceae
Parasyte plant, brackish coastal areas. Sicily, Sardinia, Basilicata: C. (0-50 m.) Steno-Mediterranean.
2n=28
Red: whole plant
PAL-GR – 61146_GR

Cytisus hirsutus L.
Fabaceae
Meadows, heaths, thickets and coppices (preferably acidophilous). Alpine foothills and hills throughout northern Italy and Tuscany: C; present along the Apennine ridge with subsp. polytrichus. (0-1500 m). Central European.
2n=50
Green: leaves, branches
PAL – 91809

Daphne gnidium L.
Thymelaeaceae
Evergreen stain. Western coasts from Liguria to Calabria, Ionian coasts of Basilicata, Puglia, up to the Gargano, Sicily, Sardinia, and smaller islands: C; also, reported in Teramese in Tossicia. (0-800 m). Steno-Mediterranean-Macaronesian.
2n=18
Yellow: leaves, bark
Green: bark
PAL – 61284

Daphne laureola L.
Thymelaeaceae
Broad-leaved woods on mildly acid soils (oak, chestnut, more rarely beech or holm oak). In the whole territory: R. is missing from Padania and in large part of the evergreen Mediterranean belt. (300-800, in Abruzzo up to 1200, in Sicily up to 1500 m). Sub-Mediterranean-sub-Atlantic.
2n=18
Yellow: wood, leaves
PAL – 61304

Daphne mezereum L.
Thymelaeaceae

Beech woods, chestnut groves, mountain forests and subalpine moors. Alps: C; Northern and central Apennines (and also on the sub-Apennines), Campania, Pollino: R. (500-1800, rarely 100-2100 m). Eurosiberian.
2n=18
Green: stem
PAL – 61687

Daucus carota L.
Apiaceae
Uncultivated, anthropized environments and dry grassy sites. Throughout the national territory: CC. (0-1400 m). Sub-cosmopolitan.
2n=18
Red: sterile flower
Green: leaves, stem
PAL – 22061

Daucus carota subsp. *maximus* (Desf.) Ball
Apiaceae
Uncultivated. Peninsula (absent in the Marche), Sicily and Sardinia: R.
2n=18
Blue: pistils
PAL – 22073

Delphinium consolida L.
Ranunculaceae
Commensal in cereal crops (calcareous). In the whole territory excluding the Islands: C; but in recent years in many places it has become R. (0-1200 m). Euri-Mediterranean. (Archaeophyte?)
2n=16
Blue: corolla
PAL-GR – 100342_GR

Dioscorea communis (L.) Caddick & Wilkin
Dioscoreaceae
Dense woods, coppices, clearings, hedges. In the whole territory: C. (0-800, in Sicily up to 1400 m). Euri-Mediterranean.
2n=48
Yellow: berries
PAL – 76324

Diospyros lotus L.
Ebenaceae
Cultivated for wood and fruit and rarely run wild in northern and central Italy and up to Campania: R. (0-500 m). Asian.
2n=30
Yellow: branches, leaves
NYBG – 1018020

Diphasiastrum alpinum (L.) J. Holub
Lycopodiaceae
Pastures, blueberry heaths, dwarf shrubs, clearings on acid substrate. Alps from Giulie to Val di Susa: C; Cuneese in Val Pesio, Northern Apennines: R. (1000-2800 m). Circumboreal.
2n=46

Yellow: whole plant
VHLVB - 20 0002040

Diphasiastrum zeilleri (Rouy) J. Holub
Lycopodiaceae
Sparse deciduous woods, paths, moors, on siliceous substrate. At Scopello in Valsesia: RR. (650-1350 m). Circumboreal. (Euro-American).
2n=46
Yellow: whole plant
VHLVB - 20 0079395

Dipsacus fullonum L.
Dipsacaceae
Uncultivated, ruins, rubble, along the streets and ditches. In the whole territory, generally within the area of the vine: C; it is lacking in the high mountains and in Padania. (0-1400 m). Euri-Mediterranean.
2n=18
Grey: roots
PAL – 93697

Dittrichia viscosa (L.) Greuter subsp. *viscosa*
Asteraceae
Uncultivated fields, ruins, roadsides, edges of streams, preferably on a calcareous substrate. In the whole territory, generally within the area of the vine (0 - 800 m). Euri-Mediterranean
2n=18
Black: whole plant together with stems and leaves of *Rhus coriaria* and peel of *Punica granatum*
PAL – 8762

Echium vulgare L.
Boraginaceae
Uncultivated and dry pastures. In the whole territory: C. (0-1700 m). European.
2n=32
Green: leaves, stem
Grey: roots
PAL – 69766

Elaeagnus angustifolia L.
Elaeagnaceae
Cultivated by ornament especially in northern Italy, introduced on the beaches (Veneto and Friulano coasts, Marche, Lazio, and Abruzzo) to consolidate the dunes, which has become invasive. (0-600 m). Temperate Asian.
2n=28
Red: leaves, branches
S-LINN - 63.11

Erica arborea L.
Ericaceae
Thickets, coppices of holm oak, garrigues (silica or acidified soils). Liguria, Peninsula, Sicily, Sardinia, and smaller islands: C; Alps, from Garda to Lake Como and Eugeni Hills: R. (0-1200, in the North only 0-600 m). Steno-Mediterranean-Atlantic (S-African).
2n=12
Red: branches

PAL – 62860

Erodium moschatum (L.) L'Hér.
Geraniaceae
Uncultivated, vineyards, along the streets. Liguria, Piedmont, Peninsula, Sicily, Sardinia: C; also in Friuli, Veneto, Trentino, Emilia, Bresciano, but often only naturalized (0-1300 m). Euri-Mediterranean.
2n=20
Yellow: grass
PAL – 75102

Eryngium campestre L.
Apiaceae
Arid pastures (calcareous). In the whole territory: C. (0-1500 m). Euri-Mediterranean.
2n=14, 28
Yellow: leaves, stem, shoots, seeds
PAL – 19179

Erysimum cheiri (L.) Crantz
Brassicaceae
Old walls, castles, cliffs, limestone rocks. Naturalized in Liguria, the Peninsula, Sicily, Sardinia, and the smaller islands: C; also on the southern edge of the Alpine system from Trieste to Piedmont. (0-1000 m). Euri-Mediterranean.
2=12
Green: leaves, stem
PAL – 71194

Euonymus europaeus L.
Celastraceae
Broad-leaved woods (especially oak and chestnut woods), hedges. In the whole territory: C. (0-800, in Sicily 400-1300 m). Eurasian.
2n=64
Red: bark
Yellow: capsule
Black: (charred) branches
PAL – 65291

Eupatorium cannabinum L.
Asteraceae
On mud, wet soils, banks, ruins. In the whole territory: C. (0-1350 m). Paleo-tempered.
2n=20, 40
Red: flowery plant
PAL – 8264

Euphorbia characias L.
Euphorbiaceae
Environments of Mediterranean scrub and garrigue, on arid and sunny soils. Peninsula: C. (0-1000 m). Steno-Mediterranean.
2n=20
Yellow: whole plant
PAL – 87471

Euphorbia cyparissias L.
Euphorbiaceae
Arid, uncultivated meadows. Northern and central Italy on the reliefs: CC; Padana and other plains: R; Southwards to Abruzzo and Capua; an artifact in Sardinia at Ulassai. (0-1500, max 2500 m). Central European.
2n=40
Green: whole plant
PAL-GR – 54451_GR

Euphorbia helioscopia L.
Euphorbiaceae
Uncultivated, dry pastures. In the whole territory: C. (0-1200, max 1800 m). Cosmopolitan.
2n=42
Green: whole plant
PAL – 52122

Euphorbia palustris L.
Euphorbiaceae
Shores of ditches, streams, swamps, especially along the major rivers. Padania: C (but mostly disappeared in the artificial channels); Peninsula (Lucchese, Pisa, Bientina, Maremma, Pontine Marshes, Piana del Sele and still near Pesaro, Pescara, Taranto, and Otranto): R. (0-400 m). Eurosiberian.
2n=20
Yellow: whole plant
PAL – 53150

Euphorbia peplus L.
Euphorbiaceae
Nitrated fertilizers rich in nitrates: weed crops, vegetable gardens, ruins. In the whole territory: C. (0-1700 m). Eurosiberian become Cosmopolitan.
2n=16
Yellow: whole plant
PAL – 52907

Euphrasia officinalis L.
Orobanchaceae
Meadows and pastures. Alps: CC; Padania, Northern and Central Apennines: R; also, traced in southern Italy, but probably due to confusion. Circumboreal.
2n=22
Brown: grass, green capsule
PAL – 101818

Fagus sylvatica L.
Fagaceae
Mesophilic woods. The main component of the broad-leaved mountain forest, throughout the territory (excluding Sardinia), especially in the sub-Atlantic humid area. In the Alps, between 600-1300, in the Apennines between 1000 and 1700 m, in the Gargano at 600-800 m; missing in the Po Valley and in the Mediterranean belt (0-2000 m). European-Center.
2n=24
Yellow: fresh bark, branches, calyxes
PAL – 55755

Fallopia convolvulus (L.) Á.Löve
Polygonaceae
Weed crops, uncultivated. In the whole territory: C. (0-1200 m). Eurasian became Cosmopolitan.
2n=40
Yellow: dry plant
PAL – 68666

Ferula communis L.
Apiaceae
Ruins and edges of roads, generally on clay-limestone soils. Liguria, Peninsula, Sicily and Sardinia: C; sighted in Veneto and Lombardy (0-1000 m). S-Mediterranean (Euri-).
2n=22
Yellow: flower
PAL – 86053

Filipendula ulmaria (L.) Maxim.
Rosaceae
Dry, dry meadows (preferably calcareous). Alps, Padania, Peninsula (above all on the reliefs) up to Abruzzo: C; therefore, progressively rarefied in Campania, Puglia, and Basilicata (southwards up to the Barese and Pollino). (0-1500 m). Central-European-S-Siberian (steppic).
2n=14 (16)
Yellow: leaves, flowery stem
VHLVB – 1070599

Foeniculum vulgare Mill.
Apiaceae
Arid environments, cliffs, grassy sites and cultivated areas. In most of the national territory (olive and vine area): C. (0-1000 m). Euri-Mediterranean.
2n=22
Yellow: flowery stem
PAL – 94821

Fragaria vesca L.
Rosaceae
Hedges and wooded edges of beech, pine and fir trees, especially in bright areas. In the whole territory: C, but generally on the reliefs. (200-1900, rarely 0-2400 m). Eurosiberian become Cosmopolitan.
2n=14
Red: roots
Yellow: fruit
PAL – 23642

Frangula alnus Mill. subsp. *alnus*
Rhamnaceae
Broad-leaved woods, especially in damp environments. Northern Italy and the Tuscan Apennines, Marchigiana, Umbro, Abruzzese, Tyrrhenian coasts from Versilia to Agro Pontino and Eboli: R. (0-1300 m). Central European-Caucasian.
2n=22
Yellow: bark, berries, leaves
PAL-GR – 102209_GR

Fraxinus excelsior L.

Oleaceae
Riparii woods, wet gorges. Northern Italy: C; Central Italy: R; dubious in southern Italy. (0-1500 m). European-Caucasian.
2n=46
Yellow: fresh wood
Blue: bark
PAL – 100844

Fraxinus ornus L.
Oleaceae
Degraded scrubland in the sub-Mediterranean area. In the whole territory: C or R. (0-1400 m).
2n=46
Yellow: leaves
PAL – 57151

Fritillaria imperialis L.
Liliaceae
Cultivated by ornament and sub-spontaneous in gardens. W-Asian.
2n=24
Yellow: whole plant
S-LINN - 139.17

Fumaria officinalis L.
Papaveraceae
Cultured and pruned crops (especially potatoes), vegetable gardens, vineyards, ruins. In the whole territory: CC. (0-1600 m). Paleo-temperate, now Cosmopolitan.
2n=14, 28, 32, 48
Yellow: grass
PAL – 78085

Galeopsis ladanum L.
Lamiaceae
Stones, scraps, gravels, ruins, even weeds in cultivated areas. Northern Italy and the Peninsula (on the Tyrrhenian and Abruzzo sides) up to the Sila: R. (0-1000, max 2300 m). Eurasian.
2n=16
Red: flowery grass
PAL – 36752

Galeopsis tetrahit L.
Lamiaceae
Fields, rubble, debris (nitrophilic). Northern Italy, Central Italy, Campania: C; Basilicata in Muro and Pollino: R. (0-2000 m). Eurasian.
2n=32
Red: flowery grass
PAL – 36793

Galium aparine L.
Rubiaceae
Uncultivated, hedges, woods. In the whole territory: CC. (0-1700 m). Eurasian.
2n=22, 44
Red: roots
Red purple: roots

PAL – 59387

Galium boreale L.
Rubiaceae
Wet meadows and woods (preferably calcareous). Alps, from the Carniche to the Marittime: R; once also in the upper Po Valley, but almost everywhere disappeared. (600-1600, rarely 100-2200 m). Circumboreal.
2n=44
Red: roots
PAL – 90638

Galium mollugo L.
Rubiaceae
Thick meadows, damp woods. Alps, from Carnia to Liguria: R; also, reported in the Emilian Apennines and the central Apennines (0-1000 m). Euri-Mediterranean
2n=22
Red: roots
Yellow: stem, leaves
PAL – 87462

Galium sylvaticum L.
Rubiaceae
Mesophilous broadleaf woods, oak and hornbeam forest. Northern Italy: R; reported in the mountains of Tuscany and (less likely) than southern Italy. (0-1100 m). Central European.
2n=22
Red: roots
PAL – 59674

Galium verum L.
Rubiaceae
Dry meadows, woods. In the whole territory: C. (0-1700 m). Eurasian.
2n=22, 44
Red: roots
Yellow: flowery grass, spikes
PAL – 59738

Genista pilosa L.
Fabaceae
Acidophilous and subtermophilic, preferential and almost exclusive species of marly-arenaceous substrates. Southern edge of the Alps from the Trieste Karst to Piedmont, Liguria, the Apuan Alps, the Northern Apennines (eastward to the Sasso di Castro and Vallombrosa), southern Tuscany in the Siena and Argentario, Elba, central Apennines (Umbria, Lazio, and Abruzzo), southern Apennines in Basilicata, doubtful in Campania: R. Also, reported in the Pollino (perhaps confused with *G. sericea*). (100-1500 m) Central European (Sub-Atlantic).
2n=24, (40, 42) 44
Yellow: flowery branches
PAL – 89190

Genista tinctoria L.
Fabaceae

Sub-Mediterranean woods (oak, chestnut, pine) and mantle formations, heaths, dry meadows (preferably acidophilous). In the whole territory (excluding Sicily and Sardinia): C. (0-1800 m). Eurasian.
2n=48, 96
Yellow: stem, flowery branches
PAL – 91458

Geranium columbinum L.
Geraniaceae
Uncultivated, pastures, along the streets. In the whole territory: C. (0-1200). European-S-Siberian (sub-steppic).
2n=18
Yellow: grass
PAL – 74422

Geranium robertianum L.
Geraniaceae
Shady environments (walls, hedges, woods, caves). In the whole territory: C; in the Peninsula and Islands especially in the mountains. (0-1600 m). Sub-cosmopolitan.
2n=64
Yellow: grass
PAL – 68430

Glebionis coronaria (L.) N.N.Tzvel.
Asteraceae
Fields, vineyards, olive groves, uncultivated land. Liguria, Peninsula, Sicily and Sardinia and smaller islands: C; Umbria, Piedmont and Val d'Aosta, absent. (0-600 m). Steno-Mediterranean.
2n=18
Yellow: whole plant
PAL – 87466

Glebionis segetum (L.) Fourr. i
Asteraceae
Fields, vineyards, olive groves, uncultivated land. Liguria, Peninsula, Sicily and Sardinia and smaller islands: C; in northern Italy: RR, and only as an ephemeral adventitia (Trentino, Val d'Aosta). (0-800 m). Steno-Mediterranean-Turanian became Euri-Mediterranean.
2n=18
Yellow: whole plant
PAL – 87280

Gleditsia triacanthos L.
Fabaceae
Cultivated in hedges and gardens and sub-spontaneous in the inhabited areas. Northern Italy, Tuscany, Lazio, Marche, and Sicily: C. (0-500 m). N-American.
2n=28
Red: young branches, thorns
PAL – 91003

Gymnadenia nigra (L.) Rchb.f.
Orchidaceae
Range not well defined.
Purple: flower

PAL – 102682

Hedera helix L.
Araliaceae
Broad-leaved and shrub woods, also commonly cultivated for ornamentation. In the whole territory: CC; sometimes R. Especially in districts with an oceanic climate and in a Mediterranean environment. (0-1450 m). Submediterranean-sub-Atlantic.
2n=48
Black: leaves
Dark green: leaves and fruit
PAL – 62853

Helianthemum nummularium (L.) Mill. subsp. *nummularium*
Cistaceae
Dry meadows, sands, cliffs (calcareous). In the whole territory: C, but it is missing in Padania and in the other flood plains. (0-2500 m). European-Caucasian.
2n=20
Brown: roots, stem
PAL – 100850

Helianthus annuus L.
Asteraceae
Farm, roadsides, uncultivated, rubble. In the whole territory (excluding Val d'Aosta, Puglia, and Sardinia): random in most of the regions, naturalized in Friuli and Umbria. (0-1500 m). N-American.
2n=34
Yellow: flower
VHLVB - 10 0240564

Helichrysum italicum (Roth) G. Don
Asteraceae
Garrigues, bushes, dry meadows. It is common in the central, south Italy and in the islands; sporadic in the pre-Alpine area (0-800 -1.500). S-European
2n=28
Light Yellow: leaves, flowery stem
PAL – 93676

Helleborus foetidus L.
Ranunculaceae
Woodland edges, coppices. Alps from Veneto to Trentino (missing to the east of the Adige) to Liguria and northern and central Apennines as far as the Marche and Umbria: C; also in Sardinia. (0-1000, rarely 1800 m). Sub-Atlantic.
2n=32
Yellow: leaves, flowery stem
PAL-GR – 64021_GR

Hibiscus syriacus L.
Malvaceae
Grown for hedges in many varieties. In the whole territory, often sub-spontaneous: C. (0-600 m). E-Asian.
2n=88
Red: wood

Purple: flower
PAL – 108019

Hieracium umbellatum L.
Asteraceae
Woods (oaks, chestnut woods), moors, uncultivated, on generally acid soil. Prealpine Alps and hilly reliefs from Trieste to Liguria: C; also in Emilia Romagna, Tuscany, Umbria, Lazio, Basilicata and Campania: RR. (0-1500 [-1900] m). Circumboreal.
2n=18, 27
Yellow: grass
PAL – 32503

Hippophae rhamnoides L.
Elaeagnaceae
Riverbeds, landslides, gullies (often also introduced to stabilize the soil). Alps, Padania, Northern Apennines (especially on the N side) up to the valleys of the Foglia and Arno rivers: C; also in Umbria (M. Valmeronte) and Campania in Policastro. (50-1700 m). Eurasian temperate.
2n=24
Red: branches
H.PAOL - 3508

Humulus lupulus L.
Cannabaceae
Wet woods, hedges. In the whole territory; Northern Italy and the Apennines as far as Abruzzo: C; in the Mediterranean area in Sicily and Sardinia: R. (0-1200 m). European-Caucasian or Circumboreal.
2n=20
Red: leaves, flowery stem
PAL – 89011

Hypericum perforatum L.
Hypericaceae
Arid meadows, scrublands, edges of woods, along the streets, uncultivated. In the whole territory: C. (0-1600 m). Sub-cosmopolitan.
2n=32
Yellow: flower, flowery plant
Red: flower
PAL – 79061

Ilex aquifolium L.
Aquifoliaceae
Spontaneous in deciduous forests. Throughout the national territory, but intensely cultivated for wood and leaves and now rare in the spontaneous state: R; frequently cultivated in parks and gardens. (0-1400 m). Sub-Mediterranean-sub-Atlantic.
2n=40
Yellow: leaves, young branches
PAL – 65260

Impatiens balsamina L.
Balsaminaceae
Commonly cultivated and rarely sub-spontaneous. Northern Italy: R. (0-1000 m). SE-Asian.
2n=14, 18, 20, 24

Yellow: flower
VHLVB - 10 0132325

Indigofera tinctoria L.
Fabaceae
Cultivated.
2n=16
Blue: leaves
S-LINN - 314.3

Inula helenium L.
Asteraceae
Moor woods, coppices, ditches. Northern Italy (Piedmont, Veneto, Liguria, Emilia-Romagna; non-native in Friuli); Central and southern Italy (Umbria, Abruzzo, Molise, Campania, Puglia, and Basilicata; non-native in Marche and Lazio); dubious in Tuscany. (500-1200 m). SE-European orophyte.
2n=10
Blue: roots
PAL – 28199

Iris germanica L.
Iridaceae
Old walls, field margins, dry places, and cliffs. Cultivated for ornament and run wild throughout the territory; in the North: C; elsewhere, R is often referred to by mistake. (0-1200 m). Euri-Mediterranean. Unknown origin.
2n= (24, 34, 36), 44, (48, 60).
Green: flower
PAL – 64603

Iris pseudacorus L.
Iridaceae
Ditches, banks, marshes. In the whole territory: C. (0-300, max 1000 m).
2n=34 (24, 30, 32)
Yellow: roots
PAL – 64622

Isatis tinctoria L.
Brassicaceae
Uncultivated dry. In Italy, it has two distinct areas: Western Alps from the Val d'Aosta to the Maritime Alps, and Liguria: C; Peninsula (north to the Esino Valley at the Gola della Rossa, Visso, Norcia, Marsica, Capua), Sicily and Sardinia: C. Elsewhere: RR (near Bolzano, Langhe at Ceva, Pietra Parcellara, Coli, near Florence, Lazio at Tuscolo) or adventitious transient (Treviso) and absent on large tracts. (0-2100 m) SE-Asian (steppic).
2n=28
Blue: leaves
PAL – 100675

Jacobaea paludosa (L.) "G.Gaertn., B.Mey. & Scherb."
Asteraceae
Oligotrophic swamps. Alps and Po-Veneto plains: once widespread, today almost everywhere extinct: RR; recently reported in the Trieste area (0-600 m). Euro-Siberian.
2n=40

Yellow: flowery stem
PAL – 30145

Jacobaea vulgaris Gaertn.
Asteraceae
Arid meadows (calcareous). Northern Italy, Tuscany, Umbria, Campania, and Calabria: R; dubious in the Marche, Abruzzo, and Basilicata. (0-1500m). Paleo-tempered.
2n=20, 40
Green: leaves, flowery stem
VHLVB - 10 0495702

Jasminum officinale L.
Oleaceae
Hedges. Cultivated for ornamentation and feral in northern Italy, the Peninsula and Sicily. (0-800 m). SW-Asian.
2n=26
Yellow: young branches
PAL – 57425

Juglans regia L.
Juglandaceae
Cultivated for fruit and wood throughout the territory and frequently adventitia. (0-1200 m). SW-Asian.
2n=32
Yellow: bark, catkin, husk
Brown: roots' bark, leaves
H.PAOL - 1674

Knautia arvensis (L.) Coult.
Dipsacaceae
Arid, uncultivated pastures, woodlands. Alps from Carnia to Liguria, Tuscany (above all on the reliefs) up to Basilicata: C; in southern Italy in Campania, Puglia and Basilicata; dubious in Lazio, it is missing from the Marche to Abruzzo where it has often been indicated, but by mistake. (0-2000 m). Eurasian.
2n=20, 40
Yellow: leaves, dry flower
PAL – 65992

Laburnum anagyroides Medik.
Fabaceae
Broad-leaved woods (especially oaks and chestnuts), shrubs and woodlands. Northern Italy (only in the hilly area, missing in the high continental mountain valleys and in Padania): R; Central and southern Italy up to Pollino (doubt in Puglia): C; In Sicily, it is widely used for reforestations and sometimes sub-spontaneous. (0-800 m, rarely more). S-European.
2n=48 + 0-2B
Green: leaves
PAL – 84053

Lamium purpureum L.
Lamiaceae
Fields, vegetable gardens, vineyards, ruins. Northern and central Italy: C; Southern Italy and Sardinia: R. (0-1500, at Gr. S. Bernardo up to 2476 m). Eurasian.

2n=18
Green: flowery stem
PAL – 36705

Lathyrus aphaca L.
Fabaceae
Weed in wheat fields (calcareous), uncultivated. In the whole territory; in the Mediterranean area: C; on the findings: R; missing in the Padanian irrigated areas. (0-1500 m). Euri-Mediterranean.
2n=14
Green: fresh plant
PAL – 70675

Laurus nobilis L.
Mesophilic species on fresh arenaceous substrates, in the olive area. In the whole territory (0-800 m). Steno-Mediterranean.
2n=42
Greenish: fresh leaves
PAL – 87895

Lavandula angustifolia Mill.
Lamiaceae
Low spot and garrigue. Liguria (from where it goes back to Piedmont, in the Maritime Alps and Cozie), Coasts of Tuscany: R; commonly cultivated and sub-spontaneous throughout the territory. (0-1800 m). Steno-Mediterranean-West.
2n=54
Brown: stem, branches
VHLVB - 10 0355135

Lavandula angustifolia subsp. *pyrenaica* (DC.) Guinea
Lamiaceae
Low spot and garrigue. Liguria (from where it goes back to Piedmont, in the Maritime Alps and Cozie), Coasts of Tuscany: R; commonly cultivated and sub-spontaneous throughout the territory. (0-1800 m). Steno-Mediterranean-West.
2n=54
Brown: stem, branches
PAL – 36189

Leopoldia comosa (L.) Parl.
Asparagaceae
In whole territory: C. (0-1300 m). Euri-Mediterranean.
2n=18
Blue: flowers
PAL – 73387

Ligustrum vulgare L.
Oleaceae
Thermophilous deciduous woods, especially on the edges and in degradation shrubs, hedges. Alps, Padania, Peninsula up to Pollino: C; also in Sicily, but of indigenous doubt. (0-1300 m). European-Asian-W.
2n=46
Red: berries
Yellow: bark

PAL – 57326

Limonium virgatum (Willd.) Fourr. subsp. *virgatum*
Plumbaginaceae
On the coasts, both in brackish and rocky cliffs, also synanthropic. Adriatic coast from Monfalcone to Romagna, Lazio between Anzio and Gaeta, Abruzzo, southern Italy and Sicily, Sardinia, and many smaller islands: C; it is missing on the western coasts from Liguria to the Tiber, in the Marche and in Molise. (0-200 m, but always on the coast). Steno-Mediterranean.
2n=27
Blue: flower
PAL – 85060

Linaria vulgaris Mill. subsp. *vulgaris*
Plantaginaceae
Uncultivated, ruins, rubble, embankments. Northern Italy and Peninsulas: C; Sicily and Sardinia: RR. (0-1500 m). Eurasian.
2n=12
Yellow: stem, flower
PAL – 88671

Linum usitatissimum L.
Linaceae
Cultivated and sub-spontaneous. Throughout the territory, but in the process of disappearance. (0-2000 m).
2n= 30, 32
Black: soot
PAL – 90934

Liriodendron tulipifera L.
Magnoliaceae
Native to the eastern USA (from Florida to the state of New York), cultivated for ornamentation especially in Padania and Insubria. N-American.
2n=114
Yellow: young branches, leaves
S-LINN - 222.1

Lonicera alpigena L.
Caprifoliaceae
Cedui, clearings, humid scrub, especially in beech-woods and vegetation types by these dependents. Alps from the Giulie to Liguria: C (but preferably in the districts with an oceanic climate); Trieste Karst, Apuan Alps, Tuscan-Emilian Apennines, Central Apennines, Campania, and Basilicata, up to Pollino: R. (800-2100 m). Orophyte-S-European (sub-Illyrian).
2n= 18, 36
Yellow: young branches
H.PAOL - 2680

Lonicera caprifolium L.
Caprifoliaceae
Deciduous woods (oaks, chestnut trees), woods, hedges, vineyards. Northern and central Italy: C; Southern Italy up to the Pollino: R; missing in the major islands. (0-1200 m). SE-European (Pontic).
2n=18
Yellow: young stem

H.PAOL - 2675

Lonicera periclymenum L.
Caprifoliaceae
Broad-leaved woods on acid soil. Piedmont (Val Sesia, Val Aosta), Bormiese, Western Liguria, Tuscany (M. Pisano, Maremma, Argentario, Amiata): R; also, reported in Veneto at Bassano, Marche, in Irpinia and near our borders in Canton Ticino and in Istria in Piran, but to be verified. (500-1400 m). W-European (Subatlantic).
2n=18, 36, 54
Yellow: young stem
PAL – 96199

Loranthus europaeus Jacq.
Loranthaceae
Woods. Hemi-parasite on deciduous oaks, more rarely also on cork oak and chestnut. Peninsula from Emilia to Calabria and Sicily: R; also on the Trieste Karst. (0-800 m). European-Caucasian.
2n=18
Red: wood
PAL – 66315

Lotus corniculatus L.
Fabaceae
Mostly in environments created by man (mowed and fertilized meadows, dry pastures, uncultivated grassy areas, also cultivated as fodder). In the whole territory: CC (in Sicily only rarely as a species introduced with fodder seeds). (0-1800 m). Paleo-temperate became Cosmopolitan.
2n=24
Blue: dry flowers
PAL – 96625

Lotus halophilus Boiss. & Spruner
Fabaceae
Wet sands at the coasts. Near Taranto, Policoro, Calabria, Sicily, Linosa: R. (0-300 m). S-Mediterranean.
2n=14
Blue: dry flowers
PAL – 96745

Lotus hirsutus (L.) Ser.
Fabaceae
Thicket, dry pastures. Liguria, Peninsula, Sicily and Sardinia: C; northern limit Ravenna - Bologna - Rubiera - Parma - Apennine ridge; also in western Trentino, Como. (0-1300). Euri-Mediterranean.
2n=14
Yellow: leaves
PAL – 3023

Lotus tenuis Waldst. & Kit.
Fabaceae
Wet meadows especially brackish. In the whole territory on the coasts, sporadically in Padania, Trentino and on the hills of Piedmont; in central-southern Italy and the Islands also inland and on the hills: R. (0-800 m). Paleo-tempered.
2n=12
Blue: dry flowers

PAL – 3401

Lycopodium clavatum L.
Lycopodiaceae
Pastures, blueberry moors, rocky places, woods. Alps: C. Northern Apennines: R. (500-2500 m). Circumboreal.
2n=68
Yellow: whole plant
PAL – 78160

Lycopsis arvensis L.
Boraginaceae
Fallows, fields, roadsides, and ruins. Northern Italy from Friuli (Pordenone plain) to Val d'Aosta, Piedmont and Liguria, Tuscany (Lunigiana, Volterrano, near Florence, but not recently found): R (and in regression); also in Abruzzo on the Piano di Laroma (Chieti) and in Sardinia in Gennargentu: RR. (0-1600 m). Eurasian.
2n=48
Yellow: fresh grass, flower
PAL – 100744

Lycopus europaeus L.
Lamiaceae
Wet meadows, ditches, reeds. (0-1100 m). Paleo-temperate became Circumboreal.
2n=22
Yellow: leaves, stem, flower
Black: grass, sap
PAL – 18107

Lysimachia vulgaris L.
Myrsinaceae
Marshes, ditches, wet woodlands. Northern and central Italy: C; still in the Salernitan area at Padula, near Potenza, on the Pollino and Sicily at Ispica. (0-1200 m). Eurasian.
2n= 28, 42, 84
Yellow: grass, stem, flower
Grey: roots
PAL – 102298

Lythrum salicaria L.
Lythraceae
Shores of ditches, streams, marshes even in a salty environment. In the whole territory: C. (0-1200, rarely 2100 m). Sub-cosmopolitan.
2n= (30), 60
Brown: flowery stem
PAL – 62317

Malus domestica Borkh.
Rosaceae
Commonly cultivated. In the whole territory: C. (0-1500 m).
2n=34, 51
Yellow: bark, leaves
Brown: dry wood
H.PAOL - 1908

Malva arborea (L.) Webb & Berthel.
Malvaceae
Spontaneous on the sea cliffs and wild. Sicily and Sardinia, in the Pontine Islands, Naples and perhaps elsewhere: RR; frequently cultivated and feral in Liguria, central Italy, Campania, Puglia: C; as a transient adventitress also in the N: Comasco in Mandello, Veneto in Rosolina. (0-600 m). Steno-Mediterranean.
2n=40
Yellow: stem, leaves
PAL – 66134

Marrubium vulgare L.
Lamiaceae
Uncultivated, ruins, dry pastures (nitrophilous). In the whole territory, Center-S and Islands: C; to the N especially in the hilly areas; it is almost missing in Padania (0-1200 m). Sub-cosmopolitan.
2n=34
Yellow: stem, flower
PAL – 14211

Matricaria chamomilla L.
Asteraceae
Uncultivated, or commensal in cereal crops. In the whole territory: C. (0-800 rarely 1500 m). Sub-cosmopolitan.
2n=18
Yellow: flower
PAL – 9259

Matthiola incana (L.) W.T.Aiton
Brassicaceae
Maritime cliffs (limestone) and old walls. On the entire west coast from Liguria to Calabria, Ionian and Adriatic coasts north to Conero, Sicily and Sardinia and in the smaller islands: C; elsewhere escaped from cultivation and naturalized (Trieste to Duino, Lake Garda, Lake Como), in the Peninsula also far from the sea, but then mostly introduced. (Coastal, naturalized up to 600 m). Steno-Mediterranean.
2n=14
Green: leaves, stem
PAL – 71361

Matthiola sinuata (L.) W.T.Aiton
Brassicaceae
Maritime dunes. Western coasts, Sicily, Sardinia, Elba, Giglio, Ischia, Pantelleria, Puglia in Otranto and Gallipoli: R. Mediterranean-Atlantic.
2n=14
Red: flower
PAL – 6548

Medicago sativa L.
Fabaceae
Cultivated for forage already in ancient times according to Pliny, then forgotten in the Middle Ages and reintroduced only by 3 centuries, now very common everywhere. Originally probably from Persia (the ancient empire of the Medes). In the whole territory: C. (0-1200, exceptionally 1900 m on the Stelvio). W-Eurasian.
2n=32

Yellow: dry grass
PAL – 75550

Melampyrum nemorosum L.
Orobanchaceae
Oak-hornbeam woods, and dependent associations. Reported in northern and central Italy, but mostly due to confusion, known with certainty only from Brescia to the Bergamasco: R; allied forms throughout the NE and the central Apennines from the Sibillini to Abruzzo, which probably represent a distinct taxon. (0-1000 m). Eurasiat.
2n=18
Green: flowery whole plant
PAL-GR – 54590_GR

Melampyrum pratense L.
Orobanchaceae
Woods on humified acid soil (oak, chestnut, beech, spruce). Alps, from the Giulie to the Marittime: C; also on the Trieste Karst, hills of Friuli and Treviso, Langhe and up to the margins of Padania; an ancient indication for the Bolognese (Gesso, Zola Predosa) should be verified. (100-1800 m). Euro-Siberian.
2n=18
Yellow: whole plant
PAL – 101802

Melia azedarach L.
Meliaceae
Grown in gardens and avenues, occasionally adventitious in neighboring areas. India.
Red: branches
PAL-GR – 102072_GR

Melissa officinalis L.
Lamiaceae
Uncultivated, ruins, often cultivated and wild. In the whole territory: until the nineteenth century, CC, now R and in many areas almost disappeared. (0-1000 m). W-Asian become Euri-Mediterranean.
2n=32
Green: leaves
PAL – 16087

Mentha aquatica L.
Lamiaceae
Embankments, banks, marshes. In the whole territory: C. (0-1200 m). Sub-cosmopolitan.
2n=96
Green: leaves, stem
PAL – 18109

Menyanthes trifoliata L.
Menyanthaceae
Marshes, ponds. Northern Italy (in the Padana Valley everywhere disappeared): R; Appennino Tosco-Emiliano, Valdarno, Umbria in Colfiorito, Paludi Pontine, Abruzzo, Matese and province of Avellino: RR. (0-2000, max 2170 m). Circumboreal.
2n=54
Yellow: grass

PAL – 93271

Mercurialis annua L.
Euphorbiaceae
Infesting the fertilized crops, vegetable gardens, abandoned soils, more rarely on walls and rubble. In the whole territory: C. (0-1300, rarely even up to 1800 m). Paleo-tempered.
2n=16
Yellow: leaves, stem
PAL – 70499

Mercurialis corsica Coss. & Kralik
Euphorbiaceae
Sardinia: R. (0-1100 m). Endemic
2n=ignote
NL - 1353837

Mercurialis perennis L.
Euphorbiaceae
Mesophilous woods, especially beech woods. In the whole territory (excluding Sardinia); Alps, Northern Apennines: C; Peninsula and Islands: R; Padania: RR. (0-1600 m). European-Caucasian.
2n=48-112
Blue: grass
PAL – 85058

Mespilus germanica L.
Rosaceae
Rarely cultivated and wild in broad-leaved woods, on sub-acid soil (chestnut groves, oak forests). In the whole territory: R. (0-1000 m). S-European.
2n=34
Red: bark, wood, branches, fruit
PAL – 84873

Misopates orontium (L.) Raf. subsp. *orontium*
Plantaginaceae
Fields, vineyards, dry uncultivated (preferably silica). In the whole territory, Liguria, Peninsula, and Islands: C; Alps (only on arid slopes): R; Padania: mostly absent. (0-1000 m). Paleo-tempered.
2n=16
Yellow: fruited plant
PAL – 89281

Morus alba L.
Moraceae
Cultivated for the breeding of the silkworm in the whole territory and rarely sub-spontaneous. East Asia.
2n=14, 28
Yellow: wood
Green: leaves
VHLVB - 10 0235951

Morus nigra L.
Moraceae
Cultivated for the fruit throughout the territory and rarely sub-spontaneous. SW-Asia.

2n=28
Yellow: wood
VHLVB - 10 0399290

Muscari atlanticum Boiss. & Reut.
Asparagaceae
In whole territory: CC. (0-600 m). Mediterranean-Turanian
2n= 18, 28, 36, 45, 54, 72
Blue: flower
PAL – 63217

Muscari neglectum Ten.
Asparagaceae
In whole territory: CC. (0-600 m). Mediterranean-Turanian
2n=18, 28, 36, (45), 54
Blue: flower
PAL – 61571

Myricaria germanica (L.) Desv. subsp. *germanica*
Tamaricaceae
Riverbeds of mountain streams and floating up to the plain. Alps, Padania (along the rivers), northern and central Apennines up to Abruzzo: R. (0-2000 m). European-W-Asian orophyte.
2n=12
Black: fruit
PAL-GR – 54601_GR

Myrrhis odorata (L.) Scop.
Apiaceae
Mountain and subalpine meadows. Eastern Alps from the Giulie to the Grigne: C; also in the Langhe, Cozie and Maritime Alps (synanthropic), Appennino Tosco-Emiliano, Alpi Apuane: R. (1000-2100 m). SE-European orophyte.
2n=22
Yellow: leaves, green stem
PAL – 24309

Myrtus communis L.
Myrtaceae
Mediterranean maquis. Liguria, coasts of the Peninsula (on the Adriatic only up to the southern Marche), Sicily, Sardinia, and in almost all the smaller islands: C; as introduced plant and subspontnea still on the Costiera Triestina, Garda, Insubria lakes, Adriatic coasts up to Pesaro and at the mouth of the Po (0-500 m). Steno-Mediterranean.
2n=22
Brown: berries’ juice, leaves
PAL – 62408

Narcissus pseudonarcissus L.
Amaryllidaceae
Cultivated for ornamentation (often in full-flowered forms) and feral in vegetable gardens, vineyards, fields. Spontaneous in southern Piedmont; naturalization in northern Italy, central and southern Italy up to Basilicata: R. (0-800 m). W-European.
2n= 14 (28, 42)
Yellow: flower

PAL – 85606

Nerium oleander L.
Apocynaceae
Riparian woods, river streams. Spontaneous in southern Italy, Sicily, and Sardinia; also in the Ligurian Riviera, Argentario and around Lake Garda, but probably only run wild; elsewhere commonly cultivated and often sub-spontaneous. (0-300 m.) S-Mediterranean.
2n=22
Yellow: branches, leaves
PAL – 63523

Nicotiana tabacum L.
Solanaceae
Cultivated on a large scale throughout the territory. (0-600 m.) N-American.
2n=(24) 48 (96)
Yellow: green leaves
Brown: yellowed leaves
PAL – 101690

Oenanthe pimpinelloides L.
Apiaceae
Marshes, springs, and rarely very moist deciduous forests. In most of the territory (with large gaps especially in the North): from R to C. (0-800- m). Mediterranean-Atlantic.
2n=22
Green: yellowed stem
PAL – 21127

Olea europaea L.
Oleaceae
Spontaneous and cultivated throughout the Mediterranean area. Liguria, Peninsula (up to the hills of Emilia-Romagna), Sicily, Sardinia and smaller islands: C; in the North only cultivated in the Trieste, Montebello, Bassano, Colli Eugeni, Garda, Lombard Prealps, especially on Lake Como, Canton Ticino, Langhe; in many of these areas the crop, after having experienced a phase of decline that lasted until the last decade of the last century, is currently in strong recovery, thanks also to the general increase in average temperatures. Recent installations have even been carried out on the Rhaetian side of the Valtellina. (0-900 m). Steno-Mediterranean.
2n=46
Yellow: young soft branches, leaves
PAL – 57307

Ononis natrix L.
Fabaceae
Dry meadows. Prealpine slopes from western Friuli (Sacile) to Piedmont and the Apennines up to Calabria: C. (0-1200, rarely 1700 m). Euri-Mediterranean.
2n=28, 32
Green: grass
PAL – 71437

Ononis spinosa L.
Fabaceae
Arid environments. In the whole territory, except in Sicily, with various subspecies: C. (0-1300 m). Euri-Mediterranean.

2n=30
Yellow: flowery stem
PAL – 92482

Opuntia ficus-indica (L.) Mill.
Cactaceae
Neophyte invasive. Peninsula, Sicily and Sardinia: CC. (0-1000 m). Neotropical.
2n=ignote

Origanum vulgare L.
Lamiaceae
Sparse bushes, bushes, sunny cliffs. In the whole territory: central and northern Italy: C (missing in Padania); Southern Italy up to the Murge and Pollino, Sicily and Sardinia: R. (0-1400, in Abruzzo up to 1700 m). Eurasian.
2n=30
Brown: top of the flowering plant
PAL – 17531

Paeonia officinalis L.
Paeoniaceae
Screes, woods, meadows. Alps and Apennines up to Abruzzo. (0-1800 m). European-Pontic.
2n=20
Grey: flower
PAL-GR – 100301_GR

Paliurus spina-christi Mill.
Rhamnaceae
Arid slopes, bushes. Northern Italy (excluding the plain), central and southern Italy up to Basilicata and Sicily (near Palermo, Madonie and Iblei); but in most of the Peninsula perhaps only as a wild species: C. (0-500 m). SE-European-Pontic.
2n=24
Yellow: young branches, leaves
PAL – 66031

Papaver rhoeas L.
Papaveraceae
Infesting the cereal fields, often even on ruins and rubble. In the whole territory: C. (0-1950 m). E-Mediterranean always synanthropic.
2n=14
Red: corolla
PAL – 83450

Papaver somniferum L.
Papaveraceae
Cultivated as a medicinal plant (opium poppy), for aromatic seeds (in the Alps) and as an ornamental species. Rarely subspontaneous. (0-1500 m). Euri-Mediterranean which has become Sub-cosmopolitan.
2n=22
Yellow: leaves, young soft stem
PAL – 84175

Parietaria judaica L.

Urticaceae
Roadsides, along dry stone walls and in arid places. Whole territory: CC. (0-1000 m). Euri-Mediterranean
2n=26
PAL – 93704

Parietaria officinalis L.
Urticaceae
Wooded eutrophic soils, rubble, abandoned lands, often nitrophilous and in shady environments. In the whole territory: C, but progressively rarefied in the South and sometimes absent in the Islands. (0-900 m). Central European-Asian-W.
2n=14
Green: leaves, fresh stem
PAL – 66259

Paris quadrifolia L.
Melanthiaceae
Wet deciduous and coniferous woods. Alps, northern and central Apennines: C; also in the Appennino Campano, Sila; in the Padania, almost everywhere extinct. (200-2000 m). Eurasian.
2n=20
Green: leaves
Brown: berries
PAL – 63398

Pelargonium inquinans (L.) L'Hér.
Geraniaceae
Commonly cultivated. S-African.
Red: corolla
VHLVB - 12460-010

Periploca graeca L.
Apocynaceae
Wet woods, hedges. Tuscany between Viareggio and Livorno and up to Cecina, Abruzzo, Otranto to the Almini, Calabria to the Bosco di Rosarno: R; in many places disappearing; cultivated and sub-spontaneous in Trieste. NE-Mediterranean (Steno-).
2n=24
Brown: whole plant
VHLVB - 10 1041013

Persicaria hydropiper (L.) Delarbre
Polygonaceae
Moist and muddy environments, ditches. The whole territory, excluding Puglia and Sardinia; Northern Italy: C; elsewhere: R. (0-1300 m). Cosmopolitan.
2n=20
Yellow: grass
VHLVB - 10 0463008

Persicaria lapathifolia (L.) S. F. Gray subsp. *lapathifolia*
Polygonaceae
Riverbeds and ditches, ruderal and cultivated environments. In the whole territory: CC. (0-1300 m). Cosmopolitan.
2n=22

Blue: leaves
PAL – 76179

Persicaria maculosa Gray subsp. *maculosa*
Polygonaceae
Weeds irrigated crops, ruderal. In the whole territory: CC. (0-1300 m). Cosmopolitan.
2n=22, 44
Yellow: grass
VHLVB - 10 0142723

Phaseolus coccineus L.
Fabaceae
Cultivated mainly for ornament: R. N-American.
Red: flower
VHLVB - 18 0006896

Philadelphus coronarius L.
Hydrangeaceae
Spontaneous in the sub-Mediterranean woods. Eastern Alps from Trevigiano to Bresciano, Tuscany and Abruzzo: R. (0-800 m). Subendemic.
2n=26
Red: branches
VHLVB - 10 0502901

Phillyrea angustifolia L.
Oleaceae
Drought and hard coastal soils. Peninsula, Sardinia: C; missing in Marche and Umbria. (0-500 m). Steno-Mediterranean
2n=ignote
Yellow: branches, leaves
PAL – 92105

Phillyrea latifolia L.
Oleaceae
Stains and holm oaks. Liguria, Peninsula, Sicily and Sardinia and smaller islands: C; in the rest of northern Italy: RR. (0-800 m). Steno-Mediterranean.
2n=ignote
Yellow: branches, leaves
PAL – 57225

Phragmites australis (Cav.) Trin. ex Steud. subps. *australis*
Poaceae
Marshes, banks, embankments, wet areas (even brackish). Throughout the territories: C. (0-1200, maximum 2000 m). Sub-cosmopolitan.
2n= (36) 48 (72, 84, 96)
Green: spikes
PAL – 67874

Physalis alkekengi L.
Solanaceae
Wet woods and hedges. Northern Italy: C; Central and southern Italy up to Cilento and Basilicata (Pomarico), Calabria, northern Sicily, Sardinia: R. (0-1000 m). Temperate Eurasian.

2n=24
Red: calyx, berries' juice
PAL – 80316

Physocarpus opulifolius (L.) Maxim.
Rosaceae
Cultivated in parks for ornamentation and observed sub-spontaneous in Piedmont and Lombardy. (0-500 m). N-American.
2n=18
Red: leafless stem
VHLVB - 10 0463624

Phytolacca americana L.
Phytolaccaceae
Vegetable gardens, uncultivated. Cultivated for the berries used to color the wine, it expands vigorously thanks to the roots: always near the inhabited areas. In the whole territory: C. (0-350). N-American.
2n=36
Red: berries' juice
PAL – 59117

Pistacia lentiscus L.
Anacardiaceae
It is one of the most common and representative shrubs of the Oleo-ceratonion, often in association with the olive and myrtle. Its presence in the Mediterranean maquis and garrigue is more sporadic. Quite frequent also in the bushy pastures and in the more degraded residual areas of the maquis. Peninsula, Sicily and Sardinia: CC. (0-600 m). Steno-Mediterranean.
2n=30
Red: leaves
PAL – 73415

Pistacia terebinthus L.
Anacardiaceae
On the slopes and in the cracks of limestone and arid cliffs, inside thermophilic woods. Peninsula, Sicily and Sardinia: C. (0-1000 m). Euri-Mediterranean.
2n=ignote
PAL – 68053

Picea abies (L.) H.Karst.
Pinaceae
The main component of the sub-alpine coniferous forest. Alps: CC; Northern and Tuscan Apennines: RR. (0-2200 m). Eurosiberian.
2n=24
Brown: top of the young branches
Black: soot
H.PAOL – 2209

Pinguicula vulgaris L.
Lentibulariaceae
Wet meadows, marshes, springs (generally acidophilous). Alps, from Carnia to Liguria, Appennino to Monti della Laga: R. (400-2350 m). Circumboreal.
2n=64

Red: leaves’ juice
PAL-GR – 64193_GR

Pinus halepensis Mill.
Pinaceae
Arid slopes, spots and garrigues, especially coastal and on limestone. In the whole Peninsula, Sicily and Sardinia and smaller Islands: C; cultivated and sub-spontaneous on the Triestina and Garda coasts. (0-800 m). Steno-Mediterranean.
2n=24
Red: pulverized bark, cones
Yellow: leaves
PAL – 71800

Pinus pinea L.
Pinaceae
Dunes, scrub, dry slopes. Along the coasts of the Peninsula, Sicily, Sardinia and smaller Islands, northwards as far as Ravenna and Chioggia: C; less widespread inside, almost everywhere cultivated (indigenous doubt plant). (0-800 m). Euri-Mediterranean.
2n=24
Brown: bark
PAL – 21232

Pinus sylvestris L.
Pinaceae
Woods of the mountain belt. Arid centroalpine valleys from Tarvisio to Val Susa: C; also in the upper Lombard Plain, on the Asti side of the Collina di Torino, and on the northern Apennines from the Piacentino to the Modenese; grown in the rest of the Alps, Peninsula, and Sardinia. (100-1800 m). Eurasian orophyte.
2n=24
Red: wood
Yellow: young branches’ bark
Black: soot
PAL-GR – 64921_GR

Plantago lanceolata L.
Plantaginaceae
Uncultivated, along the streets, fields, vineyards, generally synanthropic. In the whole territory: CC. (0-2000 m). Cosmopolitan.
2n=12, 24
Green: leaves
PAL – 78530

Platanus acerifolia (Aiton) Willd.
Platanaceae
Parks, along the streets. Cultivated and feral throughout the territory: CC in Padania and in the other irrigated plains. (0-800 m). Euri-Mediterranean.
2n=42
Red: wood
Yellow: branches’ bark
PAL – 107890

Platycladus orientalis (L.) Franco

Cupressaceae
Native to northern China and Japan, cultivated for ornamentation and in the south for forestry purposes, run wild in Piedmont. (0-800 m). E-Asian.
2n=22
Yellow: branches with leaves
PAL – 21249

Plumbago europaea L.
Plumbaginaceae
Uncultivated, edges of streets, walls. Liguria, Peninsula, Sicily, Sardinia: R. (0-800 m). Steno-Mediterranean.
2n=12
Yellow: grass
PAL – 90467

Polygonum aviculare L.
Polygonaceae
Uncultivated and wet depressions. In the whole territory: Peninsula (on the reliefs) and along the coasts: CC; alpine valleys: R; not very common in Padania and in general in the alluvial plains where it is generally replaced by *P. arenastrum*. (0-850 m). Cosmopolitan.
2n=60
Yellow: whole plant
Blue: leaves
PAL – 69259

Polypodium vulgare L.
Polypodiaceae
Cliffs, walls, bark of trees. Alps, Northern Apennines: C; Friulano-Veneto and Padana plains: from RR to R; central and southern regions and islands: R is available at altitudes gradually, progressing towards the south; uncertain for Calabria and Sicily. (20-2850 m).
2n=148
Red: roots
PAL – 9455

Populus alba L.
Salicaceae
Damp or flooded stations along rivers and on lakes. In the whole territory: C. (0-1000 m). Paleo-tempered.
2n=38 (57)
Yellow: young branches
PAL – 64793

Populus nigra L.
Salicaceae
Spontaneous along rivers and lakes. In the whole territory: C; cultivated for ornament. (0-1200 m). Paleo-tempered.
2n=38, 57
Yellow: bark
PAL – 70770

Populus tremula L.
Salicaceae

Mountain woods, especially wet, more rarely up to the plains. In the whole territory: C. (0-2000 m) Euro-Siberian.
2n= (19) 38 (57)
Yellow: bark
PAL – 83964

Potentilla anserina L.
Rosaceae
Trampled soils rich in soluble salts (on the subalophyte coasts, inside nitrophilous). Alps (from Carnia to Val d'Aosta, especially in valleys with a continental climate), Padania, Lazio, Avellino: C. (0-1800 m). Sub-cosmopolitan.
2n=28 (42)
Yellow: leaves
PAL – 23689

Potentilla erecta (L.) Raeusch.
Rosaceae
Meadows, heaths, and woods (acidophilous). In the whole territory: C. (0-2400 m). Eurasian.
2n=28
Red: roots
H.PAOL - 1850

Prunella vulgaris L.
Lamiaceae
Meadows, pastures, sepis, scrublands. In the whole territory: CC (0-2000 m). Circumboreal.
2n=28
Green: flowery plant
PAL – 15002

Prunus armeniaca L.
Rosaceae
Commonly cultivated. In the whole territory; shows no tendency to run wild. (0-1000 m). Central Asia.
2n=16
Yellow: young branches
PAL – 26405

Prunus avium (L.) L.
Rosaceae
Oaks and chestnut woods, often sub-spontaneous. Probably indigenous in broad-leaved woods on sub-acid soil: cultivated on a large scale throughout the territory (in Sicily only as a cultivated species) C. (0-1500 m). European-Caucasian.
2n=16
Yellow: dry wood
PAL-GR – 62403_GR

Prunus cerasus L.
Rosaceae
Often cultivated and wild. Throughout the territory, but probably only as an introduced plant. (0-800 m). Pontic.
2n=32
Red: fruit

PAL – 107891

Prunus domestica L.
Rosaceae
Commonly cultivated and sub-spontaneous (or spontaneous). In the whole territory. (0-1000 m). Uncertain origin.
2n=48
Yellow: bark, wood, dry fruit
Brown: root
PAL – 26409

Prunus dulcis (Mill.) D.A. Webb
Rosaceae
Cultivated. In whole territory, except Calabria: C. (0-1000 m). S-Mediterranean
2n=ignote
H.PAOL - 1918

Prunus laurocerasus L.
Rosaceae
Cultivated for ornament. Across the territory, sub-spontaneous in areas with a Mediterranean climate; wild in Insubria, in the coastal pine forests in Tuscany, Castelli Romani and in Campania: R. (0-300 m). W-Asian.
2n=144, 170-180
Red: branches, leaves
H.PAOL – 1915

Prunus mahaleb L.
Rosaceae
Sub-Mediterranean thermophilic bush (especially in the Quercetum pubescentis), coppices, hedges. Alps, Peninsula, Sicily: C to R. (0-800, in Sicily up to 1900 m). S-European-Pontic.
2n=16
Yellow: young soft branches
PAL – 84609

Prunus persica (L.) Stokes
Rosaceae
Cultivated on a large scale and grown in shrubbery and deciduous coppice. In the whole territory: C. (0-600 m). East Asia.
2n=16
Red: young branches, kernel
PAL – 41869

Prunus spinosa L.
Rosaceae
Edges of the woods and paths, in sunny places. In whole territory: C. (0-1500 m). Eurasian.
2n=32, (40, 48)
Red: young branches
PAL – 26500

Pteridium aquilinum (L.) Kuhn subsp. *aquilinum*
Dennstaedtiaceae

Not thick woods, heaths, grassy slopes and terraces, uncultivated fields, on acid substrate also of calcareous origin. In the whole territory: from C to CC. (0-2100 m). Cosmopolitan.
2n=104
Grey: fresh roots
PAL – 69118

Punica granatum L.
Lythraceae
Cultivated for ornamentation or fruit and grown in the gardens. In the whole territory, within the area of the vine. (0-800 m). SW-Asian.
2n=16
Yellow: fruits' peel
Black: epicarp together with *Dittrichia viscosa* and *Rhus coriaria*
PAL – 62397

Pulsatilla montana (Hoppe) Rchb.
Ranunculaceae
Pre-Alpine valleys from Carnia to Piedmont, Friuli and northern Apennines: RR. (200-2400 m). SE-European.
2n=16
PAL-GR – 100168_GR

Pyracantha coccinea M. J. Roemer
Rosaceae
Evergreen woods, holm oaks, hedges. Frequently cultivated and naturalized, sometimes also included in natural vegetation; Peninsular Italy and Liguria, Sardinia: C; along the Adriatic it advances on ancient dunes consolidated up to Mesola and Rosolina; also in the urban area and alpine valleys. (0-900 m). W-Asian.
2n=34
Red: branches
PAL – 22955

Pyrus communis L.
Rosaceae
Broadly cultivated. In the whole territory, from the plains to the middle mountains. (0-1000 m).
2n=34 (51, 68)
Yellow: bark, wood
Brown: dry fruit
PAL – 6729

Quercus cerris L.
Fagaceae
Forests, especially on sub-acid soil with deep water stagnation. In the whole territory, excluding Sardinia and small islands; Padania: R is perhaps introduced, in the rest: C. (100-800, rarely 0-1500 m). N-Euri-Mediterranean.
2n=24
Brown: bark, young stem
Black: galls
PAL – 70468

Quercus coccifera L.
Fagaceae

Mediterranean scrub of the Oleo-Ceratonion type and in the garrigues linked to the degradation of the evergreen Mediterranean forest. Peninsula except Campania, Sardinia: RR; Sicily: C. (0-500 m).Steno-Mediterranean
2n=24
Black: galls
PAL – 55807

Quercus congesta C.Presl
Fagaceae
Mountain forest. Sicily, Sardinia, Calabria and Basilicata: C. (400-1400 m). NW-Mediterranean.
2n=24
Black: galls
PAL – 55878

Quercus ilex L.
Fagaceae
Soils not too humid, with good drainage. Whole territory, except Val d'Aosta: C. (0-1800 m). Steno-Mediterranean
2n=24
Black: galls
PAL – 55841

Quercus ithaburensis subsp. *macrolepis* (Kotschy) Hedge & Yalt.
Fagaceae
At the edge of the fields; arid forests. Perhaps in ancient times introduced from the Greek coasts. Peninsula Salentina, Matera: RR and to be protected. (0-200 m). East-Mediterranean.
2n=24
Brown: bark, branches, exudate
Black: galls, acorn' top
PAL-GR – 51858_GR

Quercus petraea (Matt.) Liebl.
Fagaceae
Acid or sub-acid soils, northern and central Italy, a distinct subspecies in southern Calabria and Sicily.
2n=24
Brown: bark, branches, exudate
Black: galls, acorn' top
PAL – 69120

Quercus pubescens Willd.
Fagaceae
Limestone slopes facing south. In whole territory: C. (100-1200 m). S-European.
2n=24
Black: galls
PAL – 56188

Quercus robur L.
Fagaceae
Fertile and deep soils in northern Italy and the Peninsula up to the Neapolitan. (0-800 m). European-Caucasian.
2n=24

Brown: bark, branches, exudate
Black: galls, acorn' top
PAL – 88948

Quercus suber L.
Fagaceae
Evergreen stains and woods (silica). C on the western coasts from Nizzano to Calabria, Sicily, Sardinia, elsewhere R (0-700 m). W-Mediterranean (Euri-).
2n=24
Black: charred bark
PAL – 56160

Quercus virgiliana (Ten.) Ten.
Fagaceae
In quite dry woods, on weakly acid soils, with optimum in the sub-Mediterranean zone. In the whole territory (200 -1200 m). SE-European
2n=24
Gray: bark
PAL – 70806

Ranunculus acris L.
Ranunculaceae
Meadows and uncultivated. Northern Italy: CC; Peninsula: R (especially on the reliefs); Sardinia (naturalized); missing in Sicily. (0-1600, maximum 2530 m). Sub-cosmopolitan.
2n=14
Yellow: flowery plant
PAL – 46208

Raphanus sativus L.
Brassicaceae
Plant of unknown origin, perhaps fixed hybrid, commonly cultivated and sub-spontaneous. In the whole territory: C. (0-1000 m).
2n=18 (36)
Purple: epidermis of the root
PAL – 5802

Reseda lutea L.
Resedaceae
In whole territory: C. (0-1600 m). Subcosmopolitan
2n=24, 48
PAL – 72617

Reseda luteola L.
Resedaceae
Riverbeds, stony, railways, walls. In the whole territory (excluding most of Padania): R. (0-1300 m). Circumboreal.
2n=24, 26, 28
Yellow: whole plant
PAL – 72641

Rhamnus alaternus L.
Rhamnaceae

Typical of holm oak and evergreen bush. Liguria, Peninsula, Sicily and Sardinia: C; to the north up to the Romagnolo Apennines, Bolognese, Reggiano, Garfagnana and Lunigiana; also in Trieste (indigenous doubt), Groppa a Semonzo; cultivated by ornament and naturalized on Lake Garda, Lake Como and Ivrea. (0-700 m). Steno-Mediterranean.
2n=24
Yellow: young branches, leaves
PAL – 84809

Rhamnus cathartica L.
Rhamnaceae
Thermophilic woods, bushes. Alps, Liguria, Peninsula (especially the eastern side) and Sicily (Madonie): C. (0-800, rarely 1400 m). S-European-Pontic.
2n=24
Red: overripe berries
Yellow: bark, green berries
Green: roots, ripe berries' juice
PAL-GR – 102182_GR

Rhamnus infectoria L.
Rhamnaceae
Prevalent on the Apennines, exclusive at least on the Auruncis, southern Apennines, and Sicily (Madonie, Nebrodi, Sicani).
2n=24
Yellow: green berries' peel
Green: ripe berries
H.PAOL – 4272

Rhus coriaria L.
Anacardiaceae
Uncultivated arid, often as a relic of ancient crops (plant rich in tannins). Liguria, Peninsula (missing in Umbria): R is disappearing; Sicily: C; naturalized also in the Trieste, Veronese, and province of Turin. (0-800 m). S-Mediterranean.
2n= ignote
Red: roots' bark
Yellow: bark
Brown: leaves
PAL – 73607

Rhus typhina L.
Anacardiaceae
Run wild in the uncultivated. Padania, southern slopes of the Alps, hills of Emilia, Cornero, Basilicata: R. (0-500 m). N-American.
2n=30
Yellow: bark
VHLVB - 10 0355409

Ribes rubrum L.
Grossulariaceae
Cultivated and spontaneous in cool places and in mountainous and valley bottom humid forests. Become spontaneous in the Alps, cultivated in the Apennines. (0-1500 m). W-European.
2n=16
Red: berries

Yellow: branches
VHLVB - 10 0340750

Ribes uva-crispa L.
Grossulariaceae
Mountain species of beech, fir, and larch woods, from where it sometimes descends into the lower parts of the valleys. Frequently cultivated. Alps and Apennines, up to Abruzzo: R; also in Sicily, on the slopes of Pizzo Carbonara (Madonie). (800-1500 m). Eurasian.
2n=16
Yellow: branches, leaves
PAL-GR – 50636_GR

Ricinus communis L.
Euphorbiaceae
Cultivated for oil and run wild. Veneto, Lazio, Campania, Calabria, Sicily, Sardinia: R; elsewhere sporadic and inconstant. (0-300 m). Paleotropical.
2n=20
Yellow: leaves, spike
PAL – 51694

Robinia hispida L.
Fabaceae
Adventitia in the Marche. E-N-American.
2n=30
Yellow: dry branches, bark
VHLVB - 13659-010

Robinia pseudoacacia L.
Fabaceae
Escarpments, uncultivated, hedges. Introduced in the seventeenth century and now completely spontaneous throughout the territory, in abandoned places, hedges, embankments, but always synanthropic. (0-1000 m). N-American.
2n=20, 22
Yellow: Dry wood, young branches
PAL – 72752

Rosa canina L.
Rosaceae
Woodlands, hedges, shrubberies. In all regions: C. (0-1500 m). Paleo-tempered.
2n=35
Yellow: roots, wood
PAL – 25238

Rosa pendulina L.
Rosaceae
Woodland edges, cliffs. Northern and central Italy, in all regions: C; also in Campania, it is lacking in the rest of southern Italy and the Islands. (600-1800 m). S-European orophyte.
2n=28
Yellow: young branches
PAL-GR – 54564_GR

Rosa rubiginosa L.

Rosaceae
Woodlands, shrubberies. Trieste Karst, Alps, from Trentino Alto Adige to the Maritime Alps: Widespread, also in Tuscany, Abruzzo, Campania, Calabria. (600-1400 m). Eurasian.
2n=35
Yellow: young branches
PAL – 26162

Rosmarinus officinalis L.
Lamiaceae
Stains and garrigues (calcareous). Liguria, Peninsula on the western and southern coasts (on the Adriatic only up to Molise), Sicily, Sardinia and in almost all the smaller Islands: C; also on the west bank of the Garda: RR; throughout the rest of the territory, commonly cultivated and often sub-spontaneous. (0-800 m). Steno-Mediterranean.
2n=24
Yellow: branches, leaves
PAL – 13993

Rubia peregrina L.
Rubiaceae
Evergreen woods (holm oaks) and more rarely deciduous trees, spots, hedges. Liguria, Sicily, Sardinia, smaller islands, Peninsula: C. (0-1000 m). Steno-Mediterranean-Macaronesian.
2n=66
Red: roots
PAL – 59797

Rubia tinctorum L.
Rubiaceae
Woodlands, hedges. Cultivated in the last century for the dyeing of fabrics, rarely naturalized in the sub-Mediterranean area (from the alpine valleys to the Islands), but in the process of disappearance: R. (0-1000 m). W and Central Asian.
2n=66
Red: roots
PAL – 59837

Rubus idaeus L.
Rosaceae
Moist mountain woodlands, on any substrate (200 - 2.000 m). In the whole territory, common in the Northern regions (0-1200 m). Circumboreal-Eurosiberian.
2n=14
Yellow: stem, fruits
PAL – 23155

Rubus odoratus L.
Rosaceae
Introduced as an ornamental plant and frequently run wild. (0-1800 m). N-American.
2n=14
Yellow: dry stem
PAL – 23176

Rumex acetosa L.
Polygonaceae
Mowed lawns, fertilized. In the whole territory: C. (0-2000 m). Sub-cosmopolitan.

2n=14, 15
Red: dry roots
Yellow: green roots
VHLVB - 10 0483410

Rumex acutus L.
Polygonaceae
Vegetation of high grasses on the edge of the humid woods, in the flooded riverbeds, near marshes, ditches etc. In the whole territory (missing in Val d'Aosta): C. (0-1300 m). Circumboreal-S-African.
2n=20
Yellow: roots
VHLVB - 07035 -010

Rumex alpinus L.
Polygonaceae
Around the huts, on meadows with fertilizers and manure. Alps from the Carnie to the Maritime: C; Apennines from Liguria to Pollino: widespread. (1600-2300 m, it rarely goes down to 800 m). Euroamerican.
2n=20
Yellow: roots
PAL – 89768

Rumex aquaticus L.
Polygonaceae
Slow waters, swamps. Safe indications only for southern Tyrol and Tuscany: RR; to be eliminated from the Trieste Karst and to be verified in Molise and Lazio; elsewhere reported due to confusion. (0-900 m). Eurosiberian.
2n=140
Yellow: fresh roots
PAL – 89769

Rumex maritimus L.
Polygonaceae
Wet soils, sometimes sub-salts, sometimes confused with *R. palustris*. Veneto-Friulano coast, Sicily, Sardinia: RR. Eurasian. Observed in America.
2n=40
Yellow: roots
PAL – 90657

Rumex sanguineus L.
Polygonaceae
Wet deciduous woods, rarely also beech woods, oak woods, cork woods. In the whole territory (missing in Veneto, Trentino Alto Adige, and Emilia-Romagna): R. (0-1200 m). Circumboreal.
2n=20
Yellow: grass
PAL – 71235

Ruscus aculeatus L.
Asparagaceae
Holm oak, thermophilous deciduous forests. In the whole territory (however it is missing in part of Padania): C. (0-600, in the South 0-1200 m). Euri-Mediterranean.
2n=40

Yellow: stem, leaves
PAL – 61876

Ruta graveolens L.
Rutaceae
Grassy slopes and cliffs (calcareous). Northern Italy and the Peninsula (especially on the eastern side): C; but mostly cultivated and wild. (0-1100 m). Euri-Mediterranean.
2n=72
Green: leaves, stem
PAL-GR – 102164_GR

Salix alba L.
Salicaceae
Riveras stations and wet places. In the whole territory: C is frequently cultivated. (0-1200, sometimes 1600 m). Paleo-tempered.
2n=76
Red: debarked wood
Yellow: bark, branches
PAL – 65028

Salix alba subsp. *vitellina* (L.) Schübl. & Martens
Salicaceae
Cultivated and wild.
2n=76
Yellow: branches
PAL – 88316

Salix atrocinerea Brot.
Salicaceae
Freshly disturbed land, on beaches near the sea and on islands. Sandy or gravel shores of rivers, streams and ponds, meadows, valleys and hedgerows with some soil moisture. Sardinia and Tuscany: R. (0-1000 m). Euri-W-Mediterranean.
2n=74
Yellow: leaves
PAL – 102252

Salix caprea L.
Salicaceae
Edge of woods, escarpments, and wet areas. In the whole territory; Alps: C; Padania and the Apennines: R; Sicily and Corsica: RR; missing in Sardinia. (0-1800 m). Eurasian.
2n=38, 76
Red: debarked wood
Yellow: bark, branches, wood
PAL – 83447

Salix pentandra L.
Salicaceae
Marshes and peaty soils subject to temporary submersions. Alps, from Alto Adige to Piedmont; also, reported for the Tuscan-Emilian Apennines, Lazio and Molise: RR; Sardinia: R. (500-1900 m). Eurosiberian.
2n=76
Yellow: leaves

PAL-GR – 54571_GR

Salix purpurea L.
Salicaceae
Riverbeds and banks of water courses. In the whole territory including the Islands: C. (0-1800 m). Temperate Eurasian.
2n=38
Yellow: bark
PAL – 65114

Salvia officinalis L.
Lamiaceae
Arid cliffs and stony (calcareous). Trieste Karst, southern Lazio on the Ausoni, southern Italy, Sardinia (Monte di Oliena): R; also in Abruzzo (Fucino) and sub-spontaneous in northern and central Italy (0-300 m). E-Steno-Mediterranean.
2n=14
Yellow: stump, woody stem
PAL – 69437

Sambucus ebulus L.
Adoxaceae
Uncultivated, edges of fields and streets. In the whole territory: Liguria, Peninsula, and Islands: C; Alps and Padania: R. (0-1300 m). Euri-Mediterranean.
2n=32
Purple: ripe berries
PAL – 69440

Sambucus nigra L.
Adoxaceae
Wet woods, clearings, coppices, hedges. In the whole territory: C. (0-1400 m). European-Caucasian.
2n=36
Yellow: bark, branches, dry flowers
Purple: ripe berries
Brown: wood
Black: seed
PAL – 68508

Sambucus racemosa L.
Adoxaceae
Glade, woodland paths, especially in beech woods. Alps from Carnia to Liguria and northern Apennines: C; Umbria-Marche and Abruzzo Apennines at the source of the Melfa: RR. (900-2000 m). S-European orophyte.
2n=36
Yellow: young leafy branches
PAL – 96208

Sanguisorba officinalis L.
Rosaceae
Marshy meadows, low bogs. Alps and Padania (here almost disappeared following reclamation): C; Northern Apennines, central Italy, Pollino and Sila: R. (0-2000 m). Circumboreal.
2n=28, 56

Brown: whole plant
PAL – 24808

Saponaria officinalis L.
Caryophyllaceae
Uncultivated wet along the waterways. Largely naturalized in the gardens, along the streets, on the ruins and abandoned lands; throughout the territory: C. (0-1000 m). Eurosiberian.
2n=28
Red: pollen
PAL – 69441

*Satureja hortensi*s L.
Lamiaceae
Uncultivated arid, walls (calcareous). Northern and central Italy, Sicily: R: probably only cultivated and sub-spontaneous. (0-1300 m). Euri-Mediterranean.
2n=24, 45, 48
Green: flowery stem
PAL – 16326

Scandix pecten-veneris L.
Apiaceae
Grassy environments and cereal fields. In the whole national territory: from R to C. (0-1200, rarely 1900 m). Sub-cosmopolitan.
2n=26
Yellow: grass, seeds
PAL – 90390

Scleranthus perennis L.
Caryophyllaceae
Arid stations. Northern and central Italy, Sicily: R. (600-1500, rarely 100-2100 m). Eurosiberian.
2n=22, 44
Yellow: roots
PAL – 83977

Scorzonera hispanica L.
Asteraceae
Rock slopes, steppe meadows. In the whole territory (excluding Lombardy, Calabria, and Islands): R. (300-1900 m). SE-European-S-Siberian.
2n=14
Grey: roots
PAL_GR – 64448_GR

Scrophularia nodosa L.
Scrophulariaceae
Wet woods, ravines, shores. Alps and Apennines up to Basilicata: C; Padania: R; also in Sardinia, Elba, and Ischia. (0-1800 m). Circumboreal.
2n=36
Yellow: stem, leaves
PAL – 43465

Selaginella denticulata (L.) Spring
Selaginellaceae

Grassy slopes, mossy terraces, sloping brook margins, cliffs, walls, from sunny to shaded stations. Coasts and Tyrrhenian and Ionian Islands: from CC to C; inland areas of central and southern regions, Adriatic coasts from the Marche to the south: from RR to R. (0-1500 m). Steno-Mediterranean-Macaronesian.
2n=18
Yellow: whole plant
PAL – 6062

Serratula tinctoria L.
Asteraceae
Woods, meadows, marshes. In the whole territory, not indicated in Puglia, Calabria and Sardinia. Northern Italy, the Tuscan-Emilian Apennines and the Marches: C; in the rest of the Peninsula and Sicily: R. (0-1600 m). Euro-Siberian.
2n=22
Yellow: stem, dry leaves, flower
PAL – 30850

Seseli libanotis (L.) W.D.J. Koch subsp. *libanotis*
Apiaceae
Pastures, dry meadows, and rocky environments (calcareous). Alps and central-southern Apennines (with lacunae): R. (500-2500 m). Pontic-Central European.
2n=22
Yellow: fresh plant
PAL – 25065

Silene vulgaris (Moench) Garcke S. subsp. *vulgaris*
Caryophyllaceae
In fields and meadows, especially if mown and fertilized, in lowlands and low mountains, often even ruderal-nitrophilous. In the whole territory (excluding the most arid areas): CC, but mostly synanthropic. (0-1500, rarely 2400 m). Sub-cosmopolitan.
2n=24
Green: leaves, flowery stem
PAL – 85323

Sisymbrium officinale (L.) Scop.
Brassicaceae
Ruins, rubble (synanthropic). In the whole territory: C. (0-1000 m, rarely up to 2400 m). Sub-cosmopolitan.
2n=14
Green: spikes
PAL – 5358

Sium latifolium L.
Apiaceae
Stagnant waters, banks, and marshes. Sporadic in Padania and Peninsula (in different locations disappeared or threatened by environmental alterations): R. (0-600 m). Central European.
2n=12, 20
Grey: leaves, flowery stem
PAL – 23835

Sixalix atropurpurea (L.) W.Greuter & Burdet
Caprifoliaceae

Liguria, Peninsula, Sicily and Sardinia: C. (0-1400 m). Steno-Mediterranean
2n=16
Green: whole plant
PAL – 93722

Smilax aspera L.
Smilacaceae
Spontaneous in the woods and in the maquis. Peninsula, Liguria, Sicily and Sardinia: C. (0-1200 m). Subtropical
2n=32
PAL – 82957

Solanum dulcamara L.
Solanaceae
Wet woods, uncultivated, generally in shady environments. In the whole territory: C. (0-1100, maximum 1450 m). Paleo-tempered.
2n=24 + 2B - 24
Grey: leaves, flowery stem
PAL – 80412

Solanum lycopersicum L.
Solanaceae
Cultivated on a large scale and often sub-spontaneous on rubble, shores, ruins, throughout the territory: C, but does not tend to become naturalized. (0-1500 m). Cultivated species.
2n=24, 48
Yellow: leaves, stem
PAL - 80383c

Solanum nigrum L.
Solanaceae
Uncultivated and ruins; edges of paths and roads. It is easily found in vegetable gardens, vineyards or cornfields. In whole territory: CC. (0-1000 m). Cosmopolitan
2n=12, 24, 36, 72
PAL – 80507

Solanum tuberosum L.
Solanaceae
Commonly cultivated and sub-spontaneous near crops. (0-1500 m). Cultivated species.
2n=24, 36, 48
Yellow: green leaves, flowery stem
PAL – 107198

Solidago canadensis L.
Asteraceae
Marshes, hygrophilous woods (especially poplar groves), uncultivated humid, banks, embankments. Reported in the North (naturalized in Friuli, Veneto, Lombardy, Piedmont, Liguria, Emilia-Romagna; invasive in Trentino and southern Tyrol): C; at the center of cases in Umbria, Marche, and Lazio. No longer found in Tuscany. (0-800 m). Circumboreal.
2n=18
Yellow: stem, leaves, flower
VHLVB - 10 0682460

Solidago virgaurea L.
Asteraceae
Woods, woods, pastures. In the whole territory, excluding Sicily: C (from Padania almost everywhere disappeared, it is generally missing in the area of the holm oak). Circumboreal.
2n=18
Yellow: leaves, flowery stem
PAL – 26896

Sonchus oleraceus L.
Asteraceae
Street edges, crops. In the whole territory: CC. (0-1700 m). Sub-cosmopolitan.
2n=18, 32, 36, 64
Yellow: leaves, flowery stem
PAL – 68545

Sorbus aucuparia L.
Rosaceae
Mountain woods (beech trees, fir trees) and subalpine woods, rhododendron bushes. Alps and Northern Apennines: C; other reliefs of the Peninsula, Sicily, and Sardinia: R. (600-2100 m, rarely up to the plain). European.
2n=34
Red: young branches
PAL – 22813

Sorbus domestica L.
Rosaceae
Sub-Mediterranean woods; also, cultivated for the fruit. In the whole territory, however, in northern Italy: R is often wild. (0-800 m). N-Euri-Mediterranean.
2n=34
Red: young branches
Brown: fruit
PAL – 22824

Sorghum bicolor (L.) Moench
Poaceae
Uncultivated, fields. Mainly cultivated in the Padania and in Tuscany, rarely subspace. (0-500 m). Paleotropical.
2n=20
Purple: seeds
PAL – 93140

Spartium junceum L.
Fabaceae
Bushes in sunny stations. In the whole territory: C. (0-600 m, in the Apennines up to 1200-1400, on Etna up to 2000 m). Euri-Mediterranean.
2n=48
Yellow: young branches
Light green: young branches and flowers
PAL – 68530

Spinacia oleracea L.
Amaranthaceae

Anthropized and ruderal environments (cultivated, sometimes escapes). Veneto, Emilia-Romagna, Tuscany, Marche, Umbria, Abruzzo. (0-1500 m). W-Asian.
2n=12
Green: leaves
PAL – 58446

Stachys sylvatica L.
Lamiaceae
Broad-leaved woods on damp ground, their clearings, and glades. Northern and central Italy: C; Southern Italy and Sicily: R; in the Padania almost everywhere disappeared. (0-1700 m). Eurosiberian.
2n=33
Yellow: grass
PAL – 37006

Staphylea pinnata L.
Staphyleaceae
Broad-leaved thermophilous woods, sunny cliffs. Trieste Karst, Alpine foothills from Friuli to Piedmont, northern slope of the Apennines in Parmigiano, Reggiano, Bolognese, Romagna and Marche, reliefs of Lazio and Campania: R; also in Tuscany, Basilicata, and western Calabria. (0-900 m). SE-European-Pontic.
2n=26
Yellow: leafy branches
PAL-GR – 102176_GR

Stellaria alsine Grimm
Caryophyllaceae
Springs, streams, swamps. Alps, Northern Apennines up to Romagna, Basilicata (Vulture, Muro) and Sila: R. (1000-2000 m, rarely less). Circumboreal.
2n=24, 26
Green: whole plant
PAL-GR – 101712_GR

Succisa pratensis Moench
Dipsacaceae
Wet meadows. Alps: C; in the rest of northern Italy, Tuscany, Lazio, Umbria, Agro Pontino, Abruzzo: R; also on the Sila and perhaps in Basilicata on the Pollino. (0-1600, max 2400 m).
2n=20
Yellow: roots
PAL – 89236

Sulla coronaria L. (Medik.)
Fabaceae
Clay soils also sub-salty. Liguria, the Peninsula (in the north as far as the Via Emilia), Sicily, Sardinia, and the nearby minor islands, but often cultivated and sub-spontaneous; missing from Padania, also cultivated on the Garda, Bergamasco and Novarese hills. (0-1200 m). W.-Mediterranean.
2n=16
Brown: fresh flowers
PAL – 91697

Symphytum officinale L.

Boraginaceae
Wet meadows, riparian woods, often on the banks and along the ditches. Padania and alpine valleys: C; Peninsula in Versilia, Tuscan-Emilian Apennines, Umbria, Lazio, Campania, Basilicata, Sicily (Madonie, Mezzojuso): R. (0-1700 m). European-Caucasian.
2n=24
Black: grass
PAL – 63775

Syringa vulgaris L.
Oleaceae
Woodlands, hedges. Cultivated by ornament and often completely spontaneous in the Prealps from Friuli to Piedmont, Northern Apennines etc. (0-800 m). SE-European orophyte.
2n=46, 47, 48
Yellow: branches
VHLVB - 10 0682460

Tagetes erecta L.
Asteraceae
Man-made environments. Friuli, Veneto, Emilia-Romagna and Lazio. Mexico.
2n=24
Yellow: leaves, flowery stem, fresh flower
VHLVB - 10 0220806

Tamarix africana Poir.
Tamaricaceae
Coastal species, present both in large dune and retro-dune sandy areas and in coastal wetlands. Liguria, Peninsula, Sicily and Sardinia: C. (0-800 m). W-Mediterranean.
2n=12
PAL – 92266

Tamarix gallica L.
Tamaricaceae
Alluvial plains, lagoon systems, channels of waterways carved on predominantly carbonatic substrates, on the banks of rivers. Along the whole coast of the Peninsula and in the Islands, it has been confirmed for Veneto, Liguria, Emilia-Romagna, Tuscany, Lazio, Puglia, Basilicata, Sardinia, and Sicily: C, but located (0-800 m). Area to be specified.
2n=24
Yellow: green branches
PAL – 79176

Tanacetum vulgare L.
Asteraceae
Fallow, banks, roadsides, woods, clearings, pastures. Northern Italy, the Peninsula (along the Apennine ridge) up to Campania: C; Eastern Sicily. (0-3000 m). Eurasian.
2n=18
Yellow: leaves, flowery stem
PAL – 9323

Taxodium distichum (L.) Rich. var. *distichum*
Taxodiaceae

Native to North America, from the Carolina to Mexico (marshes and lake environments), cultivated in the gardens of the Po Valley and Tuscany on the edge of ponds and ponds. (0-300 m). N-American.
2n=22
Yellow: branches
VHLVB - 18 0013606

Taxus baccata L.
Taxaceae
Beech and other broad-leaved woods, preferably calcareous. Alps, Apennines, Sicily and Sardinia: R; commonly cultivated. (300-1900 m). Mediterranean-Mountain.
2n=24
Red: bulge on stem, fruit
PAL – 69018

Thalictrum aquilegiifolium L.
Ranunculaceae
Woods (especially beech woods). Alps and Peninsula (only on the reliefs) up to the Gargano and Sila: C; Padania: R. (50-2400 m). Eurosiberian.
2n=14
Green: leaves, stem
PAL-GR – 100210_GR

Thalictrum flavum L.
Ranunculaceae
Marshes, damp woods, peaty meadows. Area to be specified. (0-900 m). Eurasian.
2n=84
Yellow: roots, leaves, flower
PAL – 48180

Thalictrum minus L.
Ranunculaceae
Arid environments and edges of the woods (sub-Mediterranean oak woods), shrubs and clearings. In whole territory, except Puglia: CC. (0-2000 m). Eurasian
2n=42, 70
PAL – 48413

Thapsia asclepium L.
Apiaceae
Arid and rocky or rocky slopes. Central and southern Italy, Sicily, and Sardinia: R. (0-1200 m). Steno-Mediterranean.
2n=22
Yellow: umbels
PAL – 30040

Thapsia garganica L.
Apiaceae
Dry pastures. Tuscany, Latium and Southern Italy, Sicily, Sardinia (0-1400 m). S-Mediterranean
2n=22
Green: leaves
Yellow: Umbels
PAL – 21767

Thlaspi arvense L.
Brassicaceae
Cereal fields, vegetable gardens, ruins. Alps, Padania, Northern Apennines: C; Peninsula up to the Pollino: R. Absent in Puglia and the Islands. (50-2200 m). W-Asian. (Archaeophyte).
2n=14
Green: green plant, green seeds
PAL – 14457

Thuja occidentalis L.
Cupressaceae
Native to New England and Quebec, cultivated in gardens and sporadically wild (Merano). (0-800 m). N-American.
2n=22
Yellow: young branches
S-LINN - 389.15

Thymus vulgaris L.
Lamiaceae
Garrigues, dry slopes. Val d'Aosta, southern Piemote, Liguria, Emilia-Romagna, Tuscany, Umbria, Lazio, Abruzzo: R; often cultivated and sub-spontaneous in Insubria, Marche, Pugli etc. (0-800 m). Steno-Mediterranean-Western.
2n=28, 30
Green: stem, leaves
PAL – 38112

Tilia × *europaea* L.
Malvaceae
Wet woods, ravines, often with elm, ash, alder, beech. Northern and central Italy, Campania, Basilicata, Calabria (Pollino, Serra San Bruno) and Sicily: R; grown in parks and avenues. (0-1200 m). European-Caucasian.
2n=82
Red: green bark
PAL – 66115

Trifolium pratense L.
Fabaceae
Meadows, pastures, uncultivated, also cultivated as forage. In the whole territory: CC. (0-2600 m). Sub-cosmopolitan.
2n=14
Yellow: grass
Green: flower
PAL – 1998

Trigonella foenum-graecum L.
Fabaceae
Uncultivated. Cultivated as fodder and rarely feral in Veneto, the Peninsula (in the north as far as the Via Emilia), Sicily and Sardinia. (0-800 m). SW-Asian.
2n=16
Yellow: grass
PAL – 595

Triticum aestivum L.
Poaceae
Cultivated everywhere over large areas in many cultivars: CC.
2n=42
Yellow: dry culms
PAL – 82530

Tropaeolum majus L.
Tropaeolaceae
Commonly cultivated for ornamentation and sometimes sub-spontaneous in gardens and in ruderal environments of the South. S-American.
2n=28
Yellow: flower
PAL – 92213

Ulex europaeus L.
Fabaceae
Bushes and sub-Mediterranean spots (silica). Apuan Alps and northern Tuscany: C; Peninsula on the western side from Liguria to Campania, Basilicata, and Calabria: R; penetrates eastwards into the Emilian Apennines up to San Marino, Pesaro and Fano. Reported again in Carnia (reforestation), the province of Bolzano (crops) and in the Como area. (0-1000 m). Sub-Atlantic.
2n=96 (32, 64)
Yellow: fresh flowers
PAL – 91640

Ulmus minor Mill.
Ulmaceae
Woods and uncultivated land. Along the bed of streams and streams. Whole territory: CC. (0-1200 m). European-Caucasic.
2n=28
Red: leaves
PAL – 69186

Umbilicus rupestris (Salisb.) Dandy
Crassulaceae
Shady walls or damp cracks in the rock. Peninsula, Sicily, Sardinia: CC; allochthonous in Lombardia, Veneto and Trentino Alto Adige. (0-1200 m). Steno-Mediterranean.
2n=48
PAL – 69192

Urtica dioica L.
Urticaceae
Abandoned lands, heaps of rubbish, nitrophilous, in the houses or even in the wood clearings. In the whole territory: CC. (0-1800, rarely 2300 m). Sub-cosmopolitan.
2n=52
Yellow: roots
PAL – 69327

Vaccinium myrtillus L.
Ericaceae
Woods, moors, bushes, pastures, always on humified acid soil. Alps: C; Northern and central Apennines up to Abruzzo: R. (1200-2000, rarely 300-2800 m). Circumboreal.

2n=24
Purple: berries
PAL – 97868

Vachellia farnesiana (L.) Wight & Arn.
Fabaceae
Cultivated for ornamentation in parks and gardens, and rarely for greening in southern Italy and Sicily.
2n=26, 52
Yellow: flower
PAL – 89112

Verbascum phlomoides L.
Scrophulariaceae
Uncultivated, hedges, ruins. In the whole territory: C in the Peninsula, in the rest R. (0-1300 m). S-European
2n=32, 34
Yellow: leaves, stem, flower
PAL – 44716

Verbascum thapsus L.
Scrophulariaceae
Uncultivated, hedges, ruins. In the whole territory: C. (0-2000 m). European-Caucasian
2n=(30,34) 36
PAL – 80327

Verbena officinalis L.
Verbenaceae
On the roadside, uncultivated trampled (synanthropic). In the whole territory: CC. (0-1200 m). Cosmopolitan.
2n=14
Yellow: flowery stem
PAL – 64982

Veronica chamaedrys L.
Plantaginaceae
Nitrate-rich environments in the woods, bushes, meadows along the paths. Settentironal Italy (especially in the Alps): C; Peninsula on the reliefs up to Calabria, progressively R. (0-2200 m). Euro-Siberian.
2n=16, 32, 36
Yellow: flowery plant
PAL – 89316

Veronica hederifolia L.
Plantaginaceae
Uncultivated, fields, gardens, roadsides, ruins, mountain forests. In the whole territory: C. (0-1800 m). Eurasian.
2n=54
Yellow: flowery plant
PAL – 43952

Veronica officinalis L.

Plantaginaceae
Woods, coppices, glades, moors, on acid soils. In the whole territory; Alps: C, in the rest R. (0-2000 m). Eurasian-Mountain. (-American).
2n=36 (34, 38)
Green: dry plant
PAL – 69042

Veronica serpyllifolia L.
Plantaginaceae
Sub-humid places, meadows, pastures, roadsides, and consortia of high grasses. In the Alps: C, in the rest of the territory: R. (0-2500 m). Sub-cosmopolitan.
2n=14
Yellow: flowery plant
PAL – 44029

Viburnum lantana L.
Adoxaceae
Thermophilous deciduous woods (especially Quercetum pubescentis). Alps and hill systems in front of the northern Apennines on the Emilian side: C; Padania, Alps Apuane, Apennine ridge up to Abruzzo: also in northern Campania and Avellino. (0-1000 m). S-European.
2n=18
Black: ripe berries
PAL – 102043

Viburnum opulus L.
Adoxaceae
Moorish woodlands, poplars, hedges. Alps, Padania, Emilian Apennines: R; also in northern Tuscany (near Pisa, Apennines Pistoiese, Apennines Romagnolo at Sasso di Simone), Umbria in Gubbio, Paludi Pontine, Abruzzo, and Basilicata (Pignola, Monte Arioso, Val d'Agri); still indicated for Campania and Salento Peninsula, but to be verified. (0-1100 m). Eurasian temperate.
2n=18
Red: green branches, ripe berries
H.PAOL – 2684

Viburnum tinus L.
Adoxaceae
Holm oaks, evergreen forests, hedges. Liguria, Peninsula (north to Cornero and Furlo, Perugia, Trasimeno, Siena, Lucca, Sarzana), Sicily, Sardinia, and smaller islands, also on the coast between Trieste and Duino: C; farther north (shores of Lake Garda, Insubria, Romagna, Florence etc.) cultivated and wild. (0-800 m). Steno-Mediterranean.
2n= ignote
Red: green branches
PAL – 59858

Vinca major L.
Apocynaceae
Woods, hedges, parks. In the whole territory (excluding Sardinia), but often wild. (0-800 m). Euri-Mediterranean.
2n=92
Yellow: stem, leaves
PAL – 63511

Vincetoxicum hirundinaria Medik. s.l. subsp. *hirundinaria*
Apocynaceae
Craggy sunny bushes, edges of woods, hedges. In the whole territory (missing in Sicily): C. (0-1000, rarely 1700-1900 m). Eurasian.
2n=22
Green: leaves, stem
PAL – 93446

Viola odorata L.
Violaceae
Woodland margins, hedges, grassy and wild places; often grown in gardens and run wild. In the whole territory: C. (0-1200 m). Euri-Mediterranean.
2n=20
Blue: flower
PAL – 73331

Viola tricolor L.
Violaceae
Fields, pastures. Distribution in Italy is not well known; Northern Italy: Widespread; Central and southern Italy up to Basilicata; Sardinia only in the mountains; missing in Sicily. (0-2100 m). Eurasian.
2n=26
Yellow: grass
PAL – 90675

Viscum album L.
Santalaceae
Hemiparasite on various trees and shrubs. (0-1200 m). Eurasian.
2n=20
Yellow: branches, leaves
PAL – 80366

Vitex agnus-castus L.
Lamiaceae
River beds, streams, wet lowlands between the dunes. Liguria, Tuscan and Lazio coasts, Abruzzo at the mouth of the Sangro, southern Italy, Sicily, Sardinia, and smaller islands: R; in the rest of the territory often cultivated and sub-spontaneous. (0-500 m). Steno-Mediterranean-Turanian.
2n= ignote
Green: young branches
PAL – 65229

Vitis vinifera L.
Vitaceae
Cultivated in whole territory. (0-1000 m).
2n=38
PAL – 107828

Xanthium strumarium L.
Asteraceae

Ruins, rubble, dry uncultivated. In the whole territory, in many areas very rare and in regression, not reported in Val d'Aosta and Marche (considered as non-native in Basilicata; dubious in Umbria): R. (0-300 m). Cosmopolitan.
2n=(34) 36
Red: young branches
Yellow: grass, fruits, seeds
PAL – 8921
