# Supplementary material for: Ethnobotany of dye plants in Southern Italy, Mediterranean Basin: floristic catalog and two centuries of analysis of traditional botanical knowledge heritage
Source: J Ethnobiol Ethnomed. 2020 Jun 3;16:31. doi: 10.1186/s13002-020-00384-2 (PMC7268309; doi:10.1186/s13002-020-00384-2)
Supplement: Supplementary file 2 — Additional file 2: Supplementary Table 1. Dye plants catalog, sorted by APG IV criteria. [file 13002_2020_384_MOESM2_ESM.pdf]

Table 1 - Dye plants catalogue, sorted by APG IV criteria.

Family and Botanical Name		Part/s used	Color/s	Pigment/s	References
**Lycopodiaceae**					
	Diphasiastrum alpinum (L.) J. Holub	Whole plant	Yellow	Carotenoids	Briganti, 1842
	Diphasiastrum zeilleri (Rouy) J. Holub	Whole plant	Yellow	Carotenoids	Briganti, 1842
	Lycopodium clavatum L.	Whole plant	Yellow	Carotenoids	Ucria, 1789<br>Briganti, 1842
**Selaginellaceae**					
	Selaginella denticulata (L.) Spring	Whole plant	Yellow	Carotenoids	Briganti, 1842
**Polypodiaceae**					
	Polypodium vulgare L.	Roots	Red	PAHs, Carotenoids, Anthocyanins	Briganti, 1842
**Pinaceae**					
	Abies alba Mill.	Young branches<br>Soot	Brown<br>Black	Tannins<br>Anthocyanins, Tannins, PAHs	Briganti, 1842
	Picea abies (L.) H.Karst.	Top of the young branches<br>Soot	Brown<br>Black	Tannins<br>Anthocyanins, Tannins, PAHs	Briganti, 1842<br>Kakhia, 2015
	Pinus halepensis Mill.	Pulverized bark<br>Cones<br>Leaves	Red<br><br>Yellow	PAHs, Carotenoids, Anthocyanins<br>Carotenoids	Briganti, 1842<br>Fenaroli, 1967<br>Guarrera, 2006(a)<br>Guarrera, 2006(b)<br>Kakhia, 2015
	Pinus pinea L.	Bark	Brown	Tannins	Briganti, 1842<br>Guarrera, 2006(b)<br>Kakhia, 2015
	Pinus sylvestris L.	Wood<br><br>Bark from young branches<br>Soot	Red<br><br>Yellow<br>Black	PAHs, Carotenoids, Anthocyanins<br>Carotenoids<br>Anthocyanins, Tannins, PAHs	Briganti, 1842<br>Guarrera, 2006(b)<br>Kakhia, 2015
**Taxodiaceae**					
	Taxodium distichum (L.) Rich. var. *distichum*	Branches	Yellow	Carotenoids	Briganti, 1842

**Cupressaceae**					
	Cupressus sempervirens L.	Branches	Yellow	Carotenoids	Briganti, 1842
	Thuja occidentalis L.	Young branches	Yellow	Carotenoids	Briganti, 1842
	Platycladus orientalis (L.) Franco	Branches with leaves	Yellow	Carotenoids	Briganti, 1842
**Taxaceae**					
	Taxus baccata L.	Bulge on the stem<br>Fruit	Red	PAHs, Carotenoids,<br>Anthocyanins	Briganti, 1842<br>Guarrera, 2006(b)
**Magnoliaceae**					
	Liriodendron tulipifera L.	Young branches<br>Leaves	Yellow	Carotenoids	Briganti, 1842
**Lauraceae**					
	Laurus nobilis L.	Fresh leaves	Green		Briganti, 1842<br>Guarrera, 2006(b)
**Aristolochiaceae**					
	Asarum europaeum L.	Whole plant	Brown	Tannins	Briganti, 1842
	Aristolochia clematitis L.	Leaves<br>Stem<br>Flower	Yellow	Carotenoids	Briganti, 1842
**Araceae**					
	Arum maculatum L.	Leaves	Yellow	Carotenoids	Briganti, 1842
**Dioscoreaceae**					
	Dioscorea communis (L.) Caddick & Wilkin	Berries	Yellow	Carotenoids	Briganti, 1842
**Colchicaceae**					
	Colchicum autumnale L.	Flower	Yellow	Carotenoids	Briganti, 1842
**Liliaceae**					
	Fritillaria imperialis L.	Whole plant	Yellow	Carotenoids	Briganti, 1842
**Smilacaceae**					
	Smilax aspera L.	Unknown	Unknown	Unknown	Guarrera, 2006(b)
**Asparagaceae**					
	Ruscus aculeatus L.	Stem<br>Leaves	Yellow	Carotenoids	Briganti, 1842
	Convallaria majalis L.	Roots<br>Leaves	Yellow	Carotenoids	Briganti, 1842
	Leopoldia comosa (L.) Parl.	Flower	Blue	Indigotins	Guarrera, 2006(b)
	Muscari negletum Ten.	Flower	Blue	Indigotins	Guarrera, 2006(b)

	Muscari atlanticum Boiss. & Reut.	Flower	Blue	Indigotins	Guarrera, 2006(b)
**Amaryllidaceae**					
	Allium cepa L.	Leaves	Red<br><br>Yellow	PAHs, Carotenoids, Anthocyanins<br>Carotenoids	Briganti, 1842<br><br>Guarrera, 2006(b)
	Narcissus pseudonarcissus L.	Branches<br>Leaves	Yellow	Carotenoids	Briganti, 1842
**Asphodelaceae**					
	Asphodelus aestivus Brot.	Green capsule	Yellow	Carotenoids	Briganti, 1842
	Asphodeline lutea (L.) Rchb.	Green capsule	Yellow	Carotenoids	Briganti, 1842
**Iridaceae**					
	Crocus imperati Ten.	Stigmas	Yellow	Carotenoids	Briganti, 1842<br>Curreli & Loddo, 1983
	Crocus biflorus Mill.	Stigmas	Yellow	Carotenoids	Briganti, 1842<br>Curreli & Loddo, 1983<br>Guarrera, 2006(b)
	Crocus vernus (L.) Hill	Stigmas<br>Flower	Yellow<br>Red	Carotenoids<br>PAHs, Carotenoids, Anthocyanins	Ucria, 1789<br>Briganti, 1842<br>Curreli & Loddo, 1983
	Crocus longiflorus Raf.	Stigmas	Yellow	Carotenoids	Briganti, 1842<br>Curreli & Loddo, 1983
	Crocus thomasii Ten.	Stigmas	Yellow	Carotenoids	Briganti, 1842<br>Curreli & Loddo, 1983
	Crocus sativus L.	Stigmas	Yellow	Carotenoids	Briganti, 1842<br>Curreli & Loddo, 1983<br>Kerry & David, 2001<br>Guarrera, 2006(a)<br>Guarrera, 2006(b)<br>Angelini et al., 2010<br>Caneva et al., 2013<br>Kakhia, 2015<br>Fioretti, 2016
	Iris pseudacorus L.	Roots	Yellow	Carotenoids	Briganti, 1842
	Iris germanica L.	Flower	Green	Clorophylls	Ucria, 1789<br>Briganti, 1842<br>Caneva et al., 2013
**Orchidaceae**					

	Gymnadenia nigra (L.) Rchb.f.	Flower	Purple	Anthocyanins	Briganti, 1842
**Poaceae**					
	Apera spica-venti (L.) P.Beauv.	Spike	Green	Clorophylls	Briganti, 1842
	Phragmites australis (Cav.) Trin. ex Steud. subps. *australis*	Spike	Green	Clorophylls	Ucria, 1789<br>Briganti, 1842
	Bromus secalinus L.	Spike<br>Grass	Green	Clorophylls	Ucria, 1789<br>Briganti, 1842
	Triticum aestivum L.	Dry culms	Yellow	Carotenoids	Briganti, 1842<br>Guarrera, 2006(b)
	Sorghum bicolor (L.) Moench	Seeds	Purple	Anthocyanins	Briganti, 1842
**Commelinaceae**					
	*Commelina communi*s L.	Flower	Blue	Indigoidines	Briganti, 1842
**Cannaceae**					
	Canna indica L.	Seeds	Red	PAHs, Carotenoids, Anthocyanins	Briganti, 1842
**Berberidaceae**					
	Berberis vulgaris L.	Roots<br>Bark<br>Wood	Yellow	Carotenoids	Ucria, 1789<br>Briganti, 1842
**Ranunculaceae**					
	Helleborus foetidus L.	Flowery stem<br>Leaves	Yellow	Carotenoids	Briganti, 1842
	Actaea spicata L.	Juice of berries	Black	Anthocyanins, Tannins, PAHs	Briganti, 1842
	Caltha palustris L.	Flower<br>Grass	Yellow	Carotenoids	Briganti, 1842
	Delphinium consolida L.	Corolla	Blue	Indigoidines	Ucria 1789<br>Briganti, 1842
	Consolida ajacis (L.) Schur	Flowery stem<br>Leaves	Green	Clorophylls	Briganti, 1842
	Anemone nemorosa L.	Leaves	Yellow	Carotenoids	Briganti, 1842
	Clematis vitalba L.	Lianas	Yellow	Carotenoids	Briganti, 1842
	Pulsatilla montana (Hoppe) Rchb.	Unknown	Unknown	Unknown	Guarrera, 2006
	Ranunculus acris L.	Flowery plant	Yellow	Carotenoids	Briganti, 1842
	Thalictrum aquilegiifolium L.	Leaves<br>Stem	Green	Clorophylls	Ucria, 1789<br>Briganti, 1842
	Thalictrum flavum L.	Roots	Yellow	Carotenoids	Ucria, 1789

		Leaves<br>Flower			Briganti, 1842
	Thalictrum minus L.	Unknown	Unknown	Unknown	Guarrera, 2006(b)
**Papaveraceae**					
	Papaver rhoeas L.	Corolla	Red	PAHs, Carotenoids, Anthocyanins	Briganti, 1842<br>Türkmen et al., 2004<br>Guarrera, 2006(a)<br>Guarrera, 2006(b)<br>Maxia et al., 2013<br>Patil et al., 2019
	Papaver somniferum L.	Young soft stem<br>Leaves	Yellow	Carotenoids	Briganti, 1842<br>Patil et al., 2019
	Chelidonium majus L.	Roots<br>Sap<br>Grass	Yellow<br><br>Blue	Carotenoids<br><br>Indigoidines	Briganti, 1842<br>Guarrera, 2006(a)<br>Guarrera, 2006(b)
	Fumaria officinalis L.	Grass	Yellow	Carotenoids	Briganti, 1842<br>Guarrera, 2006(b)
**Platanaceae**					
	Platanus acerifolia (Aiton) Willd.	Wood<br><br>Bark of branches	Red<br><br>Yellow	PAHs, Carotenoids, Anthocyanins<br>Carotenoids	Briganti, 1842
**Loranthaceae**					
	Loranthus europaeus Jacq.	Wood	Red	PAHs, Carotenoids, Anthocyanins	Briganti, 1842
**Santalaceae**					
	Viscum album L.	Branches<br>Leaves	Yellow	Carotenoids	Briganti, 1842
**Cynomoriaceae**					
	Cynomorium coccineum L.	Whole plant	Red	PAHs, Carotenoids, Anthocyanins	Guarrera, 2006(b)
**Paeoniaceae**					
	Paeonia officinalis L.	Flower	Grey	PAHs, Tannins	Briganti, 1842
**Crassulaceae**					
	Umbilicus rupestris (Salisb.) Dandy	Unknown	Unknown	Unknown	Guarrera, 2006(b)
**Grossulariaceae**					
	Ribes rubrum L.	Berries	Red	PAHs, Carotenoids, Anthocyanins	Briganti, 1842

		Branches	Yellow	Carotenoids	
	Ribes uva-crispa L.	Branches<br>Leaves	Yellow	Carotenoids	Briganti, 1842
**Vitaceae**					
	Vitis vinifera L.	Unknown	Unknown	Unknown	Guarrera, 2006(b)
**Tamaricaceae**					
	Tamarix gallica L.	Green branches	Yellow	Carotenoids	Briganti, 1842<br>Guarrera, 2006(b)
	Tamarix africana Poir.	Unknown	Unknown	Unknown	Guarrera, 2006(b)
	Myricaria germanica (L.) Desv. subsp. *germanica*	Fruit	Black	Anthocyanins, Tannins, PAHs	Briganti, 1842
**Plumbaginaceae**					
	Plumbago europaea L.	Grass	Yellow	Carotenoids	Briganti, 1842
	Limonium virgatum (Willd.) Fourr. subsp. *virgatum*	Flower	Blue	Indigoidines	Briganti, 1842<br>Kakhia, 2015
**Polygonaceae**					
	Polygonum aviculare L.	Whole plant<br>Leaves	Yellow<br>Blue	Carotenoids<br>Indigoidines	Briganti, 1842
	Fallopia convolvulus (L.) Á.Löve	Dry plant	Yellow	Carotenoids	Briganti, 1842
	Bistorta officinalis Raf. subsp. *officinalis*	Roots	Red	PAHs, Carotenoids, Anthocyanins	Briganti, 1842
	Persicaria maculosa Gray subsp. *maculosa*	Grass	Yellow	Carotenoids	Ucria, 1789<br>Briganti, 1842
	Persicaria lapathifolia (L.) S. F. Gray subsp. *lapathifolia*	Leaves	Blue	Indigoidines	Briganti, 1842
	Persicaria hydropiper (L.) Delarbre	Grass	Yellow	Carotenoids	Briganti, 1842
	Rumex acetosa L.	Dry roots<br><br>Fresh roots	Red<br><br>Yellow	PAHs, Carotenoids, Anthocyanins<br>Carotenoids	Ucria, 1789<br>Briganti, 1842<br>Guarrera, 2006(b)<br>Angelini et al., 2010
	Rumex alpinus L.	Roots	Yellow	Carotenoids	Briganti, 1842<br>Angelini et al., 2010
	Rumex aquaticus L.	Fresh roots	Yellow	Carotenoids	Briganti, 1842<br>Angelini et al., 2010
	Rumex sanguineus L.	Grass	Yellow	Carotenoids	Briganti, 1842<br>Angelini et al., 2010
	Rumex acutus L.	Roots	Yellow	Carotenoids	Briganti, 1842

					Angelini et al., 2010
	Rumex maritimus L.	Roots	Yellow	Carotenoids	Briganti, 1842<br>Angelini et al., 2010
**Caryophyllaceae**					
	Stellaria alsine Grimm	Whole plant	Green	Clorophylls	Briganti, 1842
	Scleranthus perennis L.	Roots	Yellow	Carotenoids	Briganti, 1842
	Silene vulgaris (Moench) Garcke S. subsp. *vulgaris*	Leaves<br>Flowery stem	Green	Clorophylls	Briganti, 1842
	Saponaria officinalis L.	Pollen	Red	PAHs, Carotenoids, Anthocyanins	Briganti, 1842<br>Angelini et al., 2010
**Amaranthaceae s.l.**					
	Celosia argentea L.	Flower	Red	PAHs, Carotenoids, Anthocyanins	Briganti, 1842<br>Patil et al., 2019
	Beta vulgaris L. subsp. *vulgaris*	Roots	Red	PAHs, Carotenoids, Anthocyanins	Briganti, 1842<br>Patil et al., 2019
	Chenopodium vulvaria L.	Whole plant	Yellow	Carotenoids	Briganti, 1842
	Spinacia oleracea L.	Leaves	Green	Clorophylls	Briganti, 1842
	Atriplex hortensis L.	Leaves<br>Stem	Yellow	Carotenoids	Briganti, 1842<br>Angelini et al., 2010
**Phytolaccaceae**					
	Phytolacca americana L.	Juice of berries	Red	PAHs, Carotenoids, Anthocyanins	Briganti, 1842<br>Guarrera, 2006(b)<br>Maxia et al., 2013<br>Kakhia, 2015
**Cactaceae**					
	Opuntia ficus-indica (L.) Mill.	Unknown	Unknown	Unknown	Guarrera, 2006(b)
**Basellaceae**					
	Basella alba L.	Berries	Purple	Anthocyanins	Ucria, 1789<br>Briganti, 1842<br>Patil et al., 2019
**Buxaceae**					
	Buxus sempervirens L.	Leaves<br>Branches	Green	Clorophylls	Briganti, 1842<br>Guarrera, 2006(b)
**Hypericaceae**					
	Hypericum perforatum L.	Flower<br>Flowey plant	Yellow	Carotenoids	Ucria, 1789<br>Briganti, 1842

					Guarrera, 2006(b)<br>Angelini et al., 2010
**Euphorbiaceae**					
	Chrozophora tinctoria (L.) A.Juss.	Juice of fruits<br>Flowered bunches	Blue	Indigoidines	Ucria, 1789<br>Briganti, 1842<br>Guarrera, 2006(a)<br>Guarrera, 2006(b)
	Mercurialis annua L.	Leaves<br>Stem	Yellow	Carotenoids	Briganti, 1842<br>Guarrera, 2006(a)<br>Guarrera,2006(b)
	Mercurialis perennis L.	Grass	Blue	Indigoidines	Briganti, 1842
	Mercurialis corsica Coss. & Kralik	Unknown	Unknown	Unknown	Guarrera, 2006(b)
	Ricinus communis L.	Leaves<br>Spike	Yellow	Carotenoids	Briganti, 1842
	Euphorbia helioscopia L.	Whole plant	Green	Clorophylls	Briganti, 1842<br>Türkmen et al., 2004<br>Guarrera, 2006(b)
	Euphorbia palustris L.	Whole plant	Yellow	Carotenoids	Briganti, 1842<br>Türkmen et al., 2004<br>Guarrera, 2006(b)
	Euphorbia cyparissias L.	Whole plant	Green	Clorophylls	Briganti, 1842<br>Türkmen et al., 2004<br>Guarrera, 2006(b)
	Euphorbia characias L.	Whole plant	Yellow	Carotenoids	Guarrera, 2006(a)<br>Guarrera, 2006(b)<br>Caneva et al., 2013
	Euphorbia peplus L.	Whole plant	Yellow	Carotenoids	Briganti, 1842<br>Türkmen et al., 2004<br>Guarrera, 2006(b)
**Celastraceae**					
	Euonymus europaeus L.	Bark<br><br>Capsule<br>Charred branches	Red<br><br>Yellow<br>Black	PAHs, Carotenoids, Anthocyanins<br>Carotenoids<br>Anthocyanins, Tannins, PAHs	Briganti, 1842<br>Maxia et al., 2013
**Violaceae**					
	Viola odorata L.	Flower	Blue	Indigoidines	Briganti, 1842

					Guarrera, 2006(b)
	Viola tricolor L.	Grass	Yellow	Carotenoids	Briganti, 1842<br>Guarrera, 2006(b)
**Salicaceae**					
	Salix alba L.	Debarked wood<br><br>Bark<br>Branches	Red<br><br>Yellow	PAHs, Carotenoids, Anthocyanins<br>Carotenoids	Briganti, 1842<br>Guarrera, 2006(a)<br>Guarrera, 2006(b)
	Salix alba subsp. *vitellina* (L.) Schübl. & Martens	Branches	Yellow	Carotenoids	Briganti, 1842<br>Guarrera, 2006(b)
	Salix purpurea L.	Bark	Yellow	Carotenoids	Briganti, 1842<br>Guarrera, 2006(b)
	Salix pentandra L.	Leaves	Yellow	Carotenoids	Ucria, 1789<br>Briganti, 1842<br>Guarrera, 2006(b)
	Salix caprea L.	Debarked wood<br><br>Bark<br>Branches<br>Wood	Red<br><br>Yellow	PAHs, Carotenoids, Anthocyanins<br>Carotenoids	Briganti, 1842<br>Guarrera, 2006(b)<br>Kakhia, 2015
	Salix atrocinerea Brot.	Leaves	Yellow	Carotenoids	Guarrera, 2006(b)
	Populus alba L.	Young branches	Yellow	Carotenoids	Briganti, 1842<br>Farina, 1874
	Populus tremula L.	Bark	Yellow	Carotenoids	Briganti, 1842
	Populus nigra L.	Bark	Yellow	Carotenoids	Briganti, 1842<br>Guarrera, 2006(a)<br>Guarrera, 2006(b)<br>Maxia et al., 2013
**Linaceae**					
	Linum usitatissimum L.	Soot	Black	Anthocyanins, Tannins, PAHs	Briganti, 1842
**Fabaceae**					
	Cercis siliquastrum L.	Branches	Yellow	Carotenoids	Briganti, 1842
	Gleditsia triacanthos L.	Young branches<br>Thorns	Red	PAHs, Carotenoids, Anthocyanins	Briganti, 1842
	Vachellia farnesiana (L.) Wight & Arn.	Flower	Yellow	Carotenoids	Briganti, 1842

	Anagyris foetida L.	Leaves	Yellow	Carotenoids	Briganti, 1842
	Laburnum anagyroides Medik.	Leaves	Green	Clorophylls	Briganti, 1842
	Cytisus hirsutus L.	Leaves<br>Branches	Green	Clorophylls	Briganti, 1842
	Genista tinctoria L.	Stem<br>Flowery branches	Yellow	Carotenoids	Ucria 1789<br>Briganti, 1842<br>Kerry & David, 2001<br>Guarrera, 2006(a)<br>Angelini et al., 2010<br>Cardon, 2010<br>Kakhia, 2015
	Genista pilosa L.	Flowery branches	Yellow	Carotenoids	Briganti, 1842
	Spartium junceum L.	Young branches	Yellow	Carotenoids	Briganti, 1842<br>Guarrera, 2006(b)
	Ulex europaeus L.	Fresh flower	Yellow	Carotenoids	Briganti, 1842
	Indigofera tinctoria L.	Leaves	Blue	Indigotines	Ucria 1789<br>Briganti, 1842<br>Kerry & David, 2001<br>Guarrera, 2006(a)<br>Guarrera, 2006(b)<br>Angelini et al., 2010<br>Cardon, 2010<br>Caneva et al., 2013<br>Kakhia, 2015
	Robinia pseudoacacia L.	Dry wood<br>Young branches	Yellow	Carotenoids	Briganti, 1842
	Robinia hispida L.	Dry branches<br>Bark	Yellow	Carotenoids	Briganti, 1842
	Colutea arborescens L.	Branches	Green	Clorophylls	Briganti, 1842
	Amorpha fruticosa L.	Tender shoots<br>Leaves	Blue	Indigoidines	Briganti, 1842
	Phaseolus coccineus L.	Flower	Red	PAHs, Carotenoids,<br>Anthocyanins	Briganti, 1842
	Lathyrus aphaca L	Fresh plant	Green	Clorophylls	Briganti, 1842
	Ononis natrix L.	Grass	Green	Clorophylls	Briganti, 1842
	Ononis spinosa L.	Flowery stem	Yellow	Carotenoids	Briganti, 1842
	Trigonella foenum-graecum L.	Grass	Yellow	Carotenoids	Briganti, 1842

	Medicago sativa L.	Dry grass	Yellow	Carotenoids	Briganti, 1842
	Trifolium pratense L.	Grass<br>Flower	Yellow<br>Green	Carotenoids<br>Clorophylls	Ucria, 1789<br>Briganti, 1842
	Lotus tenuis Waldst. & Kit.	Dry flower	Blue	Indigoidines	Briganti, 1842
	Lotus corniculatus L.	Dry flower	Blue	Indigoidines	Briganti, 1842
	Lotus halophilus Boiss. & Spruner	Dry flower	Blue	Indigoidines	Briganti, 1842
	Lotus hirsutus (L.) Ser.	Leaves	Yellow	Carotenoids	Briganti, 1842
	Anthyllis vulneraria L.	Grass<br>Flower	Yellow<br>Blue	Carotenoids<br>Indigoidines	Ucria, 1789<br>Briganti, 1842
	Coronilla valentina subsp. *glauca* (L.) Batt.	Stem<br>Leaves	Green	Clorophylls	Briganti, 1842
	Sulla coronaria (L.) Medik.	Fresh flower	Brown	Tannins	Briganti, 1842
**Rhamnaceae**					
	Paliurus spina-christi Mill.	Leaves<br>Young branches	Yellow	Carotenoids	Briganti, 1842
	Rhamnus alaternus L.	Leaves<br>Young branches<br><br><br><br>Ripe berries<br>Bark	Yellow<br><br><br><br><br>Green<br>Black	Carotenoids<br><br><br><br><br>Clorophylls<br>Brown	Ucria, 1789<br>Briganti, 1842<br>Atzei, 2003<br>Guarrera, 2006(a)<br>Guarrera, 2006(b)<br>Maxia et al., 2013<br>Caneva et al., 2013<br>Guarrera, 1990
	Rhamnus infectoria L.	Peel of green berries<br>Ripe berries	Yellow<br>Green	Carotenoids<br>Clorophylls	Briganti, 1842<br>Atzei, 2003<br>Guarrera, 2006(a)<br>Caneva et al., 2013
	Rhamnus cathartica L.	Overripe berries<br><br>Bark<br>Green berries<br>Roots<br>Juice of ripe berries	Red<br><br>Yellow<br><br><br>Green	PAHs, Carotenoids, Anthocyanins<br>Carotenoids<br><br><br>Clorophylls	Ucria, 1789<br>Briganti, 1842<br>Tammaro, 1984<br><br><br>Caneva et al., 2013
	Frangula alnus Mill. subsp. *alnus*	Bark<br>Berries<br>Leaves	Yellow	Carotenoids	Ucria, 1789<br>Briganti, 1842
**Elaeagnaceae**					

	Hippophae rhamnoides L.	Branches	Red	PAHs, Carotenoids, Anthocyanins	Briganti, 1842
	Elaeagnus angustifolia L	Branches<br>Leaves	Red	PAHs, Carotenoids, Anthocyanins	Briganti, 1842
**Ulmaceae**					
	Celtis australis L.	Branches<br>Bark<br>Wood	Yellow<br><br>Brown	Carotenoids<br><br>Tannins	Briganti, 1842<br>Guarrera, 2006(b)
	Ulmus minor Mill.	Leaves	Red	PAHs, Carotenoids, Anthocyanins	Guarrera, 2006(b)
**Moraceae**					
	Broussonetia papyrifera (L.) Vent.	Branches	Yellow	Carotenoids	Briganti, 1842
	Morus nigra L.	Wood	Yellow	Carotenoids	Briganti, 1842<br>Guarrera, 2006(a)<br>Guarrera, 2006(b)<br>Kakhia, 2015
	Morus alba L.	Wood	Yellow	Carotenoids	Briganti, 1842
**Cannabaceae**					
	Humulus lupulus L.	Leaves<br>Flowery stem	Red	PAHs, Carotenoids, Anthocyanins	Briganti, 1842
	Cannabis sativa L.	Grass	Yellow	Carotenoids	Briganti, 1842
**Urticaceae**					
	Urtica dioica L.	Roots<br><br><br><br>Leaves	Yellow<br><br><br><br>Green	Carotenoids<br><br><br><br>Clorophylls	Ucria, 1789<br>Briganti, 1842<br>Guarrera, 2006(a)<br>Guarrera, 2006(b)<br>Kakhia, 2015<br>Caneva et al., 2013
	Parietaria officinalis L.	Leaves<br>Fresh stem	Green	Clorophylls	Briganti, 1842
	Parietaria judaica L.	Unknown	Unknown	Unknown	Guarrera, 2006(b)
**Coriariaceae**					
	Coriaria myrtifolia L.	Whole plant	Black	Anthocyanins, Tannins, PAHs	Briganti, 1842<br>Kakhia, 2015
**Betulaceae**					
	Betula pubescens Ehrh. var. *pubescens*	Branches<br>Wood	Red	PAHs, Carotenoids, Anthocyanins	Briganti, 1842<br>Kakhia, 2015

		Bark<br>Leaves	 Yellow	 Carotenoids	
	Alnus glutinosa (L.) Gaertn.	Bark<br><br>Leaves<br>Bark<br>Young branches<br>Bark<br><br>Cones	Red<br><br>Yellow<br><br><br>Brown<br><br>Black	PAHs, Carotenoids,<br>Anthocyanins<br>Carotenoids<br><br><br>Tannins<br><br>Anthocyanins,<br>Tannins, PAHs	Briganti, 1842<br>Farina, 1874<br>Schneider, 1981<br>Curreli & Loddo, 1983<br>Atzei, 2003<br>Türkmen et al., 2004<br>Caneva et al., 2013<br>Guarrera, 2006(a)<br>Guarrera, 2006(b)<br>Maxia et al., 2013<br>Kakhia, 2015
	Alnus cordata (Loisel.) Duby	Inner bark<br><br>Bark<br>Cones<br>Catkins	Red<br><br>Yellow<br><br>Green	PAHs, Carotenoids,<br>Anthocyanins<br>Carotenoids<br><br>Clorophylls	Briganti, 1842<br>Schneider, 1981<br>Curreli & Loddo, 1983<br>Atzei, 2003<br>Kakhia, 2015
	Corylus avellana L.	Bark<br>Branches	Green	Clorophylls	Briganti, 1842
	Carpinus betulus L.	Bark	Yellow	Carotenoids	Briganti, 1842
**Fagaceae**					
	Fagus sylvatica L.	Fresh bark<br>Calyxes<br>Branches<br>Charred wood	Yellow<br><br><br>Black	Carotenoids<br><br><br>Anthocyanins,<br>Tannins, PAHs	Briganti, 1842<br><br><br>Caneva et al., 2013
	Castanea sativa Mill.	Bark<br>Wood<br>Fruit rind	Brown	Tannins	Briganti, 1842
	Quercus suber L.	Charred bark<br>Galls	Black	Anthocyanins,<br>Tannins, PAHs	Briganti, 1842<br>Schneider, 1981<br>Curreli & Loddo, 1983<br>Tammaro, 1984<br>Atzei, 2003<br>Türkmen et al., 2004<br>Guarrera, 2006(b)

					Caneva et al., 2013<br>Kakhia, 2015
	Quercus ilex L.	Galls	Black	Anthocyanins, Tannins, PAHs	Guarrera, 2006(b)
	Quercus coccifera L.	Galls	Black	Anthocyanins, Tannins, PAHs	Guarrera, 2006(b)
	Quercus congesta C.Presl	Galls	Black	Anthocyanins, Tannins, PAHs	Guarrera, 2006(b)
	Quercus pubescens Willd.	Galls	Black	Anthocyanins, Tannins, PAHs	Guarrera, 2006(b)
	Quercus robur L.	Bark<br>Branches<br>Exudate<br>Top of acorn<br>Galls	Brown<br><br><br>Black	Tannins<br><br><br>Anthocyanins,<br>Tannins, PAHs	Ucria, 1789<br>Briganti, 1842<br>Schneider, 1981<br>Curreli & Loddo, 1983<br>Tammaro, 1984<br>Atzei, 2003<br>Türkmen et al., 2004<br>Angelini et al., 2010<br>Caneva et al., 2013<br>Kakhia, 2015
	Quercus petraea (Matt.) Liebl.	Bark<br>Branches<br>Exudate<br>Top of acorn<br>Galls	Brown<br><br><br>Black	Tannins<br><br><br>Anthocyanins,<br>Tannins, PAHs	Briganti, 1842<br>Schneider, 1981<br>Curreli & Loddo, 1983<br>Tammaro, 1984<br>Atzei, 2003<br>Türkmen et al., 2004<br>Angelini et al., 2010<br>Caneva et al., 2013<br>Kakhia, 2015
	Quercus ithaburensis subsp. *macrolepis* (Kotschy) Hedge & Yalt.	Bark<br>Branches<br>Exudate<br>Top of acorn<br>Galls	Brown<br><br><br>Black	Tannins<br><br><br>Anthocyanins,<br>Tannins, PAHs	Ucria, 1789<br><br><br><br>Caneva et al., 2013
**Juglandaceae**					
	Juglans regia L.	Bark<br>Catkin	Yellow	Carotenoids	Briganti, 1842<br>Cabiddu, 1965

		Husk<br>Bark of roots<br>Leaves<br>Fruit	Brown	Tannins	Tammaro, 1984<br>Guarrera, 1990<br>Özgökçe & Yilmaz, 2003<br>Dogan et al., 2004<br>Türkmen et al., 2004<br>Guarrera, 2006(a)<br>Guarrera, 2006(b)<br>Caneva et al., 2013<br>Maxia et al., 2013<br>Kakhia, 2015
**Rosaceae**					
	Physocarpus opulifolius (L.) Maxim.	Leafless stem	Red	PAHs, Carotenoids, Anthocyanins	Briganti, 1842
	Filipendula ulmaria (L.) Maxim.	Leaves<br>Flowery stem	Yellow	Carotenoids	Briganti, 1842
	Rubus odoratus L.	Dry stem	Yellow	Carotenoids	Briganti, 1842<br>Guarrera, 2006(b)
	Rosa pendulina L.	Young branches	Yellow	Carotenoids	Briganti, 1842<br>Guarrera, 2006(b)
	Rosa rubiginosa L.	Young branches	Yellow	Carotenoids	Briganti, 1842<br>Guarrera, 2006(b)
	Rosa canina L.	Roots<br>Wood	Yellow	Carotenoids	Briganti, 1842<br>Guarrera, 2006(b)
	Agrimonia eupatoria L.	Leaves<br>Stem	Yellow	Carotenoids	Briganti, 1842<br>Guarrera, 2006(b)
	Amelanchier ovalis Medik	Unknown	Unknown	Unknown	Guarrera, 2006(b)
	Sanguisorba officinalis L.	Whole plant	Brown	Tannins	Briganti, 1842
	Fragaria vesca L.	Roots<br><br>Fruits	Red<br><br>Yellow	PAHs, Carotenoids, Anthocyanins<br>Carotenoids	Briganti, 1842
	Potentilla anserina L.	Leaves	Yellow	Carotenoids	Briganti, 1842
	Potentilla erecta (L.) Raeusch.	Roots	Red	PAHs, Carotenoids, Anthocyanins	Briganti, 1842
	Pyrus communis L.	Bark<br>Wood<br>Dry fruits	Yellow<br><br>Brown	Carotenoids<br><br>Tannins	Briganti, 1842<br>Patil et al., 2019

	Cydonia oblonga Mill.	Leaves	Yellow	Carotenoids	Briganti, 1842
	Malus domestica Borkh.	Bark<br>Leaves<br>Dry wood	Yellow<br><br>Brown	Carotenoids<br><br>Tannins	Ucria, 1789<br>Briganti, 1842<br>Guarrera, 2006(b)
	Sorbus domestica L.	Young branches<br><br>Fruits	Red<br><br>Brown	PAHs, Carotenoids, Anthocyanins<br>Tannins	Briganti, 1842
	Sorbus aucuparia L.	Young branches	Red	PAHs, Carotenoids, Anthocyanins	Briganti, 1842
	Pyracantha coccinea M. J. Roemer	Branches	Red	PAHs, Carotenoids, Anthocyanins	Briganti, 1842
	Mespilus germanica L.	Bark<br>Wood<br>Branches<br>Fruits	Red	PAHs, Carotenoids, Anthocyanins	Briganti, 1842
	Crataegus monogyna Jacq.	Wood	Red	PAHs, Carotenoids, Anthocyanins	Briganti, 1842
	Prunus avium (L.) L.	Dry wood	Yellow	Carotenoids	Briganti, 1842
	Prunus cerasus L.	Fruits	Red	PAHs, Carotenoids, Anthocyanins	Briganti, 1842<br>Guarrera, 2006(b)
	Prunus dulcis (Mill.) D.A. Webb	Unknown	Unknown	Unknown	Guarrera, 2006(b)
	Prunus mahaleb L.	Young soft branches	Yellow	Carotenoids	Briganti, 1842<br>Guarrera, 2006
	Prunus persica (L.) Stokes	Young branches<br>Kernel	Red	PAHs, Carotenoids, Anthocyanins	Briganti, 1842<br>Patil et al., 2019
	Prunus armeniaca L.	Young branches	Yellow	Carotenoids	Briganti, 1842
	Prunus domestica L.	Bark<br>Wood<br>Dry fruits	Yellow	Carotenoids	Briganti, 1842
	Prunus laurocerasus L.	Branches<br>Leaves	Red	PAHs, Carotenoids, Anthocyanins	Briganti, 1842
	Prunus spinosa L.	Unknown	Unknown	Unknown	Guarrera, 2006(b)
**Geraniaceae**					
	Geranium robertianum L.	Grass	Yellow	Carotenoids	Briganti, 1842
	Geranium colombinum L.	Grass	Yellow	Carotenoids	Briganti, 1842
	Pelargonium inquinans (L.) L'Hér.	Corolla	Red	PAHs, Carotenoids, Anthocyanins	Briganti, 1842

	Erodium moschatum (L.) L'Hér.	Grass	Yellow	Carotenoids	Briganti, 1842
**Lythraceae**					
	Lythrum salicaria L.	Flowery stem	Brown	Tannins	Briganti, 1842
	Punica granatum L.	Peel of fruits	Yellow	Carotenoids	Briganti, 1842<br>Cabiddu, 1965<br>Curreli & Loddo, 1983<br>Atzei, 2003<br>Türkmen et al., 2004<br>Guarrera, 2006(b)<br>Angelini et al., 2010<br>Maxia et al., 2013<br>Kakhia, 2015<br>Patil et al., 2019
**Myrtaceae**					
	Myrtus communis L.	Juice of berries<br>Leaves	Brown	Tannins	Briganti, 1842<br>Cabiddu, 1965<br>Curreli & Loddo, 1983<br>Atzei, 2003<br>Türkmen et al., 2004<br>Guarrera, 2006(b)
**Staphyleaceae**					
	Staphylea pinnata L.	Leafy branches	Yellow	Carotenoids	Briganti, 1842
**Resedaceae**					
	Reseda lutea L.	Whole plant	Yellow	Carotenoids	Guarrera, 2006(b)
	Reseda luteola L.	Whole plant	Yellow	Carotenoids	Ucria, 1789<br>Briganti, 1842<br>Kerry & David, 2001<br>Özgökçe & Yilmaz, 2003<br>Dogan et al., 2004<br>Guarrera, 2006(a)<br>Guarrera, 2006(b)<br>Angelini et al., 2010<br>Cardon, 2010<br>Caneva et al., 2013<br>Kakhia, 2015<br>Angelini, 2016

					Fioretti, 2016
**Brassicaceae**					
	Sisymbrium officinale (L.) Scop.	Spikes	Green	Clorophylls	Briganti, 1842
	Isatis tinctoria L.	Leaves	Blue	Indigoidines	Ucria, 1789<br>Briganti, 1842<br>Kerry & David, 2001<br>Türkmen et al., 2004<br>Guarrera, 2006(a)<br>Guarrera, 2006(b)<br>Angelini et al., 2010<br>Idolo et al., 2010<br>Caneva et al., 2013<br>Maxia et al., 2013<br>Kakhia, 2015<br>Angelini, 2016<br>Fioretti, 2016
	Erysimum cheiri (L.) Crantz	Leaves<br>Stem	Green	Clorophylls	Briganti, 1842
	Matthiola incana (L.) W.T.Aiton	Leaves<br>Stem	Green	Clorophylls	Briganti, 1842
	Matthiola sinuata (L.) W.T.Aiton	Flower	Red	PAHs, Carotenoids, Anthocyanins	Briganti, 1842
	Barbarea vulgaris (L.) W.T. Aiton	Leaves	Green	Clorophylls	Briganti, 1842
	Capsella bursa-pastoris (L.) Medik.	Plant<br>Seed<br>Leaves	Yellow	Carotenoids	Briganti, 1842
	Thlaspi arvense L.	Green plant<br>Green seed	Green	Clorophylls	Briganti, 1842
	Raphanus sativus L.	Epidermis of the roots	Purple	Anthocyanins	Briganti, 1842
**Thymelaeaceae**					
	Daphne mezereum L.	Stem	Green	Clorophylls	Briganti, 1842
	Daphne laureola L.	Wood<br>Leaves	Yellow	Carotenoids	Briganti, 1842
	Daphne gnidium L.	Leaves<br>Bark	Yellow	Carotenoids	Briganti, 1842<br>Cabiddu, 1965<br>Guarrera, 1990
		Bark	Green	Clorophylls	Atzei, 2003

					Guarrera, 2006(a)<br>Guarrera, 2006(b)<br>Maxia et al., 2013
**Cistaceae**					
	Helianthemum nummularium (L.) Mill. subsp. *nummularium*	Root<br>Stem	Brown	Tannins	Briganti, 1842
**Malvaceae**					
	Malva arborea (L.) Webb & Berthel.	Stem<br>Leaves	Yellow	Carotenoids	Briganti, 1842
	Alcea rosea L.	Flower	Red	PAHs, Carotenoids, Anthocyanins	Briganti, 1842<br>Patil et al., 2019
	Hibiscus syriacus L.	Wood<br><br>Flower	Red<br><br>Purple	PAHs, Carotenoids, Anthocyanins<br>Anthocyanins	Briganti, 1842
	Tilia × europaea L.	Green bark	Red	PAHs, Carotenoids, Anthocyanins	Briganti, 1842
**Rutaceae**					
	Ruta graveolens L.	Leaves<br>Stem	Green	Clorophylls	Briganti, 1842<br>Angelini et al., 2010
	Citrus medica L.	Unripe fruits	Yellow	Carotenoids	Briganti, 1842<br>Patil et al., 2019
	Citrus aurantium L.	Unripe fruits<br>Peel of ripe fruits	Yellow<br>Red	Carotenoids<br>PAHs, Carotenoids, Anthocyanins	Briganti, 1842
**Meliaceae**					
	Melia azedarach L.	Branches	Red	PAHs, Carotenoids, Anthocyanins	Briganti, 1842
**Anacardiaceae**					
	Rhus coriaria L.	Bark of roots<br><br><br>Bark	Red<br><br><br>Yellow	PAHs, Carotenoids, Anthocyanins<br><br>Carotenoids	Briganti, 1842<br>Tammaro, 1984<br>Caneva et al., 2013<br>Türkmen et al., 2004<br>Guarrera, 2006(b)<br>Idolo et al., 2010<br>Kakhia, 2015
	Rhus typhina L.	Bark	Yellow	Carotenoids	Briganti, 1842<br>Kakhia, 2015

	Cotinus coggygria Scop.	Bark of roots	Red	PAHs, Carotenoids, Anthocyanins	Briganti, 1842<br>Kerry & David, 2001<br>Caneva et al., 2013
		Bark<br>Branches<br>Leaves	Yellow	Carotenoids	Türkmen et al., 2004<br>Guarrera, 2006(b)<br>Angelini et al., 2010
	Pistacia lentiscus L.	Leaves	Red	PAHs, Carotenoids, Anthocyanins	Guarrera, 2006(b)
	Pistacia terebinthus L.	Unknown	Unknown	Unknown	Guarrera, 2006(b)
**Sapindaceae**					
	Acer campestre L.	Bark<br>Fresh wood	Red	PAHs, Carotenoids, Anthocyanins	Briganti, 1842<br>Kakhia, 2015
	Acer opalus subsp. *obtusatum* (Waldst. & Kit. ex Willd.) Gams	Bark	Red	PAHs, Carotenoids, Anthocyanins	Briganti, 1842<br>Kakhia, 2015
	Acer pseudoplatanus L.	Bark	Red	PAHs, Carotenoids, Anthocyanins	Briganti, 1842<br>Kakhia, 2015
	Aesculus hippocastanum L.	Bark<br>Young branches<br>Leaves	Yellow	Carotenoids	Briganti, 1842
	Aesculus pavia L.	Bark<br>Young branches<br>Leaves	Yellow	Carotenoids	Briganti, 1842
**Cornaceae**					
	Cornus mas L.	Bark of roots	Yellow	Carotenoids	Briganti, 1842
	Cornus sanguinea L.	Branches<br>Bark<br>Berries	Yellow<br>Green<br>Purple	Carotenoids<br>Clorophylls<br>Anthocyanins	Briganti, 1842<br>Guarrera, 2006(b)
**Balsaminaceae**					
	Impatiens balsamina L.	Flower	Yellow	Carotenoids	Briganti, 1842<br>Patil et al., 2019
**Ebenaceae**					
	Diospyros lotus L.	Leaves<br>Branches	Yellow	Carotenoids	Briganti, 1842
**Hydrangeaceae**					
	Philadelphus coronarius L.	Branches	Red	PAHs, Carotenoids, Anthocyanins	Briganti, 1842
**Myrsinaceae**					

	Cyclamen hederifolium Aiton subsp. *hederifolium*	Tuber	Red	PAHs, Carotenoids, Anthocyanins	Briganti, 1842
	Cyclamen purpurascens Mill. subsp. *purpurascens*	Tuber	Red	PAHs, Carotenoids, Anthocyanins	Briganti, 1842
	Cyclamen repandum Sm.	Unknown	Unknown	Unknown	Guarrera, 2006(b)
	Lysimachia vulgaris L.	Grass<br>Stem<br>Flower<br>Roots	Yellow<br><br><br>Grey	Carotenoids<br><br><br>PAHs, Tannins	Ucria, 1789<br>Briganti, 1842
**Ericaceae**					
	Erica arborea L.	Branches	Red	PAHs, Carotenoids, Anthocyanins	Briganti, 1842<br>Curreli & Loddo, 1983<br>Atzei, 2003<br>Guarrera, 2006(b)<br>Maxia et al., 2013
	Calluna vulgaris (L.) Hull	Branches<br><br>Whole plant	Red<br><br>Yellow	PAHs, Carotenoids, Anthocyanins<br>Carotenoids	Briganti, 1842
	Arctostaphylos uva-ursi (L.) Spreng. subsp. *uva-ursi*	Roots<br>Whole plant<br>Leaves<br>Leaves	Red<br><br>Grey<br>Black	PAHs, Carotenoids, Anthocyanins<br>PAHs, Tannins<br>Anthocyanins, Tannins, PAHs	Ucria, 1789<br>Briganti, 1842<br>Guarrera, 2006(a)
	Vaccinium myrtillus L.	Berries	Purple	Anthocyanins	Briganti, 1842<br>Guarrera, 2006(b)
**Rubiaceae**					
	Crucianella latifolia L.	Roots	Red	PAHs, Carotenoids, Anthocyanins	Briganti, 1842
	Asperula tinctoria L.	Roots	Red	PAHs, Carotenoids, Anthocyanins	Ucria, 1789<br>Briganti, 1842
	Asperula cynanchica L.	Roots	Red	PAHs, Carotenoids, Anthocyanins	Briganti, 1842
	Asperula purpurea (L.) Ehrend.	Roots	Red	PAHs, Carotenoids, Anthocyanins	Briganti, 1842
	Asperula taurina L.	Roots	Red	PAHs, Carotenoids, Anthocyanins	Briganti, 1842

	Asperula laevigata L.	Roots	Red	PAHs, Carotenoids, Anthocyanins	Briganti, 1842
	Asperula arvensis L.	Roots	Red	PAHs, Carotenoids, Anthocyanins	Briganti, 1842
	Galium boreale L.	Roots	Red	PAHs, Carotenoids, Anthocyanins	Briganti, 1842
	Galium verum L.	Roots<br><br>Flowery grass<br>Spike	Red<br><br>Yellow	PAHs, Carotenoids, Anthocyanins<br>Carotenoids	Briganti, 1842<br>Kerry & David, 2001<br>Türkmen et al., 2004<br>Guarrera, 2006(b)<br>Angelini et al., 2010
	Galium sylvaticum L.	Roots	Red	PAHs, Carotenoids, Anthocyanins	Briganti, 1842
	Galium mollugo L.	Roots<br><br>Leaves<br>Stem	Red<br><br>Yellow	PAHs, Carotenoids, Anthocyanins<br>Carotenoids	Briganti, 1842<br>Kerry & David, 2001
	Galium aparine L.	Roots	Red	PAHs, Carotenoids, Anthocyanins	Briganti, 1842<br>Kerry & David, 2001
	Cruciata laevipes Opiz	Roots	Red	PAHs, Carotenoids, Anthocyanins	Briganti, 1842
	Rubia peregrina L.	Roots	Red	PAHs, Carotenoids, Anthocyanins	Briganti, 1842<br>Guarrera, 1990<br>Guarrera, 2006(a)<br>Guarrera, 2006(b)<br>Maxia et al., 2013<br>Kakhia, 2015
	Rubia tinctorum L.	Roots	Red	PAHs, Carotenoids, Anthocyanins	Ucria, 1789<br>Briganti, 1842<br>Farina, 1874<br>Cabiddu, 1965<br>Zanoni & Schofield, 1983<br>Kerry & David, 2001<br>Özgökçe & Yilmaz, 2003<br>Dogan et al., 2004

					Türkmen et al., 2004<br>Guarrera, 2006(a)<br>Guarrera, 2006(b)<br>Angelini et al., 2010<br>Idolo et al., 2010<br>Caneva et al., 2013<br>Maxia et al., 2013<br>Kakhia, 2015<br>Angelini, 2016
**Apocynaceae**					
	Nerium oleander L.	Branches<br>Leaves	Yellow	Carotenoids	Briganti, 1842
	Vinca major L.	Stem<br>Leaves	Yellow	Carotenoids	Briganti, 1842
	Periploca graeca L.	Whole plant	Brown	Tannins	Briganti, 1842
	Asclepias syriaca L.	Stem<br>Leaves	Green	Clorophylls	Briganti, 1842
	Vincetoxicum hirundinaria Medik. s.l. subsp. *hirundinaria*	Stem<br>Leaves	Green	Clorophylls	Briganti, 1842
**Boraginaceae**					
	Buglossoides arvensis (L.) I.M.Johnst.	Roots	Red	PAHs, Carotenoids, Anthocyanins	Briganti, 1842
	Alkanna tinctoria Tausch subsp. *tinctoria*	Roots<br><br>Leaves	Red<br><br>Yellow	PAHs, Carotenoids, Anthocyanins<br>Carotenoids	Ucria, 1789<br>Briganti, 1842<br>Cabiddu, 1965<br>Kerry & David, 2001<br>Guarrera, 2006(a)<br>Guarrera, 2006(b)<br>Caneva et al., 2013<br>Maxia et al., 2013<br>Kakhia, 2015
	Echium vulgare L.	Roots<br>Leaves<br>Stem	Grey<br>Green	PAHs, Tannins<br>Clorophylls	Briganti, 1842
	Symphytum officinale L.	Grass	Black	Anthocyanins, Tannins, PAHs	Briganti, 1842

	Anchusa ochroleuca M. Bieb.	Roots	Red	PAHs, Carotenoids, Anthocyanins	Briganti, 1842
		Leaves	Green	Clorophylls	
		Flower	Blue	Indigoidines	
	Lycopsis arvensis L.	Fresh grass<br>Flower	Yellow	Carotenoids	Briganti, 1842
	Borago officinalis L.	Leaves<br>Flowery stem	Yellow	Carotenoids	Briganti, 1842<br>Guarrera, 2006(b)
**Oleaceae**					
	Chrysojasminum fruticans (L.) Banfi	Young branches	Yellow	Carotenoids	Ucria, 1789
	Jasminum officinale L.	Young branches	Yellow	Carotenoids	Briganti, 1842
	Fraxinus ornus L.	Leaves	Yellow	Carotenoids	Briganti, 1842<br>Türkmen et al., 2004<br>Guarrera, 2006(a)<br>Guarrera, 2006(b)
			Green	Clorophylls	Idolo et al., 2010<br>Caneva et al., 2013
	Fraxinus excelsior L.	Fresh wood	Yellow	Carotenoids	Briganti, 1842
		Bark	Blue	Indigoidines	Guarrera, 2006(a)
	Syringa vulgaris L.	Branches	Yellow	Carotenoids	Briganti, 1842
	Ligustrum vulgare L.	Bark	Yellow	Carotenoids	Ucria, 1789
		Berries	Red	PAHs, Carotenoids, Anthocyanins	Briganti, 1842<br>Guarrera, 2006(b)
	Olea europaea L.	Young soft branches<br>Leaves	Yellow	Carotenoids	Briganti, 1842<br>Guarrera, 2006(b)
	Phillyrea angustifolia L.	Branches<br>Leaves	Yellow	Carotenoids	Guarrera, 2006(b)
	Phillyrea latifolia L.	Branches<br>Leaves	Yellow	Carotenoids	Briganti, 1842<br>Guarrera, 2006(b)<br>Maxia et al., 2013
**Verbenaceae**					
	Verbena officinalis L.	Flowery stem	Yellow	Carotenoids	Briganti, 1842
**Lamiaceae**					
	Marrubium vulgare L.	Stem<br>Flower	Yellow	Carotenoids	Briganti, 1842<br>Guarrera, 2006(b)
	Galeopsis ladanum L.	Flowery grass	Red	PAHs, Carotenoids, Anthocyanins	Briganti, 1842

	Galeopsis tetrahit L.	Flowery grass	Red	PAHs, Carotenoids, Anthocyanins	Briganti, 1842
	Lamium purpureum L.	Flowery stem	Green	Clorophylls	Briganti, 1842
	Ballota nigra L.	Stem<br>Flower	Green	Clorophylls	Briganti, 1842
	Stachys sylvatica L.	Grass	Yellow	Carotenoids	Ucria, 1789<br>Briganti, 1842
	Betonica officinalis L.	Leaves<br>Flowery stem	Yellow	Carotenoids	Briganti, 1842
	Prunella vulgaris L.	Flowery plant	Green	Clorophylls	Briganti, 1842
	Melissa officinalis L.	Leaves	Green	Clorophylls	Briganti, 1842<br>Türkmen et al., 2004
	Satureja hortensis L.	Flowery stem	Green	Clorophylls	Briganti, 1842
	Clinopodium acinos (L.) Kuntze	Stem<br>Leaves	Green	Clorophylls	Briganti, 1842
	Clinopodium nepeta (L.) Kuntze	Unknown	Unknown	Unknown	Guarrera, 2006(b)
	Clinopodium vulgare L.	Leaves<br>Flowery stem	Yellow	Carotenoids	Briganti, 1842
	Origanum vulgare L.	Top of flowering plant	Brown	Tannins	Briganti, 1842<br>Guarrera, 2006(b)
	Thymus vulgaris L.	Stem<br>Leaves	Green	Clorophylls	Briganti, 1842
	Lycopus europaeus L.	Stem<br>Leaves<br>Flower<br>Sap<br>Grass	Yellow<br><br><br>Black	Carotenoids<br><br><br>Anthocyanins, Tannins, PAHs	Ucria, 1789<br>Briganti, 1842
	Mentha aquatica L.	Stem<br>Leaves	Green	Clorophylls	Briganti, 1842
	Rosmarinus officinalis L.	Branches<br>Leaves	Yellow	Carotenoids	Briganti, 1842<br>Curreli & Loddo, 1983<br>Atzei, 2003<br>Guarrera, 2006(b)
	Lavandula angustifolia Mill.	Stem<br>Branches	Brown	Tannins	Briganti, 1842
	Lavandula angustifolia subsp. *pyrenaica* (DC.) Guinea	Stem<br>Branches	Brown	Tannins	Briganti, 1842

	Salvia officinalis L.	Stump<br>Woody stem	Yellow	Carotenoids	Briganti, 1842
	Vitex agnus-castus L.	Young branches	Green	Clorophylls	Briganti, 1842
**Scrophulariaceae**					
	Verbascum phlomoides L.	Leaves<br>Stem<br>Flower	Yellow	Carotenoids	Briganti, 1842
	Verbascum thapsus L.	Unknown	Unknown	Unknown	Guarrera, 2006(b)
	Scrophularia nodosa L.	Leaves<br>Stem	Yellow	Carotenoids	Briganti, 1842
**Orobanchaceae**					
	Melampyrum nemorosum L.	Flowery whole plant	Green	Clorophylls	Briganti, 1842
	Melampyrum pratense L.	Whole plant	Yellow	Carotenoids	Briganti, 1842
	Euphrasia officinalis L.	Grass<br>Green capsule	Brown	Tannins	Briganti, 1842
**Plantaginaceae**					
	Antirrhinum majus L.	Stem<br>Flower	Yellow	Carotenoids	Briganti, 1842
	Misopates orontium (L.) Raf. subsp. *orontium*	Fruited plant	Yellow	Carotenoids	Briganti, 1842
	Linaria vulgaris Mill. subsp. *vulgaris*	Stem<br>Flower	Yellow	Carotenoids	Briganti, 1842<br>Guarrera, 2006(a)<br>Guarrera, 2006(b)
	Veronica officinalis L.	Dry plant	Green	Clorophylls	Briganti, 1842
	Veronica serpyllifolia L.	Flowery plant	Yellow	Carotenoids	Briganti, 1842
	Veronica chamaedrys L.	Flowery plant	Yellow	Carotenoids	Briganti, 1842
	Veronica hederifolia L.	Flowery plant	Yellow	Carotenoids	Briganti, 1842
	Plantago lanceolata L.	Leaves	Green	Clorophylls	Briganti, 1842
**Bignoniaceae**					
	Campsis radicans (L.) Bureau	Branches	Red	PAHs, Carotenoids, Anthocyanins	Briganti, 1842
	Catalpa bignonioides Walter	Branches	Red	PAHs, Carotenoids, Anthocyanins	Briganti, 1842
**Acanthaceae**					
	Acanthus mollis L.	Grass	Yellow	Carotenoids	Ucria, 1789<br>Briganti, 1842

					Guarrera, 2006(b)
**Lentibulariaceae**					
	Pinguicula vulgaris L.	Juice of leaves	Red	PAHs, Carotenoids, Anthocyanins	Briganti, 1842
**Convolvulaceae**					
	Cuscuta europaea L.	Whole plant<br>Sap	Red	PAHs, Carotenoids, Anthocyanins	Ucria, 1789<br>Briganti, 1842
	Cuscuta epithymum (L.) L.	Whole plant	Red	PAHs, Carotenoids, Anthocyanins	Briganti, 1842
	Convolvulus sepium L.	Roots	Red	PAHs, Carotenoids, Anthocyanins	Briganti, 1842
	Convolvulus arvensis L.	Stem<br>Leaves<br>Flower	Brown	Tannins	Briganti, 1842
**Solanaceae**					
	Atropa belladonna L.	Berries	Green	Clorophylls	Briganti, 1842
	Physalis alkekengi L.	Calyxes<br>Juice of berries	Red	PAHs, Carotenoids, Anthocyanins	Briganti, 1842
	Solanum tuberosum L.	Green leaves<br>Flowery stem	Yellow	Carotenoids	Briganti, 1842
	Solanum lycopersicum L.	Leaves<br>Stem	Yellow<br>Green	Carotenoids<br>Clorophylls	Briganti, 1842<br>Guarrera, 2006(a)<br>Guarrera, 2006(b)
	Solanum nigrum L.	Unknown	Unknown	Unknown	Guarrera, 2006(b)
	Solanum dulcamara L.	Leaves<br>Flowery stem	Grey	PAHs, Tannins	Briganti, 1842
	Capsicum annuum L.	Stem<br>Leaves<br>Fruit	Yellow	Carotenoids	Briganti, 1842<br>Guarrera, 2006(a)<br>Guarrera, 2006(b)<br>Patil et al., 2019
	Nicotiana tabacum L.	Green leaves<br>Yellowed leaves	Yellow<br>Brown	Carotenoids<br>Tannins	Briganti, 1842<br>Patil et al., 2019
**Gentianaceae**					
	Centaurium erythraea Rafn	Flower	Yellow	Carotenoids	Guarrera, 2006(b)<br>Caneva et al., 2013
**Aquifoliaceae**					
	Ilex aquifolium L.	Leaves	Yellow	Carotenoids	Briganti, 1842

		Young branches			
**Araliaceae**					
	Hedera helix L.	Leaves<br><br>Sap	Black<br><br>Brown	Anthocyanins, Tannins, PAHs<br>Tannins	Briganti, 1842<br>Curreli & Loddo, 1983<br>Atzei, 2003<br>Guarrera, 2006(a)<br>Guarrera, 2006(b)<br>Maxia et al., 2013
**Apiaceae**					
	Eryngium campestre L.	Leaves<br>Stem<br>Shoots<br>Seed	Yellow	Carotenoids	Briganti, 1842
	Anthriscus sylvestris (L.) Hoffm.	Leaves<br>Green stem	Yellow	Carotenoids	Briganti, 1842
	Anthriscus cerefolium (L.) Hoffm.	Umbels<br>Grass	Yellow<br>Green	Carotenoids<br>Clorophylls	Briganti, 1842
	Scandix pecten-veneris L.	Grass<br>Seed	Yellow	Carotenoids	Briganti, 1842<br>Guarrera, 2006(b)
	Myrrhis odorata (L.) Scop.	Leaves<br>Green stem	Yellow	Carotenoids	Briganti, 1842
	Sium latifolium L.	Leaves<br>Flowery stem	Grey	PAHs, Tannins	Briganti, 1842
	Seseli libanotis (L.) W.D.J. Koch subsp. *libanotis*	Fresh plant	Yellow	Carotenoids	Briganti, 1842
	Oenanthe pimpinelloides L.	Yellowed stem	Green	Clorophylls	Briganti, 1842
	Foeniculum vulgare Mill.	Flowery stem	Yellow	Carotenoids	Briganti, 1842
	Angelica sylvestris L.	Roots<br>Leaves<br>Stem	Yellow	Carotenoids	Briganti, 1842<br>Kakhia, 2015
	Angelica archangelica L.	Leaves	Yellow	Carotenoids	Briganti, 1842
	Ferula communis L.	Flower	Yellow	Carotenoids	Briganti, 1842<br>Guarrera, 2006(b)<br>Maxia et al., 2013
	Thapsia asclepium L.	Umbels	Yellow	Carotenoids	Ucria, 1789<br>Briganti, 1842<br>Guarrera, 2006(b)

	Thapsia garganica L.	Leaves<br>Umbels	Green<br>Yellow	Clorophylls<br>Carotenoids	Briganti, 1842<br>Guarrera, 2006(b)
	Daucus carota L.	Sterile flower<br><br>Leaves<br>Stem	Red<br><br>Green	PAHs, Carotenoids, Anthocyanins<br>Clorophylls	Briganti, 1842<br>Angelini et al., 2010
	Daucus carota subsp. *maximus* (Desf.) Ball	Pistils	Blue	Indigoidines	Briganti, 1842
**Caprifoliaceae**					
	Lonicera alpigena L.	Young branches	Yellow	Carotenoids	Briganti, 1842
	Lonicera caprifolium L.	Young stem	Yellow	Carotenoids	Briganti, 1842
	Lonicera periclymenum L.	Young stem	Yellow	Carotenoids	Briganti, 1842
	Succisa pratensis Moench	Roots	Yellow	Carotenoids	Ucria, 1789
	Sixalix atropurpurea (L.) W.Greuter & Burdet	Whole plant	Green	Clorophylls	Guarrera, 2006(b)
**Campanulaceae**					
	Campanula pyramidalis L.	Flowery stem	Yellow	Carotenoids	Briganti, 1842
	Campanula rotundifolia L.	Flowery stem<br>Leaves<br>Flower	Green<br><br>Blue	Clorophylls<br><br>Indigoidines	Ucria, 1789<br>Briganti, 1842
**Menyanthaceae**					
	Menyanthes trifoliata L.	Grass	Yellow	Carotenoids	Briganti, 1842
**Asteraceae**					
	Eupatorium cannabinum L.	Flowery plant	Red	PAHs, Carotenoids, Anthocyanins	Briganti, 1842<br>Guarrera, 2006(b)
	Solidago virgaurea L.	Leaves<br>Flowery stem	Yellow	Carotenoids	Briganti, 1842<br>Kerry & David, 2001<br>Angelini et al., 2010<br>Cardon, 2010<br>Fioretti, 2016
	Solidago canadensis L.	Stem<br>Leaves<br>Flower	Yellow	Carotenoids	Briganti, 1842<br>Kerry & David, 2001<br>Angelini et al., 2010
	Aster amellus L.	Stem<br>Dry flower	Yellow	Carotenoids	Briganti, 1842
	Callistephus chinensis (L.) Nees	Leaves<br>Stem	Yellow	Carotenoids	Briganti, 1842

	Inula helenium L.	Roots	Blue	Indigoidines	Briganti, 1842
	Bidens tripartita L.	Leaves<br>Stem	Yellow	Carotenoids	Ucria, 1789<br>Briganti, 1842
	Helianthus annuus L.	Flower	Yellow	Carotenoids	Briganti, 1842<br>Patil et al., 2019
	Xanthium strumarium L.	Young branches<br><br>Grass<br>Fruit<br>Seeds	Red<br><br>Yellow	PAHs, Carotenoids,<br>Anthocyanins	Ucria, 1789<br>Briganti, 1842
	Tagetes erecta L	Fresh flower<br>Leaves<br>Flowery stem	Yellow	Carotenoids	Briganti, 1842<br>Patil et al., 2019
	Artemisia vulgaris L.	Stem	Yellow	Carotenoids	Briganti, 1842
	Artemisia absinthium L.	Pulverized dry stem	Yellow	Carotenoids	Briganti, 1842
	Artemisia dracunculus L.	Woody stem	Yellow	Carotenoids	Briganti, 1842
	Matricaria chamomilla L.	Flower	Yellow	Carotenoids	Briganti, 1842<br>Guarrera, 2006(a)<br>Guarrera, 2006(b)<br>Caneva et al., 2013
	Anthemis cotula L.	Leaves<br>Stem	Yellow	Carotenoids	Briganti, 1842
	Cota tinctoria (L.) J. Gay subsp. *tinctoria*	Grass<br>Flower	Yellow	Carotenoids	Ucria, 1789<br>Briganti, 1842<br>Guarrera, 2006(b)<br>Caneva et al., 2013<br>Kakhia, 2015
	Tanacetum vulgare L.	Leaves<br>Flowery stem	Yellow	Carotenoids	Briganti, 1842<br>Kakhia, 2015
	Glebionis coronaria (L.) N.N.Tzvel.	Whole plant	Yellow	Carotenoids	Guarrera, 2006(b)
	Glebionis segetum (L.) Fourr.	Whole plant	Yellow	Carotenoids	Briganti, 1842
	Arnica montana L.	Leaves	Yellow	Carotenoids	Briganti, 1842
	Jacobaea paludosa (L.) G.Gaertn., B.Mey. & Scherb.	Flowery stem	Yellow	Carotenoids	Briganti, 1842
	Jacobaea vulgaris Gaertn.	Leaves<br>Flowery stem	Green	Clorophylls	Ucria, 1789<br>Briganti, 1842
	Calendula officinalis L.	Petals	Yellow	Carotenoids	Briganti, 1842

		Flowery grass			Guarrera, 2006(b)
	Calendula arvensis L.	Petals<br>Flowery grass	Yellow	Carotenoids	Briganti, 1842<br>Guarrera, 2006(a)<br>Guarrera, 2006(b)
	Arctium lappa L.	Roots<br>Leaves	Yellow	Carotenoids	Briganti, 1842
	Cynara cardunculus L.	Leaves	Green	Clorophylls	Guarrera, 2006(b)<br>Caneva et al., 2013
	Cynara scolymus L.	Leaves	Yellow	Carotenoids	Briganti, 1842<br>Guarrera, 2006(b)
	Serratula tinctoria L.	Stem<br>Dry leaves<br>Flower	Yellow	Carotenoids	Briganti, 1842<br>Cardon, 2010
	Centaurea cyanus L.	Flower	Blue	Indigoidines	Ucria, 1789<br>Briganti, 1842
	Centaurea jacea L.	Grass	Yellow	Carotenoids	Briganti, 1842
	Centaurea nigra L.	Leaves<br>Stem	Green	Clorophylls	Briganti, 1842
	Centaurea benedicta (L.) L.	Leaves	Yellow	Carotenoids	Briganti, 1842
	Centaurea solstitialis L.	Leaves	Yellow	Carotenoids	Briganti, 1842
	Carthamus tinctorius L.	Flower<br><br>Stamens	Red<br><br>Yellow	PAHs, Carotenoids,<br>Anthocyanins<br>Carotenoids	Ucria, 1789<br>Briganti, 1842<br>Zanoni & Schofield, 1983<br>Kerry & David, 2001<br>Guarrera, 2006(a)<br>Guarrera, 2006(b)<br>Angelini et al., 2010<br>Caneva et al., 2013<br>Kakhia, 2015<br>Patil et al., 2019
	Carthamus lanatus L.	Flower	Yellow	Carotenoids	Briganti, 1842<br>Guarrera, 2006(b)
	Cichorium intybus L.	Grass	Green	Clorophylls	Briganti, 1842
	Scorzonera hispanica L.	Roots	Grey	PAHs, Tannins	Briganti, 1842
	Chondrilla juncea L.	Stem<br>Flower	Yellow	Carotenoids	Briganti, 1842

	Sonchus oleraceus L.	Leaves<br>Flowery stem	Yellow	Carotenoids	Briganti, 1842
	Hieracium umbellatum L.	Grass	Yellow	Carotenoids	Ucria 1789<br>Briganti, 1842
**Adoxaceae**					
	Sambucus ebulus L.	Ripe berries	Purple	Anthocyanins	Briganti, 1842<br>Guarrera, 2006(b)
	Sambucus nigra L.	Bark<br>Branches<br>Dry flowers<br>Ripe berries<br>Wood<br>Seed	Yellow<br><br><br>Purple<br>Brown<br>Black	Carotenoids<br><br><br>Anthocyanins<br>Tannins<br>Anthocyanins, Tannins, PAHs	Briganti, 1842<br>Guarrera, 2006(b)
	Sambucus racemosa L.	Leafy branches	Yellow	Carotenoids	Briganti, 1842
	Viburnum lantana L.	Ripe berries	Black	Anthocyanins, Tannins, PAHs	Briganti, 1842
	Viburnum opulus L.	Ripe berries<br>Green branches	Red	PAHs, Carotenoids, Anthocyanins	Briganti, 1842
	Viburnum tinus L.	Green branches	Red	PAHs, Carotenoids, Anthocyanins	Briganti, 1842<br>Guarrera, 2006(b)
**Dipsacaceae**					
	Dipsacus fullonum L.	Roots	Grey	PAHs, Tannins	Briganti, 1842
	Knautia arvensis (L.) Coult.	Leaves<br>Dry flower	Yellow	Carotenoids	Briganti, 1842
