# Supplementary material for: Ethnobotany of dye plants in Southern Italy, Mediterranean Basin: floristic catalog and two centuries of analysis of traditional botanical knowledge heritage
Source: J Ethnobiol Ethnomed. 2020 Jun 3;16:31. doi: 10.1186/s13002-020-00384-2 (PMC7268309; doi:10.1186/s13002-020-00384-2)
Supplement: Supplementary file 3 — Additional file 3: Supplementary Table 2. Dye fungi and lichens catalog. [file 13002_2020_384_MOESM3_ESM.pdf]

Table 2 - Dye fungi and lichens catalogue.

Family and Latin name		Part/s used	Color	References
**Acarosporaceae**				
	Pleopsidium flavum (Bellardi) Körb.	Whole plant	Yellow	Briganti, 1842
**Agaricaceae**				
	Agaricus auricolor V. Brig.	Juice	Brown	Briganti, 1842
	Coprinus comatus (O. F. Müll.) Pers.	Juice	Black	Briganti, 1842
	Lycoperdon perlatum Pers.	Juice	Brown	Guarrera, 2006(b)
	Lycoperdon pyriforme Schaeff.	Juice	Brown	Guarrera, 2006(b)
**Bostrichidae**				
	Lichenophanes plicatus Guérin-Méneville	Whole plant	Yellow	Briganti, 1842
**Chrysothricaceae**				
	Chrysothrix candelaris (L.) J. R. Laundon	Whole plant	Green	Briganti, 1842
**Cladoniaceae**				
	Cladonia fimbriata (L.) Fr.	Whole plant	Red	Briganti, 1842
	Cladonia gracilis (L.) Willd.	Whole plant	Grey	Briganti, 1842
	Cladonia rangiferina (L.) Weber ex F.H.Wigg.	Whole plant	Red	Briganti, 1842
	Cladonia uncialis (L.) Weber ex F.H. Wigg.	Whole plant	Grey	Briganti, 1842
	Cladonia weymouthii F. Wilson ex A. W. Archer	Whole plant	Red	Briganti, 1842
**Fistulinaceae**				
	Fistulina hepatica (Schaeff.) With.	Juice	Red	Briganti, 1842
**Fomitopsidaceae**				
	Fomitopsis officinalis (Vill.) Bondartsev & Singer	Whole plant	Black	Briganti, 1842
	Laetiporus sulphureus (Bull.) Murrill	Whole plant	Yellow	Briganti, 1842
**Graphidaceae**				
	Diploschistes muscorum (Scop.) R. Sant.	Whole plant	Red	Briganti, 1842
	Diploschistes scruposus (Schreb.) Norman	Whole plant	Purple	Briganti, 1842
**Hydnaceae**				
	Hydnum eleosma Pers.	Whole plant	Brown	Briganti, 1842
**Hymenochaetaceae**				
	Phellinus igniarius (L.) Quél.	Whole plant	Brown	Briganti, 1842
**Lecanoraceae**				
	Lecanora poeltiana Clauzade & Cl.Roux	Whole plant	Red	Guarrera, 2006(b)
**Lecideaceae**				
	Lecidea lapicida (Ach.) Ach.	Whole plant	Grey	Guarrera, 2006(b)
**Lobariaceae**				
	Pseudocyphellaria crocata (L.) Vain.	Whole plant	Red	Briganti, 1842
**Ochrolechiaceae**				
	Ochrolechia parella (L.) A. Massal.	Whole plant	Red	Briganti, 1842 Guarrera, 2006(b)

				Caneva et al., 2013
	Ochrolechia tartarea (L.) A. Massal.	Whole plant	Red	Briganti, 1842 Guarrera, 2006(b) Caneva et al., 2013
	Varicellaria lactea (L.) Schmitt & Lumbsch	Whole plant	Purple	Briganti, 1842
**Parmeliaceae**				
	Arctoparmelia centrifuga (L.) Hale	Whole plant	Yellow	Briganti, 1842
	Bryoria chalybeiformis (L.) Brodo & D. Hawksw.	Whole plant	Red	Briganti, 1842
	Cetraria islandica (L.) Ach.	Whole plant	Yellow	Briganti, 1842
	Evernia prunastri (L.) Ach.	Whole plant	Red	Briganti, 1842 Guarrera, 2006(b)
	Flavocetraria nivalis (L.) Kärnefelt & A. Thell	Whole plant	Purple	Briganti, 1842
	Flavoparmelia caperata (L.) Hale	Whole plant	Red	Briganti, 1842
	Hypogymnia physodes Schwäbisch-Fränkische Waldberge	Whole plant	Grey	Briganti, 1842
	Lasallia pustulata (L.) Mérat	Whole plant	Yellow	Briganti, 1842 Guarrera, 2006(b)
	Letharia vulpina (L.) Hue	Whole plant	Yellow	Briganti, 1842
	Lobaria pulmonaria (L.) Hoffm.	Whole plant	Brown	Briganti, 1842
	Melanelia stygia (L.) Essl.	Whole plant	Red	Briganti, 1842
	Melanohalea olivacea (L.) O. Blanco, A. Crespo, Divakar, Essl., D. Hawksw. & Lumbsch	Whole plant	Red	Briganti, 1842
	Parmelia saxatilis (L.) Ach.	Whole plant	Red	Briganti, 1842 Caneva et al., 2013
	Platismatia glauca (L.) W. L. Culb. & C. F. Culb.	Whole plant	Grey	Briganti, 1842
	Usnea florida (L.) Weber ex F. H. Wigg.	Whole plant	Purple	Briganti, 1842
	Usnea hirta (L.) Weber ex F. H. Wigg.	Whole plant	Yellow	Briganti, 1842
	Vulpicida juniperinus (L.) J.-E. Mattsson & M. J. Lai	Whole plant	Yellow	Briganti, 1842
**Patellariaceae**				
	Patellaria paschalis (L.) Wallr.	Whole plant	Grey	Briganti, 1842
**Peltigeraceae**				
	Peltigera canina (L.) Willd.	Whole plant	Red	Briganti, 1842
	Solorina saccata (L.) Ach.	Whole plant	Green	Briganti, 1842
**Pertusariaceae**				
	Pertusaria corallina (L.) Arnold	Whole plant	Unknown	Guarrera, 2006(b)
	Pertusaria lactescens Mudd	Whole plant	Unknown	Guarrera, 2006(b)
**Physciaceae**				
	Buellia spuria (Schaer.) Anzi	Whole plant	Red	Guarrera, 2006(b)

	Dimelaena oreina (Ach.) Norman	Whole plant	Purple	Briganti, 1842
	Physcia tenella (Scop.) DC.	Whole plant	Yellow	Briganti, 1842
**Polyporaceae**				
	Fomes fomentarius (L.) Fr.	Whole plant	Black	Briganti, 1842
	Neofavolus alveolaris (DC.) Sotome & T. Hatt.	Whole plant	Yellow	Briganti, 1842
**Psathyrellaceae**				
	Coprinellus micaceus (Bull.) Vilgalys, Hopple & Jacq. Johnson	Juice	Black	Briganti, 1842
	Coprinopsis atramentaria (Bull.) Redhead, Vilgalys & Moncalvo	Juice	Black	Briganti, 1842
**Ramalinaceae**				
	Ramalina capitata var. *tinctoria* (Hoffm.) Motyka	Whole plant	Purple	Briganti, 1842
	Ramalina farinacea (L.) Ach.	Whole plant	Red	Briganti, 1842
	Ramalina fraxinea (L.) Ach.	Whole plant	Grey	Briganti, 1842
	Ramalina siliquosa var. scopulorum (Retz.) A.E. Wade	Whole plant	Purple	Briganti, 1842
	Ramalina sinensis Jatta	Whole plant	Red	Briganti, 1842
**Roccellaceae**				
	Roccella fuciformis (L.) DC.	Whole plant	Purple	Caneva et al., 2013
	Roccella phycopsis Ach.	Whole plant	Purple	Caneva et al., 2013
	Roccella tinctoria DC.	Whole plant	Purple	Briganti, 1842 Guarrera, 2006(b) Caneva et al.,2013
**Russulaceae**				
	Lactarius chrysorrheus Fr.	Juice	Yellow	Briganti, 1842
	Lactarius necator (Bull.) Pers.	Juice	Yellow	Briganti, 1842
**Suillaceae**				
	Suillus viscidus (L.) Roussel	Whole plant	Green	Briganti, 1842
**Teloschistaceae**				
	Calopaca lactea (A.Massal.) Zahlbr.	Whole plant	Yellow	Guarrera, 2006(b)
	Xanthoria candelaria (L.) Th. Fr.	Whole plant	Yellow	Briganti, 1842
	Xanthoria parietina (L.) Beltr.	Whole plant	Yellow	Briganti, 1842
**Tremellaceae**				
	Tremella mesenterica Retz.	Whole plant	Purple	Briganti, 1842
**Umbilicariaceae**				
	Umbilicaria deusta (L.) Baumg.	Whole plant	Purple	Briganti, 1842
**Verrucariaceae**				
	Dermatocarpon miniatum (L.) W. Mann	Whole plant	Grey	Briganti, 1842
	Verrucaria aethiobola Wahlenb.	Whole plant	Red	Briganti, 1842
