# Supplementary material for: Ethnobotany of dye plants in Southern Italy, Mediterranean Basin: floristic catalog and two centuries of analysis of traditional botanical knowledge heritage
Source: J Ethnobiol Ethnomed. 2020 Jun 3;16:31. doi: 10.1186/s13002-020-00384-2 (PMC7268309; doi:10.1186/s13002-020-00384-2)
Supplement: Supplementary file 4 — Additional file 4: Supplementary Table 3. Dye algae catalog. [file 13002_2020_384_MOESM4_ESM.pdf]

Table 3 - Dye algae catalogue.

Family and Latin name		Part\s used	Color	References
**Callithamniaceae**				
	Ptilota gunneri P.C.Silva, Maggs & L.M.Irvine in Maggs & Hommersand	Whole plant	Purple	Briganti, 1842
**Cystocloniaceae**				
	Cystoclonium purpureum (Hudson) Batters	Whole plant	Purple	Briganti, 1842
**Delesseriaceae**				
	Cryptopleura ramosa (Hudson) L.Newton	Whole plant	Purple	Briganti, 1842
	Delesseria sanguinea (Hudson) J.V.Lamouroux	Whole plant	Purple	Briganti, 1842
	Membranoptera alata (Hudson) Stackhouse	Whole plant	Purple	Briganti, 1842
	Phycodrys rubens (L.) Batters	Whole plant	Purple	Briganti, 1842
**Fucaceae**				
	Ascophyllum nodosum (L.) Le Jolis	Whole plant	Green	Briganti, 1842
	Fucus vesiculosus L.	Whole plant	Green	Briganti, 1842
**Gelidiaceae**				
	Gelidium versicolor (S.G.Gmelin) J.V.Lamouroux	Whole plant	Purple	Briganti, 1842
**Palmariaceae**				
	Palmaria palmata (L.) F.Weber & D.Mohr	Whole plant	Purple	Briganti, 1842
**Plocamiaceae**				
	Plocamium cartilagineum (L.) P.S.Dixon	Whole plant	Purple	Briganti, 1842
**Trentepohliaceae**				
	Trentepohlia jolithus (L.) Wallroth	Whole plant	Green	Briganti, 1842
